# Modeling HNF1B-associated monogenic diabetes using human iPSCs reveals an early stage impairment of the pancreatic developmental program

**DOI:** 10.1016/j.stemcr.2021.07.018

**Published:** 2021-08-26

**Authors:** Ranna El-Khairi, Evelyn Olszanowski, Daniele Muraro, Pedro Madrigal, Katarzyna Tilgner, Mariya Chhatriwala, Sapna Vyas, Crystal Y. Chia, Ludovic Vallier, Santiago A. Rodríguez-Seguí

**Affiliations:** 1 Wellcome Medical Research Council Cambridge Stem Cell Institute, Anne McLaren Laboratory for Regenerative Medicine, University of Cambridge, Cambridge, UK; 2 Wellcome Sanger Institute, Hinxton, Cambridge, UK; 3 Instituto de Fisiología, Biología Molecular y Neurociencias (IFIBYNE), CONICET-Universidad de Buenos Aires, Ciudad Universitaria, Buenos Aires, Argentina; 4 Departamento de Química Biológica, Facultad de Ciencias Exactas y Naturales, Universidad de Buenos Aires, Buenos Aires, Argentina; 5 Departamento de Fisiología, Biología Molecular y Celular, Facultad de Ciencias Exactas y Naturales, Universidad de Buenos Aires, Buenos Aires, Argentina; 6 Department of Surgery, University of Cambridge, Cambridge, UK

**Keywords:** *in vitro*, differentiation, β cell, diabetes, HNF1B, MODY5, monogenic, human induced pluripotent stem cells, iPSC, pancreas

## Abstract

Heterozygous mutations in *HNF1B* in humans result in a multisystem disorder, including pancreatic hypoplasia and diabetes mellitus. Here we used a well-controlled human induced pluripotent stem cell pancreatic differentiation model to elucidate the molecular mechanisms underlying HNF1B-associated diabetes. Our results show that lack of HNF1B blocks specification of pancreatic fate from the foregut progenitor (FP) stage, but HNF1B haploinsufficiency allows differentiation of multipotent pancreatic progenitor cells (MPCs) and insulin-secreting β-like cells. We show that HNF1B haploinsufficiency impairs cell proliferation in FPs and MPCs. This could be attributed to impaired induction of key pancreatic developmental genes, including *SOX11*, *ROBO2*, and additional TEAD1 target genes whose function is associated with MPC self-renewal. In this work we uncover an exhaustive list of potential *HNF1B* gene targets during human pancreas organogenesis whose downregulation might underlie HNF1B-associated diabetes onset in humans, thus providing an important resource to understand the pathogenesis of this disease.

## Highlights

• Lack of HNF1B blocks specification of pancreatic fate from the FP stage
• HNF1B haploinsufficiency allows differentiation of MPCs and β-like cells
• HNF1B haploinsufficiency impairs cell proliferation in FPs and MPCs
• Reduced HNF1B levels impair the induction of *SOX11*, *ROBO2*, and TEAD1 targets

## Graphical abstract

**Figure:**
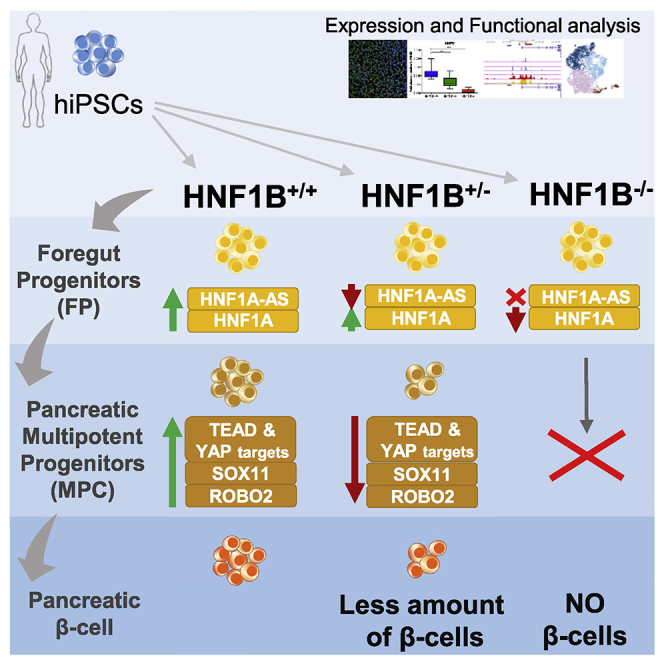

## Teaser

In this article, Rodríguez-Seguí and colleagues use an hiPSC pancreatic differentiation model to study the mechanisms underlying HNF1B-associated diabetes. The authors show that lack of HNF1B blocks specification of pancreatic fate from the foregut progenitor (FP) stage. HNF1B haploinsufficiency, on the other hand, allows differentiation of multipotent pancreatic progenitor cells (MPCs) and insulin-secreting β-like cells, but impairs cell proliferation in FPs and MPCs.

## Introduction

Maturity-onset diabetes of the young (MODY) is the most common form of monogenic diabetes, and it is characterized by autosomal dominant inheritance, onset typically before 25 years of age, and hyperglycemia due to β cell failure. In particular, hepatic nuclear factor 1β (HNF1B), associated with MODY5 (Horikawa et al., 1997), plays an important role in the normal development of the kidney, liver, pancreas, bile ducts, and urogenital tract, through tissue-specific regulation of gene expression in these organs (Barbacci et al., 1999; Coffinier et al., 1999). In humans, heterozygous mutations in *HNF1B* result in a multisystem disorder. The most common clinical features include renal disease, pancreatic hypoplasia, and diabetes mellitus, which typically develops during adolescence or early adulthood.

More than 50 splice-site, nonsense, missense, and frameshift mutations in the *HNF1B* gene have been reported to date, as well as partial or whole gene deletions (Clissold et al., 2014). Patients with whole-gene deletions do not exhibit a phenotype different from those with coding or splice-site mutations, thus suggesting that dysfunction is due to a gene-dosage effect, i.e., haploinsufficiency (Bellanne-Chantelot et al., 2005; Edghill et al., 2006b). Haploinsufficiency is an important contributor to human disease; however, the mechanism by which a reduced dosage of a transcription factor affects downstream target genes to cause a disease is poorly understood, mostly due to the lack of an appropriate model system.

The pathophysiology of diabetes mellitus in patients with *HNF1B* mutations is mainly attributed to β cell dysfunction and reduced insulin secretion, which is likely to be a consequence of pancreatic hypoplasia. Interestingly, mouse models often do not recapitulate the disease phenotype in humans. As an example, mice with heterozygous deletions of *Hnf1a*, *Hnf4a*, or *Hnf1b* do not develop diabetes (El-Khairi and Vallier, 2016; Harries et al., 2009; Lau et al., 2018). This species divergence and the difficulty in accessing patient samples led us to model HNF1B deficiency *in vitro* using human pluripotent stem cells (hPSCs). Several studies in the past decade have used genetically engineered hPSC culture systems for differentiation into pancreatic cells to further expand our understanding of the roles of various genes in pancreas development and function (recently reviewed in Burgos et al., 2021).

In this study, we established a well-controlled human induced pluripotent stem cell (hiPSC) pancreatic differentiation model to elucidate the molecular mechanisms underlying HNF1B-associated diabetes and pancreatic hypoplasia. We generated isogenic HNF1B mutant lines to investigate the influence of HNF1B dosage on pancreatic differentiation. Our findings reveal that homozygous knockout of *HNF1B* resulted in failure of foregut and pancreatic progenitor development. Heterozygous knockout of *HNF1B*, on the other hand, resulted in impairment of pancreatic progenitor and endocrine cell production. Despite the lower efficiency in producing β-like cells, these were functional to the same extent as their counterparts derived from wild-type (WT) hiPSCs. RNA sequencing (RNA-seq) and in-depth transcriptomic analyses showed that low dosages of HNF1B in pancreatic progenitor cells alter their early stage specification, downregulating the expression of several genes with known or suspected roles in pancreas development. Our results are consistent with a model in which HNF1B haploinsufficiency impairs the expansion and maintenance of pancreatic progenitor cells *in vitro*. *In vivo*, this would likely result in reduced β cell numbers at birth and increased diabetes susceptibility later in life.

## Results

### HNF1B is expressed during the *in vitro* differentiation of human iPSCs into the pancreatic lineage

**Figure 1:**
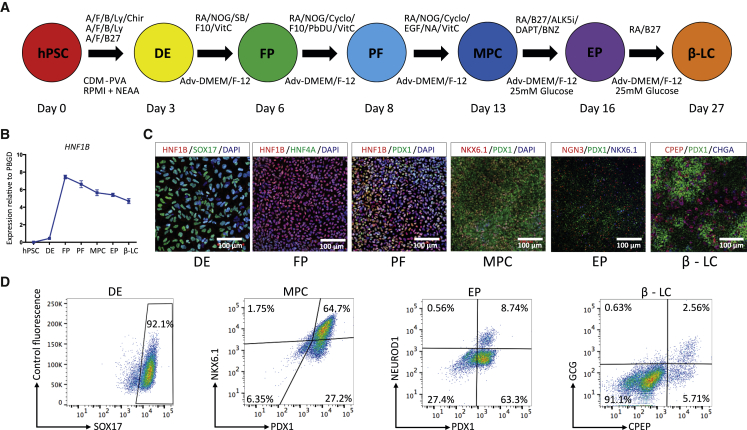
HNF1B expression during hiPSC pancreatic differentiation (A) Overview of the protocol used to differentiate hiPSCs into pancreatic β-like cells. hPSC, human induced pluripotent stem cell; DE, definitive endoderm, FP, foregut progenitor; PF, posterior foregut; MPC, multipotent pancreatic progenitor cell; EP, endocrine progenitor; β-LC, β-like cell. A, activin A; F, fibroblast growth factor 2; B, bone morphogenetic protein; CDM, chemically defined medium. Refer to the supplemental experimental procedures for additional abbreviations. (B) The mRNA expression pattern for *HNF1B* during hPSC differentiation into pancreatic β-like cells. The mRNA levels were measured by qRT-PCR (n = 5 independent experiments at each stage of differentiation using the FSPS13.B wild-type clone) and normalized to the housekeeping gene *PBGD*. (C) Representative immunostaining of HNF1B and other stage-specific markers. (D) Representative FACS dot plots of cells stained for the stage-specific markers SOX17, PDX1, NKX6.1, NEUROD1, CPEP, and GCG. The percentage of each cell population is indicated in the corresponding quadrant for all FACS plots.

Directed differentiation of hiPSCs into pancreatic cells was undertaken using a protocol developed in our laboratory (Figure 1A). This 27-day protocol, a revised version of an 18-day chemically defined protocol previously published by our group (Cho et al., 2012), was applied using two hiPSC lines (FSPS13.B and Eipl_1). Consistent with findings using our previous protocol, HNF1B expression was upregulated at the foregut progenitor (FP) stage (Figure 1B) and was co-expressed with other FP markers such as HNF4A (Figures 1C and S1A–S1C, day 6). At the posterior foregut stage, PDX1^+^ cells co-expressing HNF1B were identified, but NKX6.1 was not detected (Figures 1C and S1A–S1C, day 8). At the pancreatic multipotent progenitor cell (MPC) stage, >90% PDX1^+^ cells were detected, and HNF1B was still co-expressed with PDX1 in almost all cells, and around 50% of cells co-expressed NKX6.1 (∼60% in FSPS13.B and ∼40% in Eipl_1) (Figures 1C, 1D, S1A–S1C and data not shown). At the endocrine progenitor (EP) and β-like cell stages the expression of HNF1B was decreased (Figure S1C). Consistent with these findings, Hnf1b expression is excluded from β cells at comparable stages in adult mice and is restricted to adult ductal cells (Haumaitre et al., 2005; Maestro et al., 2003; Nammo et al., 2008). Expression of the EP cell markers NGN3 and NEUROD1 peaked at day 16 (EP) and around 8%–10% of the cells expressed NEUROD1 at this stage (Figures 1C and 1D). Expression of pancreatic hormonal markers (chromogranin A, insulin, glucagon, and somatostatin) significantly increased at day 27. By day 27 around 7%–8% of the cells expressed CPEP and around 50%–60% of these cells were monohormonal (4%–5% of total cells) (Figures 1C, 1D, S1A–S1C). Taken together, these results show that our *in vitro* protocol of cell differentiation follows a natural path of development with HNF1B expression starting at the foregut stage.

### HNF1B is required for efficient foregut and pancreatic progenitor formation

**Figure 2:**
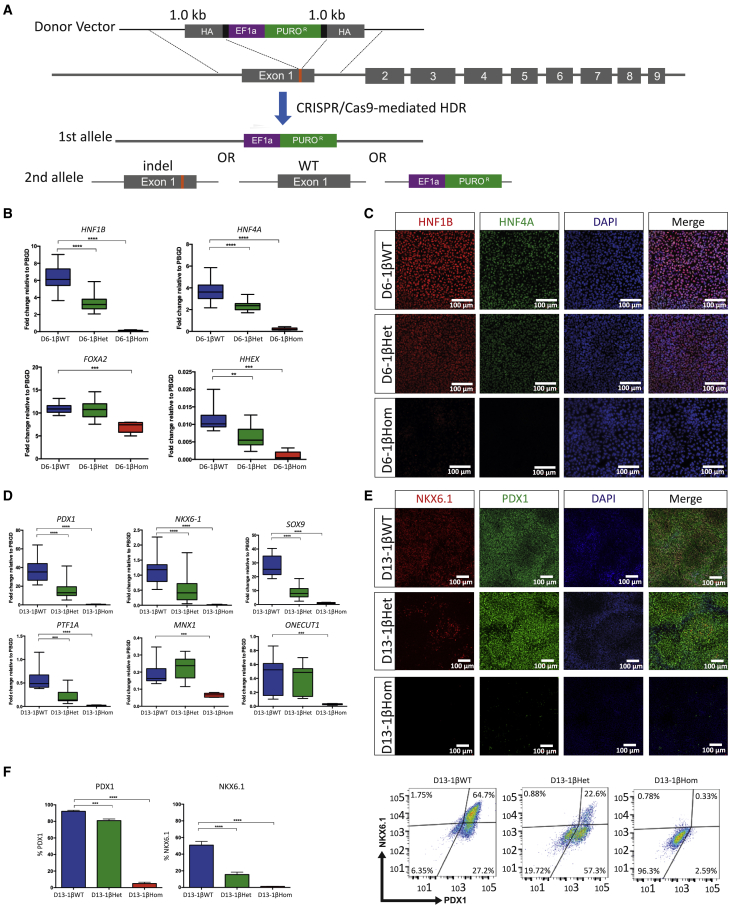
Derivation and characterization of HNF1B mutant hiPSC lines (A) CRISPR guide RNA design for generating HNF1B mutants from the FSPS13.B parental line. The schematic shows the human *HNF1B* locus and indicates the CRISPR-Cas9 cut site, lying in exon 1. The target sequences of the CRISPR guide RNA and the corresponding protospacer-adjacent motif sequence are indicated in black and red, respectively. The “knock-in” vector introduces a puromycin-resistance cassette. Successful homologous recombination resulted in both heterozygous HNF1B^+/−^ and homozygous HNF1B^−/−^ mutant hiPSCs. (B) Expression of *HNF1B*, *HNF4A*, *FOXA2*, and *HHEX* at the foregut progenitor stage of differentiation. The mRNA levels were measured by qRT-PCR and normalized to the housekeeping gene *PBGD*. Data were pooled from n = 5 independent experiments for each of the eight FSPS13.B clones and n = 3 independent experiments for each of the eight Eipl_1 clones, with clone identities as per Figure S2C. Student's t test with two-tailed distribution was used for statistical analysis. All data are presented as the mean ± SEM. ^∗∗^p < 0.01, ^∗∗∗^p < 0.001, and ^∗∗∗∗^p < 0.0001. (C) Representative immunofluorescence images showing wild-type HNF1B^+/+^ (D6-1βWT), heterozygous HNF1B^+/−^ (D6-1βHet), and homozygous HNF1B^−/−^ (D6-1βHom) mutant cells at the foregut progenitor stage of differentiation. (D) Expression of key pancreatic developmental genes in D13-1βWT, D13-1βHet, and D13-1βHom cells. The mRNA levels were measured by qRT-PCR and normalized to the housekeeping gene *PBGD*. Replicates and statistics are as indicated for (B). (E) Representative immunostaining showing PDX1 and NKX6.1 co-expression at the pancreatic progenitor stage of differentiation in D13-1βWT, D13-1βHet, and D13-1βHom cells. (F) FACS analysis of PDX1^+^, NKX6.1^+^ pancreatic progenitor cells derived from 1βWT, 1βHet, and 1βHom hiPSCs at day 13 of the differentiation protocol and representative plots. Replicates and statistics are as indicated for (B).

We then used the CRISPR-Cas9 genome editing system to generate homozygous and heterozygous HNF1B knockout hiPSCs (Figure 2A). Insertion of a puromycin-resistance cassette allowed for reliable selection of targeted clones and resulted in large disruptions in the open reading frame of the *HNF1B* gene in one or both alleles (Figures 2A, S2A, and S2B, Table S1). To control for potential CRISPR-Cas9 off-target effects and line-to-line variations, we analyzed six HNF1B heterozygous and four HNF1B homozygous hiPSCs for the FSPS13.B and Eipl_1 hiPSC lines, and compared them with six isogenic HNF1B WT control hiPSC lines. Notably, the expression of the pluripotency markers *NANOG*, *OCT4*, and *SOX2* was not affected in the hiPSCs edited with this approach (Figure S2C). Western blotting confirmed the absence of HNF1B protein in homozygous mutant lines at the FP stage (Figure S2D). We also detected reduced HNF1B expression in HNF1B heterozygous cells at the FP stage by qRT-PCR and immunostaining (Figures 2B, 2C, and S2E). Hereafter, we will refer to cells derived from the WT (HNF1B^+/+^), heterozygous (HNF1B^+/−^), and homozygous (HNF1B^−/−^) mutant lines as 1βWT, 1βHet, and 1βHom, respectively, preceded by the differentiation stage day. Thus, D13-1βWT stands for day 13 cells derived from WT (HNF1B^+/+^) hiPSCs, and so on.

A more detailed analysis of the differentiation outcomes for these cells revealed that D3-1βWT, D3-1βHet, and D3-1βHom hiPSCs could be differentiated into definitive endoderm cells expressing *SOX17* and *CXCR4* with comparable efficiencies, as determined by qRT-PCR (Figure S2F) and fluorescence-activated cell sorting (FACS) analysis for SOX17^+^ cells (Figure S2G). Subsequently, D6-1βHom mutant lines failed to differentiate to FP (HNF1B^+^/HNF4A^+^) cells, while HNF1B and HNF4A expression was significantly reduced in D6-1βHet cells compared with D6-1βWT cells (Figures 2B and 2C). Consistent with this, PDX1^+^/NKX6.1^−^ and PDX1^+^/NKX6.1^+^ cells failed to form in D8-1βHom and D13-1βHom cells, respectively (Figures 2D and 2E and data not shown). This is likely due to the earlier requirement for HNF1B at the FP stage. On the other hand, D13-1βHet mutant lines produced a lower number of PDX1^+^/NKX6.1^+^ pancreatic progenitor cells. Other pancreatic progenitor markers, such as *SOX9*, *PTF1A*, and *HNF6/ONECUT1*, also showed statistically significantly reduced expression levels between D13-1βHet and D13-1βWT cells (Figure 2D). These data demonstrate that HNF1B is essential for the efficient formation of posterior foregut, while a decrease in its expression affects production of pancreatic cells.

### HNF1B haploinsufficiency impairs the formation but not the functionality of β-like cells

**Figure 3:**
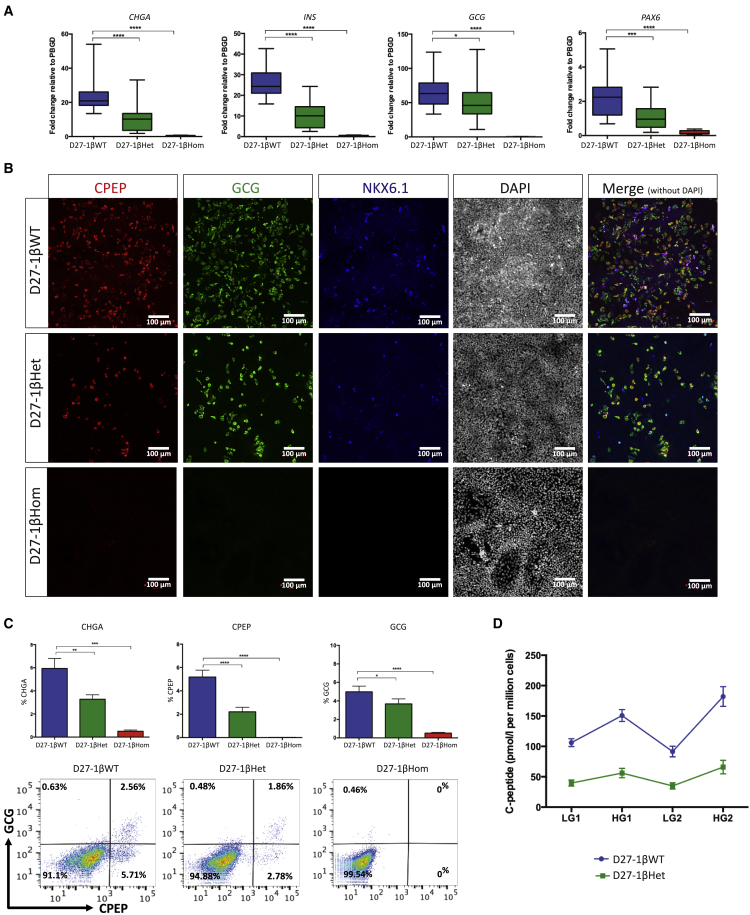
HNF1B haploinsufficiency impairs β-like cell differentiation (A) Expression of *CHGA*, *INS*, *GCG*, and *PAX6* in D27-1βWT, D27-1βHet, and D27-1βHom cells. The mRNA levels were measured by qRT-PCR and normalized to the housekeeping gene *PBGD*. Data were pooled from n = 5 independent experiments for each of the eight FSPS13.B clones and n = 3 independent experiments for each of the eight Eipl_1 clones, with clone identities as per Figure S2C. Student's t test with two-tailed distribution was used for statistical analysis. All data are presented as the mean ± SEM unless otherwise indicated. ^∗^p < 0.05, ^∗∗^p < 0.01, ^∗∗∗^p < 0.001, and ^∗∗∗∗^p < 0.0001. (B) Representative immunofluorescence images showing CPEP, GCG, and NKX6.1 co-staining in D27-1βWT, D27-1βHet, and D27-1βHom cells. (C) Percentage of cells expressing CHGA, CPEP, and GCG and representative FACS dot plots of cells derived from 1βWT, 1βHet, and 1βHom hiPSCs at day 27 of the differentiation protocol stained for CPEP and GCG. Replicates and statistics are as indicated for (A). (D) C-peptide secretion from β-like cells derived from HNF1B^+/+^ (D27-1βWT) and HNF1B^+/−^ (in D27-1βHet) hiPSCs. Cells were incubated in high-glucose (22.5 mmol/L) and low-glucose (2.25 mmol/L) culture medium for two rounds of stimulations. Replicates were as indicated for (A). Error bars indicate SEM.

To investigate the functional consequences of HNF1B haploinsufficiency, we further differentiated cells into EPs and then hormonal/β-like cells. As expected from our previous findings, 1βHom mutant lines failed to form EPs or hormonal cells (Figures 3 and S3). The expression of key EP cell markers such as *NEUROD1* and *NEUROG3* was completely abolished in D16-1βHom cells, while *GLIS3* expression was significantly reduced (Figure S3A). In contrast, *NEUROG3* expression was unaffected in D16-1βHet cells, while *NEUROD1* and *GLIS3* were expressed at lower levels compared with D16-1βWT cells (Figure S3A). By the final stage of the differentiation protocol, D27-1βHet cells showed reduced expression of several endocrine markers important for β cell function (Figures 3A and 3B). Interestingly, 1βHet hiPSCs were still able to form CPEP^+^ hormonal cells, but the percentage was greatly reduced compared with 1βWT cells (2%–3% versus 5%–6%, Figure 3C). Notably, approximately 60%–70% of CPEP^+^ cells were monohormonal for both D27-1βWT and D27-1βHet cells. However, the D27-1βHet mutant clones had fewer CPEP^+^ cells co-expressing NKX6.1 (Figure 3B), which is known to play important roles in maintaining adult β cell function (Taylor et al., 2013).

Functional assays on hiPSC-derived β-like cells showed that D27-1βHet cells exhibited reduced glucose-stimulated insulin secretion (GSIS) compared with D27-1βWT cells (Figure 3D). The total concentration of C-peptide secreted was reduced, but not the ratio of C-peptide secreted in high glucose (22.5 mM) to low glucose (2.25 mM), after correcting for the reduced number of CPEP^+^ β-like cells in 1βHet mutant cells (ratio 1.99 versus 1.91, p = 0.41). This was seen for both FSPS13.B and Eipl_1 hiPSCs. Eipl_1 iPSCs produced reduced numbers of CPEP^+^ cells and GSIS compared with FSPS13.B iPSCs (data not shown). These data confirm that the absence of HNF1B entirely blocks pancreatic development. A decrease in HNF1B expression, on the other hand, appears to affect the production of pancreatic progenitor cells without entirely inhibiting their capacity to differentiate into insulin-secreting β-like cells.

### HNF1B activates key genes at the foregut progenitor and posterior foregut stages to allow specification of pancreatic progenitor cells

To understand the molecular mechanisms underlying how the loss of function of one or both alleles of HNF1B impairs pancreatic progenitor cell development, we used bulk RNA-seq to profile the transcriptomes of 1βWT, 1βHet, and 1βHom hiPSC-derived progenitors at the FP stage (day 6) when HNF1B starts to be significantly expressed, and at posterior foregut (day 8), MPC (day 13), EP (day 16), and hormonal cell/β-like cell (day 27) stages. RNA-seq was profiled from triplicate differentiation experiments for each cell genotype and differentiation stage (Table S2). As expected, the gene expression pattern of key pancreatic differentiation markers was consistent with the results obtained by qRT-PCR (Figure S4).

Principal-component analysis separated 1βWT, 1βHet, and 1βHom cells by day of differentiation in the main component (with PC1 explaining 53% of the variance, Figure S5A). The second principal component explained 20% of the variance and separated cells by genotype. As expected, 1βWT and 1βHet cell lines were transcriptionally more similar to each other than to 1βHom cells. Calculation of the sample-to-sample distance matrix for all samples and replicates followed by unsupervised hierarchical clustering grouped together the sample replicates by day and then by 1βHom genotype, but was not able to clearly cluster 1βWT and 1βHet cells (Figure S5B). These analyses therefore confirm that the absence of HNF1B has a transcriptional impact on pancreas specification from hPSCs at the foregut stage and on, while heterozygous knockouts have a limited but still detectable effect.

**Figure 4:**
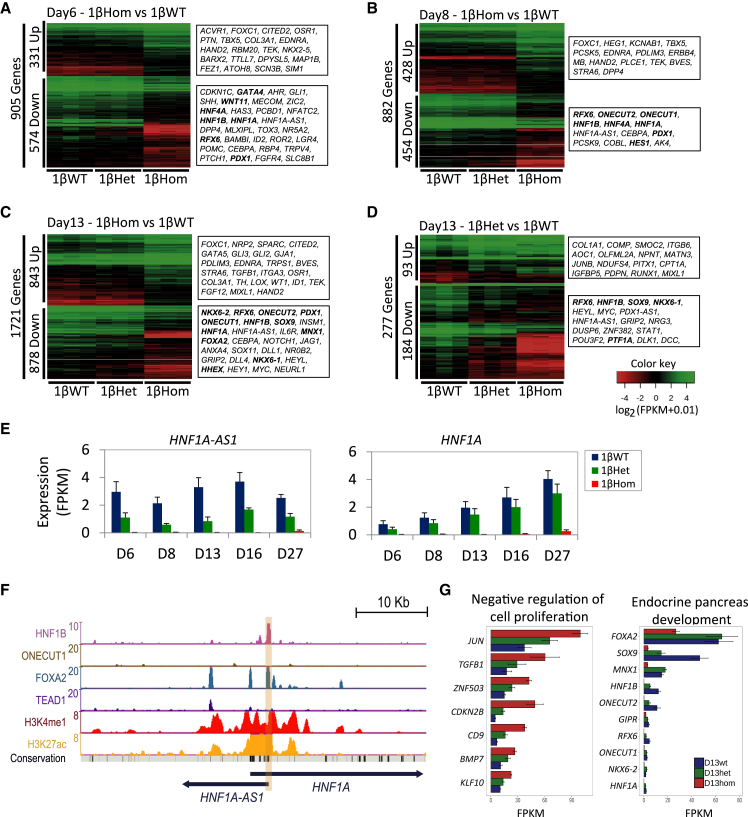
HNF1B activates key genes at the foregut progenitor and posterior foregut stages to allow specification of pancreatic progenitor cells (A–C) Expression of differentially regulated genes between 1βWT and 1βHom for the three genotypes at day 6 (A), day 8 (B), and day 13 (C) of the differentiation protocol. (D) Expression of differentially regulated genes between 1βWT and 1βHet for the three genotypes at day 13 of the differentiation protocol. The bolded genes on the right are known to be important for pancreas development. (E) Expression of *HNF1A-AS1* and *HNF1A* for all genotypes and differentiation stages of the *in vitro* protocol. (F) UCSC genome browser snapshot of the *HNF1A* genomic locus. ChIP-seq was used to locate binding sites of HNF1B, ONECUT1, FOXA2, and TEAD1 in MPCs. ChIP-seq for H3K4me1 and H3K27ac histone modifications denotes the epigenomic printing of active enhancers. HNF1B binding at the *HNF1A-AS1* promoter is highlighted in light orange. (G) Expression levels of a selection of genes from the gene ontology enriched terms “endocrine pancreas development” and “negative regulation of cell proliferation” across the three genotypes.

A more detailed analysis revealed the sets of differentially expressed genes from pairwise comparisons among samples derived from 1βHet and 1βHom cells, compared with their 1βWT counterparts. Consistent with the global analysis described above, we detected a much larger fraction of genes differentially expressed in 1βHom cells than in 1βHet cells (Table S3, Figures 4A–4D and S4B). Interestingly, the early effect of a low HNF1B dosage (in D6-1βHet and D8-1βHet cells) was mainly the downregulation of tens of genes, with almost no genes upregulated in these samples (Table S3). Most of the downregulated genes in 1βHet-derived progenitors at these stages could not be associated with known definitive endoderm/FP functions. We note, however, the consistent downregulation of the HNF1A antisense long non-coding RNA (lncRNA *HNF1A-AS1*) in all 1βHet and 1βHom samples from all stages (Figure 4E). In particular, *HNF1A-AS1* expression was downregulated, but not abolished, in samples derived from 1βHet cells, without impairing *HNF1A* expression. In sharp contrast, *HNF1A-AS1* expression was completely abolished in all samples derived from 1βHom cells, and its associated gene, coding for the transcription factor HNF1A, was not expressed in these cells (Figure 4E). Notably, *HNF1A-AS1* presents active chromatin marks at its promoter and nearby regulatory regions (H3K27ac and H3K4me1) in *in vitro* MPCs (data from our previous study, Cebola et al., 2015) and strong HNF1B and FOXA2 chromatin immunoprecipitation sequencing (ChIP-seq) binding sites at its promoter (Figure 4F). Thus, *HNF1A-AS1* could be one of the earliest HNF1B directly regulated gene targets. We also detect TEAD1 and FOXA2 binding at an active enhancer region upstream of the HNF1A promoter, potentially involved in the regulation of *HNF1A* and/or *HNF1A-AS1*. Other genes downregulated in 1βHom-derived progenitors included well-known pancreatic regulators such as *HNF4A*, *FGFR4*, *HHEX*, *SFRP5*, and *PDX1* (Figures 4A–4D, Table S3). These results suggest that the effect of early HNF1B activation, at days 6 and 8, is mainly the upregulation of a few key specific genes. The numbers of up- and downregulated genes then increase in D13-1βHet, D16-1βHet, and D27-1βHet cells. Taken together, these findings are consistent with an activator role for HNF1B at the FP and posterior foregut stages.

To gain further insights into the molecular pathways controlled by HNF1B in the initial stages of pancreas specification, we functionally annotated the sets of up- and downregulated genes in 1βHet- and 1βHom-derived cells, compared with 1βWT cells. Given that pancreas specification is abolished in 1βHom cells, we characterized in further detail the pathways enriched in these cells from day 6 to day 13. Biological pathway analysis of downregulated genes revealed significant enrichment for terms associated with lipid and retinoic acid metabolism (D6-1βHom and D8-1βHom) and endocrine pancreas development (D13-1βHom, Table S4, Figure 4G). In contrast, upregulated genes were enriched in annotations associated with alternative developmental pathways, notably heart, kidney, and nervous system development. Interestingly, upregulated genes in D13-1βHom cells were enriched in “negative regulation of cell proliferation” (Table S4, Figure 4G), suggesting that a low dosage of HNF1B at this stage could be associated with impairment of MPC population expansion. Despite not finding enrichment for this category in the D13-1βWT versus D13-1βHet cell comparison, we note that the expression of this gene set presents the same downregulation trend as in D13-1βHom samples, although at milder fold changes (Figure 4G). Considered together, these observations show that HNF1B plays a central role in the specification of the foregut toward the pancreatic lineages by controlling key master regulators, while its full expression could be necessary for proliferation of MPCs.

### HNF1B haploinsufficiency impairs cell proliferation in foregut and pancreatic multipotent progenitor cells

**Figure 5:**
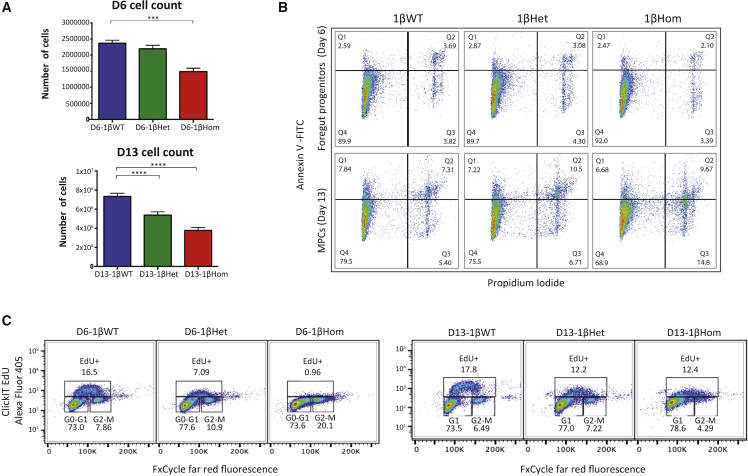
HNF1B haploinsufficiency impairs cell proliferation in foregut and pancreatic multipotent progenitor cells (A) Cell count determined by the number of cells harvested from one well of a 12-well plate of 1βWT, 1βHet, and 1βHom cells taken at the foregut progenitor stage (D6) and pancreatic progenitor stage (D13) of differentiation. n = 3 independent experiments derived from FSPS13.B clones. Student's t test with two-tailed distribution was used for statistical analysis. Data are presented as the mean ± SEM. ^∗∗∗^p < 0.001 and ^∗∗∗∗^p < 0.0001. (B) Apoptosis assay in 1βWT, 1βHet, and 1βHom cells taken at the foregut progenitor stage (D6) and pancreatic progenitor stage (D13) of differentiation. (C) EdU staining showing percentage of cells in G1, S, and G2M phase for plates of 1βWT, 1βHet, and 1βHom cells taken at the foregut progenitor stage (D6) and pancreatic progenitor stage (D13) of differentiation. n = 3 independent experiments derived from FSPS13.B clones.

We next interrogated whether the negative regulation of cell proliferation in D13 progenitor cells could be one of the effects of the lack or low dosages of HNF1B early during pancreas development. Indeed, the numbers of cells harvested at days 6 and 13 were significantly lower in 1βHom cells compared with 1βWT cells (Figure 5A), starting at the FP stage (1.46 × 10^6^ cells versus 2.2 × 10^6^ versus 2.37 × 10^6^, p < 0.05, p = 0.24). The difference was larger, for both 1βHom and 1βHet cells, at the MPC stage (3.78 × 10^6^ versus 5.38 × 10^6^ versus 7.33 × 10^6^, p < 0.05, p < 0.05). To explain the reduction in cell number, we compared the rate of apoptosis and cell proliferation during the differentiation of 1βWT, 1βHet, and 1βHom cells. Apoptosis assays (using propidium iodide and Annexin V staining) performed at the FP and MPC stages showed no significant difference in the number of cells in early or late apoptosis between 1βWT, 1βHet, and 1βHom cell lines (Figure 5B). The number of proliferating cells was next determined using 5-ethynyl-2′-deoxyuridine (EdU) incorporation. A significant reduction in cell proliferation was seen in D6-1βHet and D6-1βHom cells at the FP stage compared with their 1βWT counterparts (Figure 5C). There was a significant decrease in the number of cells in S phase and a corresponding increase in the percentage of cells at G1 and G2/M phase, as non-proliferating cells accumulated at these stages. At the MPC stage, there was a significant decrease in the number of cells in S phase in both D13-1βHet and D13-1βHom compared with D13-1βWT cells. There was no consistent decrease in proliferation among the D13-1βHet and D13-1βHom cells, suggesting that by this stage 1βHom cells have a different identity and respond differently to external stimuli. Taken together, these results suggest that the loss of one functional *HNF1B* allele results in decreased cell proliferation, which impairs the production of pancreatic progenitor cells from foregut cells. This could potentially explain the reduced production of CPEP^+^ β-like cells later on.

### Potential alternative paths for production of endocrine cells from *in vitro*-derived pancreatic progenitor cells at day 13

The results from the previous sections suggest that D13-1βHet cells present a broadly similar gene expression profile (Figures S5A and S5B). Yet, they have impaired cell proliferation and impaired β-like cell production, which are, however, functionally similar to those derived from 1βWT progenitors. To gain a deeper understanding of the mechanisms that allow specification of the pancreatic endocrine lineage in cells with low HNF1B dosages, we performed single-cell RNA-seq (scRNA-seq) in 1βWT and 1βHet cells from day 13, a stage at which the HNF1β dosage on the number of differentially regulated genes became more evident.

**Figure 6:**
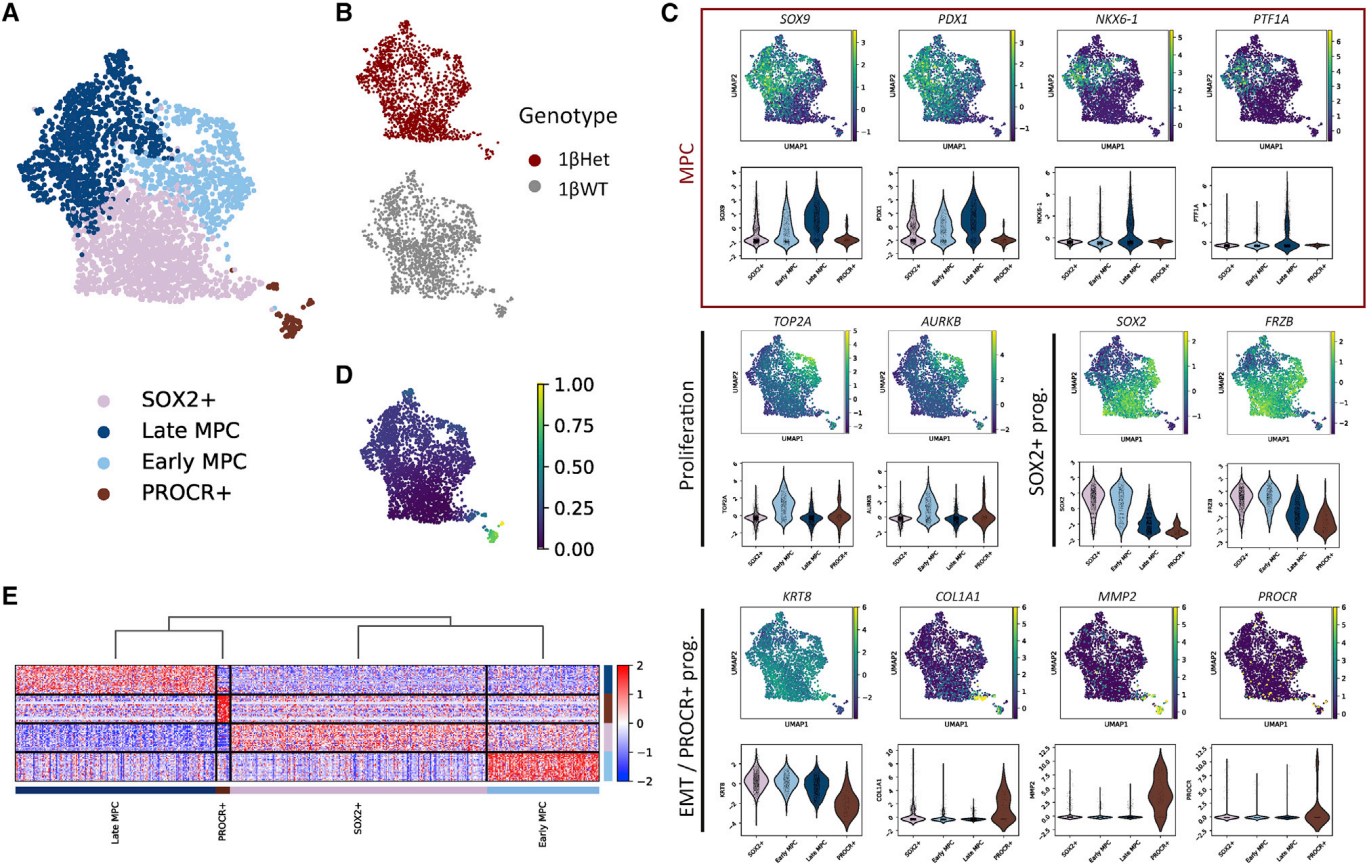
scRNA-seq analysis reveals cell populations derived *in vitro* from 1βWT and 1βHet hiPSCs (A) Uniform manifold approximation and projection (UMAP) plot of 3,216 single-cell transcriptomes profiled from the day 13 differentiation time point (1βWT and 1βHet samples). Colors in the UMAP on the right highlight clustering into four main cell subtypes that were matched with their closest *in vivo* progenitor cell type as described in the main text. (B) UMAP plots showing the distribution of clustered cells colored according to the genotype. (C) Feature and violin plots showing expression of selected progenitor cell genes in human *in vitro*-derived pancreatic cells clustered as in (A). The red rectangle highlights the MPC markers. (D) Pseudotime order of *in vitro*-derived progenitor cells shown in the UMAP plot in (A). (E) Heatmap of the top 50 enriched genes for each cluster. Each column represents a single cell and each row represents one signature gene. The colors ranging from blue to red indicate low to high relative gene expression levels. The dendrogram on top of the heatmap indicates that late MPC and PROCR^+^ cells have closer transcriptional profiles according to these markers.

An unsupervised graph-based clustering allowed identification of four cell clusters, each containing both 1βHet- and 1βWT-derived cells (Figures 6A, 6B, and S6C). Analysis of the combined expression profiles for these clusters matched highly proliferative MPCs (“early MPC”) and less proliferative MPCs (“late MPC”), as recently described for *in vitro* human pancreatic differentiation protocols at a similar differentiation stage (Veres et al., 2019). These cells showed combined expression of MPC markers *PDX1*, *SOX9*, *PTF1A*, *DLK1*, and *NKX6*-*1*, with late MPC having the highest expression for all these markers (Figures 6C and S6A). Late MPCs also expressed higher levels of *CPA2*. We also detected a cluster of progenitor cells with high *SOX2* and *FRZB* expression (“SOX2^+^,” Figures 6A and 6C, Table S5). A similar cluster was also described in a subset of pancreatic progenitor cells derived *in vitro* (Veres et al., 2019) and ascribed to non-endocrine committed progenitors. Notably, SOX2 has been detected early during pancreas specification in the pre-pancreatic gut region and reported to be soon excluded from pancreatic buds (Wilson et al., 2005). These cells also expressed high levels of *SOX21*, which has been previously detected in the mouse developing pancreas (Wilson et al., 2005). Importantly, *SOX2* and *SOX21* expression levels were rapidly downregulated in the other cell clusters, thus suggesting that these cells represent pre-pancreatic gut progenitor cells. As well, SOX2^+^ progenitor cells expressed moderate levels of *PDX1* and *SOX9*, while *NKX6*-*1* and *PTF1A* were barely detected (Figure 6C). The expression of all these markers gradually increased from SOX2^+^ progenitors to early MPC, having the highest levels in late MPC (Figure 6C).

We additionally detected a fourth cluster that presented co-expression of epithelial and mesenchymal markers, including *KRT8*, *COL1A1*, and *MMP2* (Figures 6C, S6A, and S6B), indicative of the epithelial-to-mesenchymal transition (EMT). Interestingly, this cluster also expressed several markers matching a recently reported Procr^+^ progenitor population present in adult mouse pancreatic islets (Wang et al., 2020), including *PROCR*, *SPARC*, and *IGFBP5* (Figure S6A), in addition to co-expressing the EMT markers mentioned above and being *NEUROG3*^−^ (not detected in any of the cell clusters of our scRNA-seq dataset). Notably, Procr^+^ progenitors from adult mouse islets were reported to give rise to all endocrine cells without passing through an Ngn3^+^ cell stage. A pseudotime analysis further supported the progenitor match for our clusters (Figure 6D), showing a differentiation cluster order from SOX2^+^ progenitors either toward PROCR^+^ progenitors or into early MPC and late MPC. Notably, a clustering analysis performed using the top 50 markers for each cell cluster revealed that SOX2^+^ and early MPCs have closer transcriptional profiles, consistent with their early progenitor stage (Figure 6E). Conversely, late MPCs and PROCR^+^ cells were first clustered together, in agreement with the advanced differentiated stage of these cells.

### HNF1B haploinsufficiency impairs the early stage pancreatic developmental program by altering expression of key non-canonical Wnt and Hippo signaling pathway components

**Figure 7:**
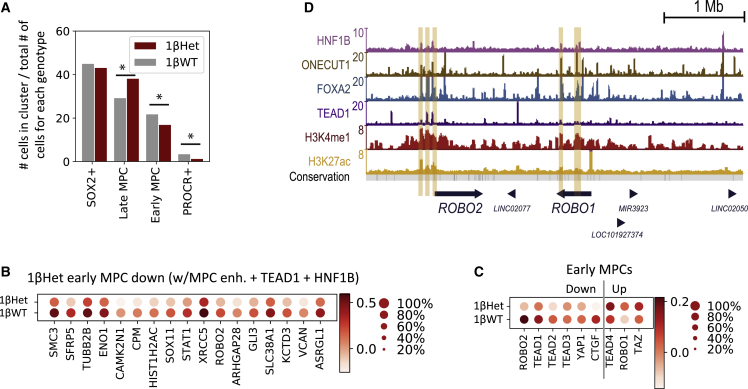
HNF1B haploinsufficiency impairs the early stage pancreatic developmental program by altering expression of key non-canonical Wnt and Hippo signaling pathway components (A) The distribution of clustered cell types by genotype. The numbers of clustered 1βWT and 1βHet cells were normalized independently by genotype; the sum of all red bars accounts for 100% of 1βHet cells and that of the gray bars for 100% of 1βWT cells.∗p < 0.001. (B) Dot plot showing the expression of genes significantly downregulated in 1βWT and 1βHet early MPCs. Genes were filtered by the association with at least one MPC enhancer (as previously defined in Cebola et al., 2015) that presents both TEAD1 and HNF1B ChIP-seq enrichment. Color intensity indicates mean expression (normalized) in a cluster, dot size indicates the proportion of cells in a cluster expressing the gene. (C) Dot plot showing expression of *ROBO1*, *ROBO2*, selected Hippo pathway components, and its known target *CTGF* in 1βWT and 1βHet early MPCs. Color intensity indicates mean expression (normalized) in a cluster, dot size indicates the proportion of cells in a cluster expressing the gene. (D) UCSC genome browser snapshot of the *ROBO1* and *ROBO2* genomic locus. ChIP-seq was used to locate binding sites of HNF1B, ONECUT1, FOXA2, and TEAD1 in MPCs (data from Cebola et al., 2015). ChIP-seq for H3K4me1 and H3K27ac histone modifications denotes the epigenomic printing of active enhancers. MPC enhancers enriched in HNF1B signal in this locus are highlighted in light orange.

We next sought to identify the transcriptional effects of low HNF1B dosages in each cell cluster. The proportion of 1βWT and 1βHet cells (after normalizing by the total number of cells per genotype) did not change for SOX2^+^ progenitors, but low HNF1B dosages switched the balance between early MPC, late MPC, and PROCR^+^ progenitors (Figure 7A). In other words, the number of late MPCs in D13-1βHet samples was higher than in their 1βWT counterparts, and this increase appeared to take place mainly at the expense of the early MPC population. This finding is in agreement with the decreased proliferation in bulk D13-1βHet cell cultures (Figure 5), since early MPCs are highly proliferative progenitors, as evidenced by the increased expression of the proliferation markers *TOP2A* and *AURKB* (Figure S6A). These results suggest that HNF1B plays an important role in allowing the proliferative early MPC stage.

Looking at the genes that are differentially expressed in 1βHet for each cluster revealed significant downregulation of some known and potentially novel pancreatic regulators (Figure S7A, Table S6). In addition to *HNF1B* itself, these included *SOX11* (in the SOX2^+^ progenitor, early MPC and late MPC clusters); *SOX4*, *TEAD1*, *GATA6*, and *HMGA2* (in the SOX2^+^ progenitor cluster); and *ONECUT2* in the early MPC cluster (Cebola et al., 2015; Sarkar et al., 2008; Wilson et al., 2005; Yu et al., 2019). Interestingly, several genes upregulated in 1βHet in the SOX2^+^ progenitor, early MPC, and late MPC cluster cells coded for pancreatic exocrine enzymes, including serine proteases *PRSS1* and *PRSS2* and carboxypeptidase *CPB1*. However, most of the differentially expressed genes in the presence of low HNF1B dosages have unannotated functions in pancreas development.

Given that 1βHet samples had a larger number of late MPCs at the expense of early MPCs, we further inquired the relevance of genes differentially regulated in these clusters in the context of pancreas development. For this purpose, we filtered them by its association with our previously reported set of 9,669 MPC enhancers (Cebola et al., 2015). As expected, a large fraction was associated with MPC enhancers (45.1%/40.9% of downregulated genes and 50%/39.6% of upregulated genes in 1βHet early MPC/late MPC, respectively), and most of these genes were also associated with nearby TEAD1 binding sites (Table S7). Interestingly, almost all genes associated with HNF1B-bound MPC enhancers were also TEAD1 targets (Figure S7B). These included the transcription factor *SOX11* and the transmembrane receptor *ROBO2* (downregulated in 1βHet early MPC and late MPC clusters, Figures 7B–7D and S7C). Notably, *Robo1* and *Robo2* have been recently reported to play a key role during pancreas development by controlling expression of Tead transcription factors and its downstream transcriptional activity, ultimately regulating the expansion of the pancreatic progenitor cell pool (Escot et al., 2018). We thus interrogated the expression of *ROBO1*, *ROBO2*, all four TEAD transcription factors, *YAP1*, *TAZ*, and the well-established TEAD target gene *CTGF*. Indeed, *ROBO2* downregulation in 1βHet early MPCs appears to be compensated for by increased *ROBO1* expression in these cells (Figure 7C). As well, while *TEAD1*, *TEAD2*, *TEAD3*, *YAP1*, and *CTGF* are expressed at lower levels in 1βHet early MPCs, *TEAD4* and *TAZ* appear to be slightly upregulated. Thus, *ROBO2* downregulation in 1βHet early MPCs could affect Hippo signaling in these cells, an adverse effect that appears to be partially compensated for by *ROBO1*, *TEAD4*, and *TAZ* upregulation. Similar *ROBO2*/*ROBO1*, *TEAD*, and *YAP* regulatory events were observed in cells from SOX2^+^ and late MPC clusters (Figure S7D). Notably, the epigenomic loci containing the *ROBO2* and *ROBO1* genes reveals several HNF1B- and TEAD1-bound MPC enhancers at the promoter and upstream regulatory regions of *ROBO2*, with regulatory regions near the *ROBO1* gene located downstream of its promoter (Figure 7D). Additional TEAD1 targets downregulated in 1βHet cells included *SFRP5* in early MPCs and *FZD5* in late MPCs (Figures 7B and S7C, Table S7), known regulators of the non-canonical Wnt and/or Hippo signaling pathways (Rodriguez-Seguel et al., 2013; Sharon et al., 2019), thus potentially accounting for the reduced cell proliferation observed in these cells. To conclude, these analyses reveal that HNF1B haploinsufficiency could result in the defective regulation of effectors of the Wnt and Hippo pathways, which in turn could decrease proliferation of early MPCs.

## Discussion

We describe here the use of a well-controlled hiPSC pancreatic differentiation model to elucidate the molecular mechanisms underlying HNF1B-associated diabetes and pancreatic hypoplasia. Our results reveal that a lack of HNF1B blocks specification of pancreatic fate from FPs. Indeed, upregulated genes in D6-1βHom and D8-1βHom cells are enriched in annotations associated with heart, kidney, and nervous system development, suggesting that absence of HNF1B affects foregut patterning by allowing cells to adopt alternative fates. These results suggest that homozygous loss of HNF1B protein expression in the human embryo is likely to be lethal due to a primary defect in gut tube/foregut formation. Conversely, HNF1B haploinsufficiency allows differentiation of MPCs and ultimately β-like cells. The β-like cells differentiated from 1βHet cells are functional, to the same degree as their 1βWT-derived counterparts, but the former are produced in a smaller amount.

In sharp contrast with a previous study reporting the use of MODY5 patient-derived hiPSCs for differentiation of pancreatic progenitors and β-like cells (Teo et al., 2016), here we detect downregulation of *PDX1* and other important pancreatic regulators, including *PTF1A*, *NKX6.1*, *SOX9*, and *RFX6*, in D13-1βHet cells (MPC stage). The main difference among the studies resides in the strategy used to model HNF1B-associated diabetes. Teo and colleagues used hiPSCs derived from MODY5 patients carrying an S148L mutation that potentially affects the DNA binding efficiency of HNF1B. Thus, binding of mutated HNF1B to its genomic regulatory regions could be less efficient, but not abolished. In this context, an increase in gene expression could be a compensatory mechanism to enhance HNF1B activity. As well, these researchers used non-isogenic hiPSCs derived from another family member and a non-related individual as control cell lines. Thus, the results could be influenced by additional contributions from the genetic background, as discussed in more detail in a recent review (Burgos et al., 2021). On the other hand, for the studies presented here, we used a better controlled cell model in which we completely disrupted *HNF1B* expression from one or both alleles, and used isogenic non-mutated hiPSCs as controls. Our strategy more accurately reflects HNF1B haploinsufficiency, since we see that *HNF1B* expression is reduced by half in D6-1βHet and D13-1βHet cells. Notably, this model might more closely recapitulate the mechanisms underlying HNF1B-associated diabetes in MODY5 patients with nonsense or frameshift mutations, in which the HNF1B protein function is more severely compromised (Edghill et al., 2006a).

One of the earliest transcriptional events we noticed in samples derived from both 1βHom and 1βHet cells is the failure to upregulate *HNF1A-AS1*, whose promoter is bound by HNF1B in WT *in vitro* MPCs. However, while progenitors derived from 1βHom cells have impaired expression of both *HNF1A-AS1* and *HNF1A*, samples derived from their 1βHet counterparts express this lncRNA at considerably lower levels and *HNF1A* close to WT levels. This could be one of the main differences allowing for the early transcriptional divergence among progenitors derived from 1βHom and 1βHet cells, as soon as from the foregut stage (day 6). Interestingly, a very recent work by Ferrer and colleagues shows that *HNF1A-AS1* (renamed as *HASTER* in their work) maintains the expression of *HNF1A* at physiological cell-specific levels through positive and negative feedback loops (Beucher et al., 2021). In the model proposed, increased *HNF1A-AS1* expression (and thus activation of its promoter) downregulates *HNF1A* mRNA expression levels by “sequestering” binding of an *HNF1A* intronic enhancer from the *HNF1A* promoter. Notably, although forced high protein levels of either HNF1A or HNF1B increase *HNF1A-AS1* expression in the EndoC-βH3 cell line, only elevated HNF1A protein levels are able to downregulate the endogenous *HNF1A* mRNA expression (Beucher et al., 2021). This is consistent with our findings because, although low levels of HNF1B in D8-1βHet and D13-1βHet cells result in an important decrease in *HNF1A-AS1* expression, we do not see a concomitant increase in *HNF1A* mRNA levels. It should be noted, however, that embryonic and adult levels of *HNF1A* could be driven by different enhancers. Indeed, while Ferrer and colleagues focus on an enhancer located in an intron of *HNF1A* as the regulatory element “sequestered” by the *HNF1A-AS1* promoter, in our work we detect a TEAD1- and FOXA2-bound regulatory region located upstream of the *HNF1A* promoter. A more detailed study of the interplay between *HNF1A-AS1* and *HNF1A* expression in this model of HNF1B-associated diabetes is an exciting area for future research.

We further report here that HNF1B haploinsufficiency impairs cell proliferation in foregut and MPCs *in vitro*. The quantity of MPCs produced is likely to be influenced by other transcription factors and environmental cues, thereby explaining the variability in the penetrance of HNF1B mutations in human. Importantly, this decrease in MPC number originates from a defect not only in cell proliferation, but also in specification. Indeed, a dose-sensitive effect of HNF1B loss was observed, since heterozygous knockout of HNF1B in hiPSCs resulted in significant impairment, but not complete loss, of pancreatic progenitor cell development. While PDX1^+^ cells can be produced with similar efficiency from 1βWT and 1βHet at day 13 (MPC stage), there is a significant reduction of PDX1^+^/NKX6.1^+^ MPCs. Single-cell transcriptomic analyses reveal that HNF1B haploinsufficiency switches the balance of progenitor cell populations derived *in vitro*. Thus, a low dosage of HNF1B in progenitor cells alters the early stage pancreatic specification program, downregulating the expression of several genes with known or suspected roles in pancreas development. Our analyses suggest that, on one hand, this could be due to impaired early MPC specification from gut progenitor cells. At this time point, 1βHet SOX2^+^ progenitors fail to upregulate *SOX11*, *SOX4*, *GATA6*, and *HMGA2*, among other genes potentially involved in the early specification of pancreatic MPCs. Later on, at the MPC stage, 1βHet early MPCs express lower levels of key pancreatic developmental genes, including *SOX11*, *ROBO2*, and additional TEAD1 target genes whose function could be associated with MPC self-renewal (Cebola et al., 2015; Escot et al., 2018; Willmann et al., 2016; Yu et al., 2019). Interestingly, it has been recently reported in the mouse that *Robo1* and *Robo2* are required to stabilize the pancreatic cell identity after fate induction and, later on, for expansion of the pancreatic progenitor cell pool (Escot et al., 2018). Robo receptors can control the expression of Tead transcription factors and its downstream transcriptional activity. These findings are consistent with our previous report describing a key role for TEAD and YAP in controlling the gene expression program in MPCs (Cebola et al., 2015), and are in agreement with the results presented here. Taken together, these observations allow us to hypothesize that downregulation of some TEAD target genes in 1βHet early MPCs, potentially mediated by impaired *ROBO2* expression, might enhance cell differentiation at the expense of MPC pool self-renewal. We note that *ROBO1* expression could be partially compensating for this effect in early MPCs, thus allowing a plausible explanation for the less efficient, but not truncated, production of β cells from 1βHet hiPSCs.

Other factors that could underlie the adverse effects of HNF1B haploinsufficiency on pancreas development include *SOX4* and *SOX11*. Of these, *SOX11* was robustly downregulated in all cell clusters derived from D13-1βHet samples. *SOX4*, on the other hand, is downregulated in D13-1βHet SOX2^+^ cells. Both factors have been previously described in the context of pancreas development. Sox4 knockout in mice results in *Sox11*, *Neurog3*, and *Neurod1* upregulation in the E12.5 pancreas (Wilson et al., 2005). Sox11 knockout mice present hypoplasia of the pancreas (Sock et al., 2004). Likewise, the reduced *SOX11* expression in D13.1βHet SOX2^+^ progenitors and early MPCs found in our HNF1B-deficient pancreatic cell differentiation model could at least partially explain the organ hypoplasia found in patients with HNF1B-associated diabetes.

Taken together, our findings show that HNF1B haploinsufficiency could result in pancreas hypoplasia in humans due to an altered production of multipotent progenitors. The downstream molecular mechanisms could involve several gene candidates, including *ROBO2*, *SOX4*, and *SOX11*. Downregulation of this set of genes has the potential to alter the early stage pancreatic specification program, which at this time point involves TEAD and YAP gene target regulation through the Hippo signaling pathway. The modulation of these factors during fetal life by environmental stimuli could compensate in part for the decrease in HNF1B expression, explaining the variable penetrance of HNF1B-associated diabetes. Future studies addressing the functional role of these factors could help to develop new therapies against this disease.

## Experimental procedures

### hiPSC generation, characterization, and differentiation

Two hiPSC lines, FSPS13.B and Eipl_1, were used for genome editing and pancreatic differentiation experiments. The hiPSCs were derived from human skin fibroblasts and peripheral blood. Ethics approval was obtained from the National Research Ethics Service Committee East of England, Cambridge East (Ethics Reference 09/h0304/77). Clonal hiPSC mutant lines were generated using the CRISPR-Cas9 technology as described in detail in the supplemental experimental procedures. hiPSCs were cultured and differentiated as previously described (Chia et al., 2019; Cho et al., 2012) with minor modifications as described in the supplemental experimental procedures.

### Western blot, immunofluorescence, FACS, qRT-PCR, apoptosis, and cell proliferation assays

Methods for western blot, immunofluorescence, FACS, qRT-PCR, apoptosis, and cell proliferation assays have been described previously (Chia et al., 2019; Cho et al., 2012; Yiangou et al., 2019), and detailed information is provided in the supplemental experimental procedures. Images were taken using a Zeiss LSM 700 confocal microscope (Carl Zeiss, Jena, Germany).

### RNA-seq

For the bulk RNA-seq experiments, one HNF1B^+/+^, one HNF1B^+/−^, and one HNF1B^−/−^ (targeted WT) clone from the FSPS13.B hiPSC line were differentiated along the pancreatic lineage. RNA was extracted and sequenced as previously described (Chia et al., 2019). Three independent experiments (biological triplicates generated from FSPS13.B clones) were sequenced for each clone at each stage of differentiation. Bioinformatics analyses were carried out following standard procedures (Cebola et al., 2015; Chia et al., 2019; Conesa et al., 2016).

### Single-cell RNA-seq

Single-cell libraries from D13-1βHet and D13-1βWT samples were generated using the Chromium Single Cell 3′ Library & Gel Bead Kit v.2 (PN 120237) from 10× Genomics. Libraries were sequenced on the HiSeq 4000 (Illumina) with 125 bp paired-end sequencing. Analysis of scRNA-seq data included filtering, alignment to the GRCh38 human genome version 28 (Ensembl 92), and unique molecular identifier collapsing performed using the Cell Ranger (v.2.01) pipeline with default mapping arguments (10× Genomics). All further analyses were run with Python 3 using the Scanpy API package (Wolf et al., 2018). Additional details are provided in the supplemental experimental procedures.

### Quantification and statistical analysis

For both FSPS13.B and Eipl_1, we used three WT clones (HNF1B^+/+^; one non-targeted WT and two targeted WT clones), three heterozygous clones (HNF1B^+/−^), and two homozygous clones (HNF1B^−/−^; one with a puromycin cassette in both alleles and one with a puromycin cassette in the first allele and an indel in the second allele). The clone identities are shown in Figures S2C and S2E. The data in the main and supplementary figures are pooled from experiments using FSPS13.B and Eipl_1 clones for qPCR, flow cytometry, and ELISA. Quantification data are presented as the mean ± SEM. Data from clonal lines of the same genotype were combined for calculating the significance of the differences between different genotypes. To directly compare two groups, Student's t test with two-tailed distribution was used to test for statistical significance. p values less than 0.05 were considered statistically significant. All statistical analyses were performed using GraphPad Prism 6.0 (GraphPad Software, San Diego, CA, USA) or the R statistical environment.

### Data and software availability

The accession number for all raw and processed sequencing data reported in this paper is NCBI Gene Expression Omnibus (https://www.ncbi.nlm.nih.gov/geo/): GSE168071.

## Author contributions

R.K., K.T., M.C., S.V., and C.Y.C. performed wet lab experiments. R.K. generated RNA-seq biological samples. P.M. and E.O. performed bulk RNA-seq bioinformatic analyses. E.O., D.M., and S.A.R.-S. analyzed scRNA-seq data. S.A.R.-S. performed epigenomic analyses. R.K. and L.V. conceived the initial experiments. R.K., E.O., L.V., and S.A.R.-S. discussed the results and wrote the manuscript.

## Supplemental information

Document S1. Supplemental experimental procedures, Figures S1–S7, and Tables S1, S2, S5

Table S3. Differentially expressed genes resulting from pairwise comparisons of samples derived from cells with different HNF1B genotypes

Table S4. Gene ontology results. Significant down- and up-regulated gene ontology biological process pathways in 1βWT versus βHet and 1βWT versus 1βHom pairwise comparisons at days 6, 8, 13, 16, and 27 of the differentiation protocol

Table S6. Day13 10× single-cell RNA-seq differentially expressed per genotype in each cluster (p < 0.05)

Table S7. 1βHet differentially regulated genes in early MPCs and late MPCs that are associated with at least one MPC enhancer and/or TEAD1/HNF1B binding sites

Document S2. Article plus supplemental information
