# Supplementary material for: Modeling HNF1B-associated monogenic diabetes using human iPSCs reveals an early stage impairment of the pancreatic developmental program
Source: Stem Cell Reports. 2021 Aug 26;16(9):2289–304. doi: 10.1016/j.stemcr.2021.07.018 (PMC8452540; doi:10.1016/j.stemcr.2021.07.018)
Supplement: Document S1. Supplemental experimental procedures, Figures S1–S7, and Tables S1, S2, S5 [file mmc1.pdf]

Stem Cell Reports, Volume *16*

Supplemental Information

Modeling HNF1B-associated monogenic diabetes using human iPSCs reveals an early stage impairment of the pancreatic developmental program

Ranna El-Khairi, Evelyn Olszanowski, Daniele Muraro, Pedro Madrigal, Katarzyna Tilgner, Mariya Chhatriwala, Sapna Vyas, Crystal Y. Chia, Ludovic Vallier, and Santiago A. Rodríguez-Seguí

Modeling HNF1B-associated monogenic diabetes using human iPSCs reveals an early stage impairment of the pancreatic developmental program

By

Ranna El-Khairi[1,2], Evelyn Olszanowski[3,4], Daniele Muraro[1,2], Pedro Madrigal[1,2], Katarzyna Tilgner[2], Mariya Chhatriwala[1,2], Sapna Vyas[2], Crystal Y. Chia[1,2], Ludovic Vallier[1,2,Φ], Santiago A. Rodríguez-Seguí[3,5,Φ]

SUPPLEMENTARY FIGURE LEGENDS

Figure S1. Characterization of the pancreatic differentiation process by studying the expression of relevant lineage markers at different stages

(A) Separated signal and merged panels for representative immunostaining images presented in **Figure 1C**. Scale bar, 100 μm.

(B) Representative immunostaining of HNF1B and other stage-specific markers, including pluripotency markers (OCT4, NANOG, SOX2), endoderm markers (GATA6, SOX17), foregut progenitor markers (HNF4A, HNF1B), posterior foregut and pancreatic progenitor markers (HNF6, PDX1, NKX6.1, SOX9) and endocrine progenitor (PDX1, NKX6.1, NEUROG3) and hormonal cell markers (CHGA, CPEP, GCG), Scale bar, 100 mm.

(C) Expression of pluripotency markers (*POU5F1*, *NANOG*, *SOX2*), endoderm, mesoderm and neuroectoderm markers (*SOX17*, *CXCR4*, *GATA6*, *Brachyury/T*, *PAX6*), foregut progenitor markers (*HNF1B*, *HNF4A*, *FOXA2*), posterior foregut markers (*HNF1B*, *FOXA2*, *HNF4A*, *PDX1*, *SOX9*, *ONECUT1*), pancreatic progenitor markers (*PDX1*, *SOX9*, *NKX6-1*), endocrine progenitor markers (*NEUROG3*, *NEUROD1*, *PDX1*, *NKX6-1*) and hormonal cell markers (*PDX1*, *NKX6-1*, *INS*, *GCG*, *SST*, *PAX6*) during the differentiation of hiPSCs into hormonal cells. mRNA levels were measured by qRT-PCR (n=5 independent experiments at each stage of differentiation using the FSPS13.B wild-type clone) and normalised to the housekeeping gene porphobilinogen deaminase (*PBGD*). Data are presented as mean ± SEM unless otherwise indicated.

Figure S2. Derivation and characterisation of HNF1B mutant hiPSC lines. Early stage differentiation

(A) Schematic showing the human *HNF1B* genomic locus, indicating the protein domains encoded within the *HNF1B* exons.

(B) Genotyping of WT HNF1B+/+, heterozygous HNF1B+/- and homozygous HNF1B-/- mutant hiPSCs. The corresponding sequences of a representative heterozygous mutant line (2nd allele) are shown underneath the WT reference sequence.

(C) mRNA expression of the pluripotency markers *NANOG* and *OCT4* for undifferentiated HNF1B+/+ (D0-1βWT), HNF1B+/- (D0-1βHet) and HNF1B-/- (D0-1βHom) clones for the FSPS13.B and Eipl_1 hiPSC lines (n=3 independent experiments for each clone). The mRNA levels were measured by qRT-PCR and normalized to the house-keeping gene *PBGD*.

(D) Western blot showing expression of HNF1B protein at ~65kb against housekeeping control B-actin (42kb) at the foregut progenitor stage (Day 6) for HNF1B+/+ (D6-1βWT), HNF1B+/- (D6-1βHet) and HNF1B-/- (D6-1βHom) clones for the FSPS13.B and Eipl_1 hiPSC lines.

(E) mRNA expression of *HNF1B* at the foregut progenitor stage (Day 6) for HNF1B+/+ (D6-1βWT), HNF1B+/- (D6-1βHet) and HNF1B-/- (D6-1βHom) clones for the FSPS13.B and Eipl_1 hiPSC lines (n=3 independent experiments). mRNA levels were measured by qRT-PCR and normalised to the house-keeping gene *PBGD*.

(F) Expression of *SOX17* and *CXCR4* in DE cells derived from D6-1βWT, D6-1βHet and D6-1βHom cells. The mRNA levels were measured by qRT-PCR and normalized to the house-keeping gene *PBGD*. Data pooled from n=5 independent experiments for each of the 8 FSPS13.B clones, and n=3 independent experiments for each of the 8 Eipl_1 clones, clone identities as per panel (C).

(G) FACS analysis of cells stained for the DE marker SOX17. There was no significant difference in the number of cells staining for SOX17 in DE cells derived from D6-1βWT, D6-1βHet and D6-1βHom cells. Data pooled from n=5 independent experiments for each of the 8 FSPS13.B clones,

and n=3 independent experiments for each of the 8 Eipl_1 clones, clone identities as per panel (C). Student's t test with two-tailed distribution was used for statistical analysis. All data are presented as mean ± SEM unless otherwise indicated. P-values were not significant.

Figure S3. Differentiation of HNF1B+/+ and HNF1B+/- and HNF1B-/- hiPSCs produces endocrine progenitor (EP) cells (Day 16)

(A) Expression of *NEUROD1*, *NEUROG3* and *GLIS3* in pancreatic progenitor cells derived from HNF1B+/+ (D16-1βWT) and HNF1B+/- (D16-1βHet) and HNF1B-/- (D16-1βHom) hiPSC lines. mRNA levels were measured by qRT-PCR and normalized to the house-keeping gene *PBGD*. Data pooled from n=5 independent experiments for each of the 8 FSPS13.B clones, with identities as per **Figure S2C**. Student's t test with two-tailed distribution was used for statistical analysis. All data are presented as mean ± SEM unless otherwise indicated. $*p < 0.05$; $**p < 0.01$; $***p < 0.001$ and $****p < 0.0001$.

(B) Percentage of cells expressing PDX1, NKX6.1 and NEUROD1 and representative FACS dot plots of cells stained for PDX1 and NEUROD1. The percentage of each cell population is indicated in the corresponding quadrant for all FACS plots. Replicates and statistics as indicated in panel (A).

Figure S4. Differential expression of key pancreatic differentiation markers between HNF1B+/+ (1βWT) and HNF1B+/- (1βHet) and HNF1B-/- (1βHom) cells as quantified from RNA-seq data.

(A) Gene expression for key foregut and pancreatic markers as detected by RNA-seq (n = 3 at each stage of differentiation and for each genotype). Counts were normalized using the fragments per kilobase of transcript per million mapped reads (fpkm) function of the DESeq2 package. Data are presented as mean ± SEM unless otherwise indicated.

(B) Venn diagrams showing the overlap of downregulated and upregulated genes in D13-1βHom and D13-1βHet, when compared to D13-1βWT cells. Selected genes cording for transcription factors are listed next to the Venn diagrams.

Figure S5. Gene expression variation in stem, progenitor and endocrine cells derived in vitro from HNF1B+/+ and HNF1B+/- and HNF1B-/- hiPSCs

(A) Principal component analysis of expressed genes (counts >1) in HNF1B+/+ (1βWT), HNF1B+/- (1βHet) and HNF1B-/- (1βHom) cells. n= 3 independent experiments per sample.

(B) Heatmap of sample-to-sample distances using log-transformed values. Rectangles correspond to measurements from individual biological replicates.

Figure S6. scRNA-seq analysis reveals cell populations derived in vitro from 1βWT and 1βHet iPSCs

(A) Violin plots showing the expression for selected markers in human in vitro derived pancreatic cells clustered as in **Figure 6A**. Cluster PROCR+ express EMT markers.

(B) Feature and violin plots showing the expression for selected EMT and PROCR+ markers (Wang et al. 2020) in human in vitro derived pancreatic cells clustered as in **Figure 6A**.

(C) Total number of cells per cluster and differentiation stage or genotype.

Figure S7. HNF1B haploinsufficiency impairs the early stage pancreatic developmental program by altering expression of key non-canonical Wnt and Hippo signalling pathway components

(A) Dot plot showing expression of the top markers significantly up- and downregulated in 1βHet samples for the progenitor cell clusters as presented in **Figure 6A**. Color intensity indicates mean expression (normalized) in a cluster, dot size indicates the proportion of cells in a cluster expressing the gene.

(B) Percent of genes up or downregulated in 1βHet early MPC and late MPC which are associated with at least 1 MPC enhancer and/or TEAD1/HNF1B binding sites. MPC enhancers taken from Cebola et al, Nat Cell Biol 17, 615-626.

(C) UCSC genome browser snapshots of the *SFRP5* and *SOX11* genomic loci. ChIP-seq was used to locate binding sites of HNF1B, ONECUT1, FOXA2 and TEAD1 in MPCs (data from Cebola et al. 2015). ChIP-seq for H3K4me1 and H3K27ac histone modifications denotes the epigenomic printing of active enhancers. MPC enhancers enriched in HNF1B signal in this locus are highlighted in yellow.

(D) Dot plot showing expression of *ROBO1*, *ROBO2*, selected Hippo pathway components and its known target *CTGF*. Color intensity indicates mean expression (normalized) in a cluster, dot size indicates the proportion of cells in a cluster expressing the gene.

SUPPLEMENTARY TABLES

Table S1. Summary of genotypes for the targeted clones for FSPS13.B (top) and Eipl_1 (bottom) hiPSC lines. The number of clones with no integration of the puromycin resistance cassette (HNF1B WT clones) or integration of the puromycin resistance cassette in one or two alleles (HNF1B homozygous knockout) of the HNF1B gene is shown. For clones where there is integration of the puromycin resistance cassette in one allele, the 2nd allele was either WT (HNF1B heterozygous knockout) or contained an in-frame or frameshift mutation (HNF1B homozygous knockout).

Table S2. Alignment details for raw bulk RNA-seq and ChIP-seq data used in this study.

Table S3. Differentially expressed genes resulting from pairwise comparisons of samples derived from cells with different HNF1B genotypes.

Table S4. Gene ontology results. Significant down- and up-regulated gene ontology biological process (GO - BP) pathways in 1βWT vs βHet and 1βWT vs 1βHom pairwise comparisons at days 6, 8, 13, 16 and 27 of the differentiation protocol.

Table S5. Day13 10x single-cell RNA-seq initial clustering. Top 50 cell cluster markers.

Table S6. Day13 10x single-cell RNA-seq differentially expressed per genotype in each cluster ($p<0.05$).

Table S7. 1βHet differentially regulated genes in early MPC and late MPC that are associated with at least 1 MPC enhancer and/or TEAD1/HNF1B binding sites.

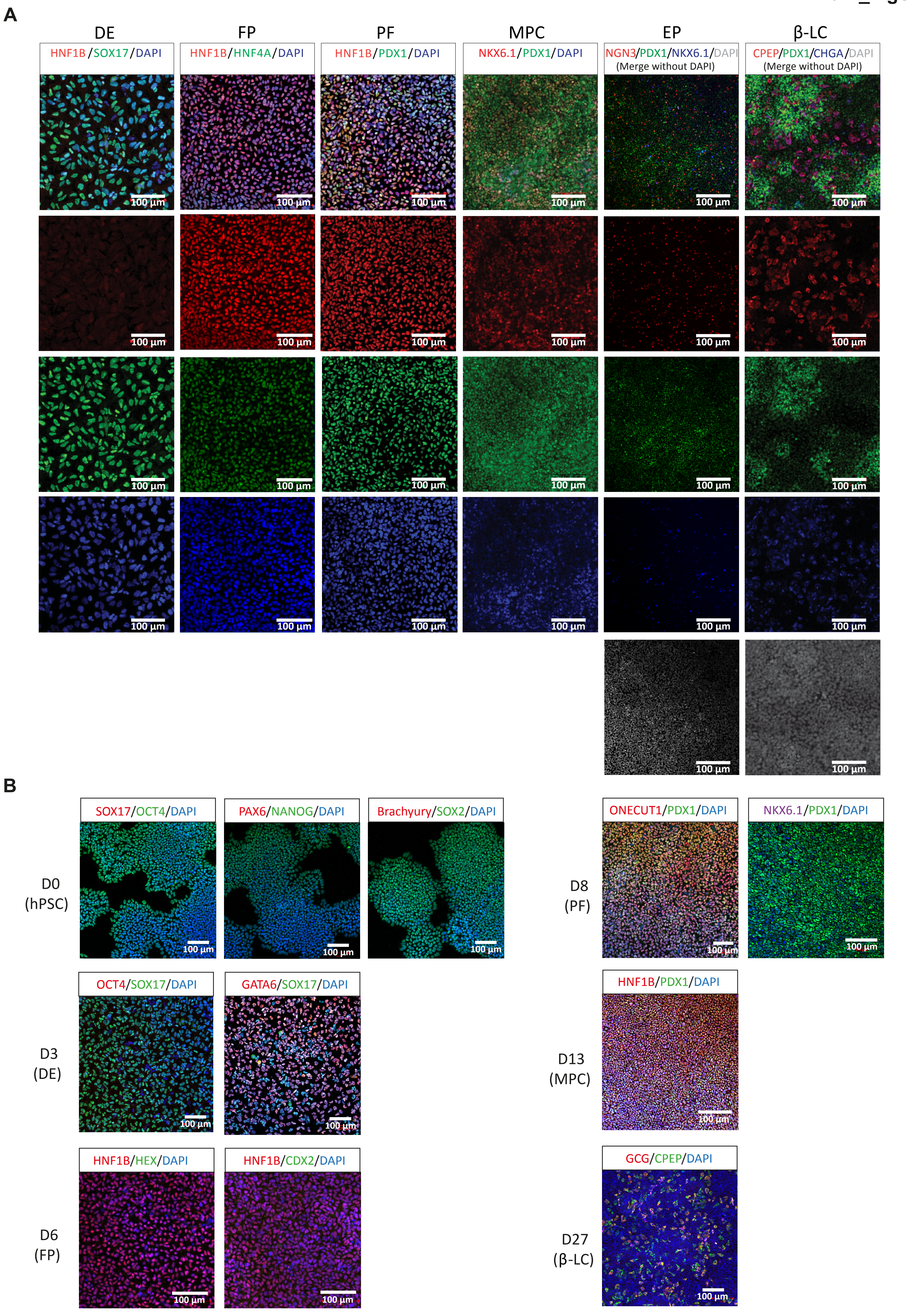
A
DE
FP
PF
MPC
EP
β-LC
HNF1B/SOX17/DAPI
HNF1B/HNF4A/DAPI
HNF1B/PDX1/DAPI
NKX6.1/PDX1/DAPI
NGN3/PDX1/NKX6.1/DAPI
(Merge without DAPI)
CPEP/PDX1/CHGA/DAPI
(Merge without DAPI)
100 µm
B
D0
(hPSC)
SOX17/OCT4/DAPI
PAX6/NANOG/DAPI
Brachyury/SOX2/DAPI
D3
(DE)
OCT4/SOX17/DAPI
GATA6/SOX17/DAPI
D6
(FP)
HNF1B/HEX/DAPI
HNF1B/CDX2/DAPI
D8
(PF)
ONECUT1/PDX1/DAPI
NKX6.1/PDX1/DAPI
D13
(MPC)
HNF1B/PDX1/DAPI
D27
(β-LC)
GCG/CPEP/DAPI

Khairi_FigS1 (cont)

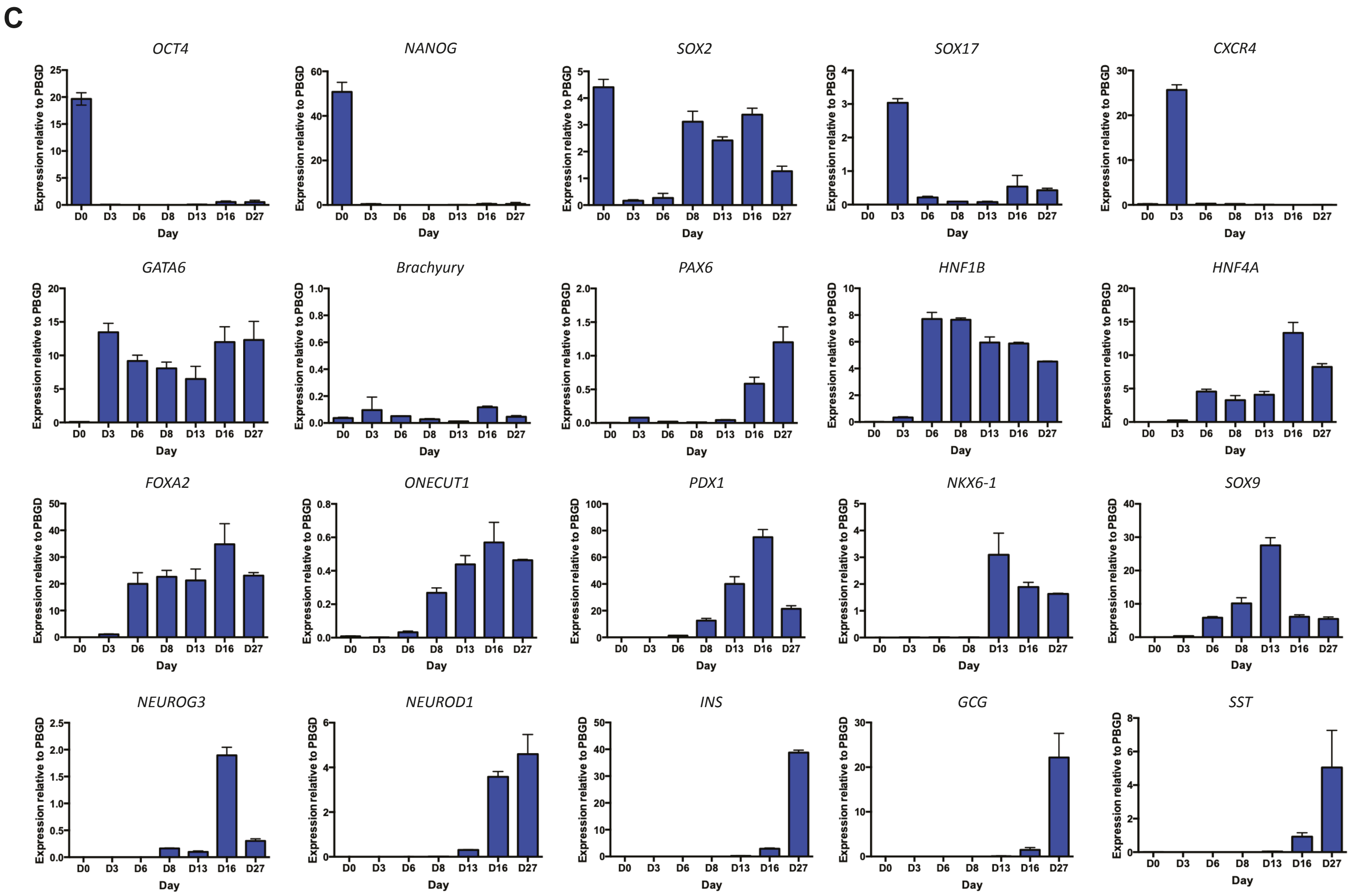

Khairi_FigS2

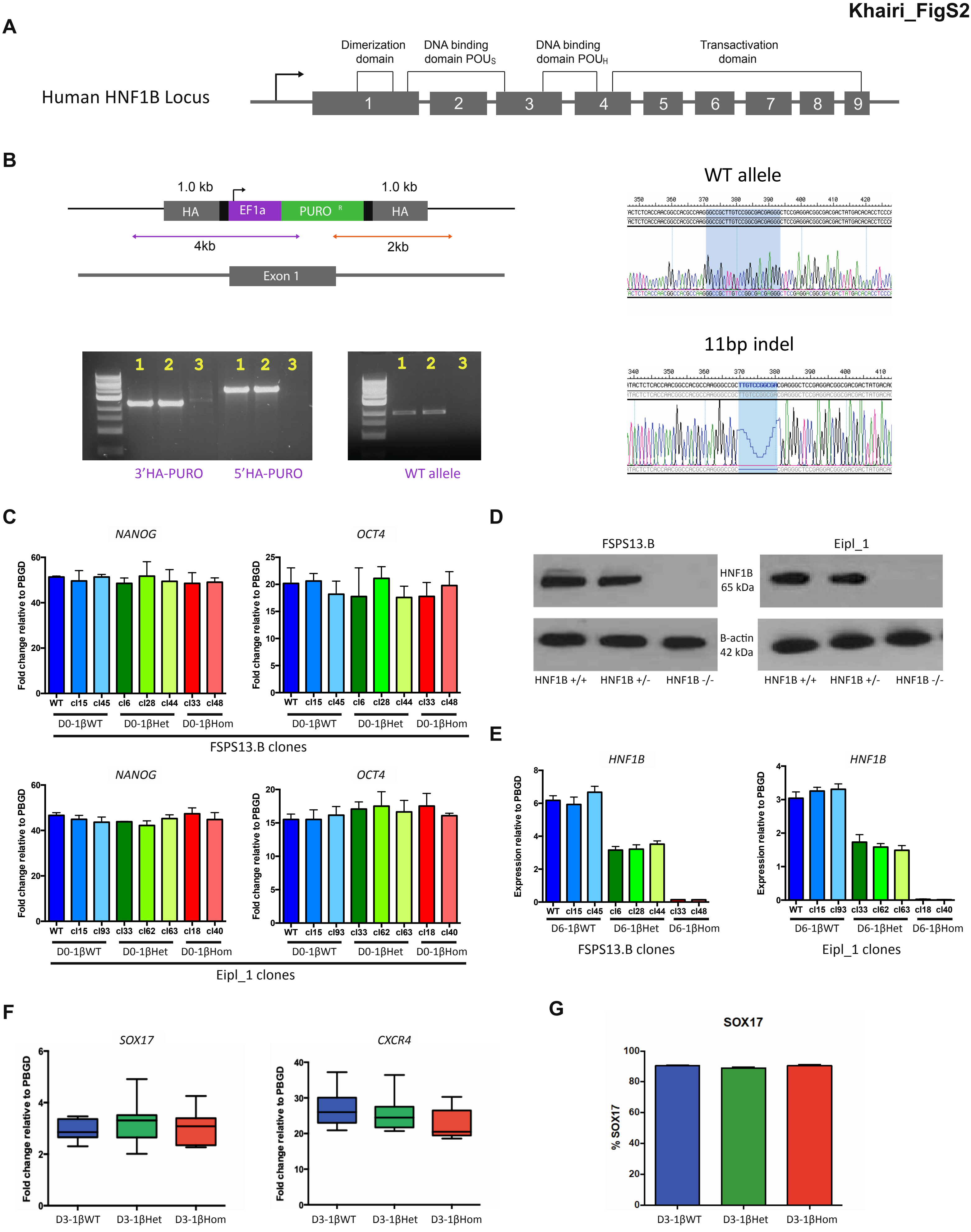

Khairi_FigS3

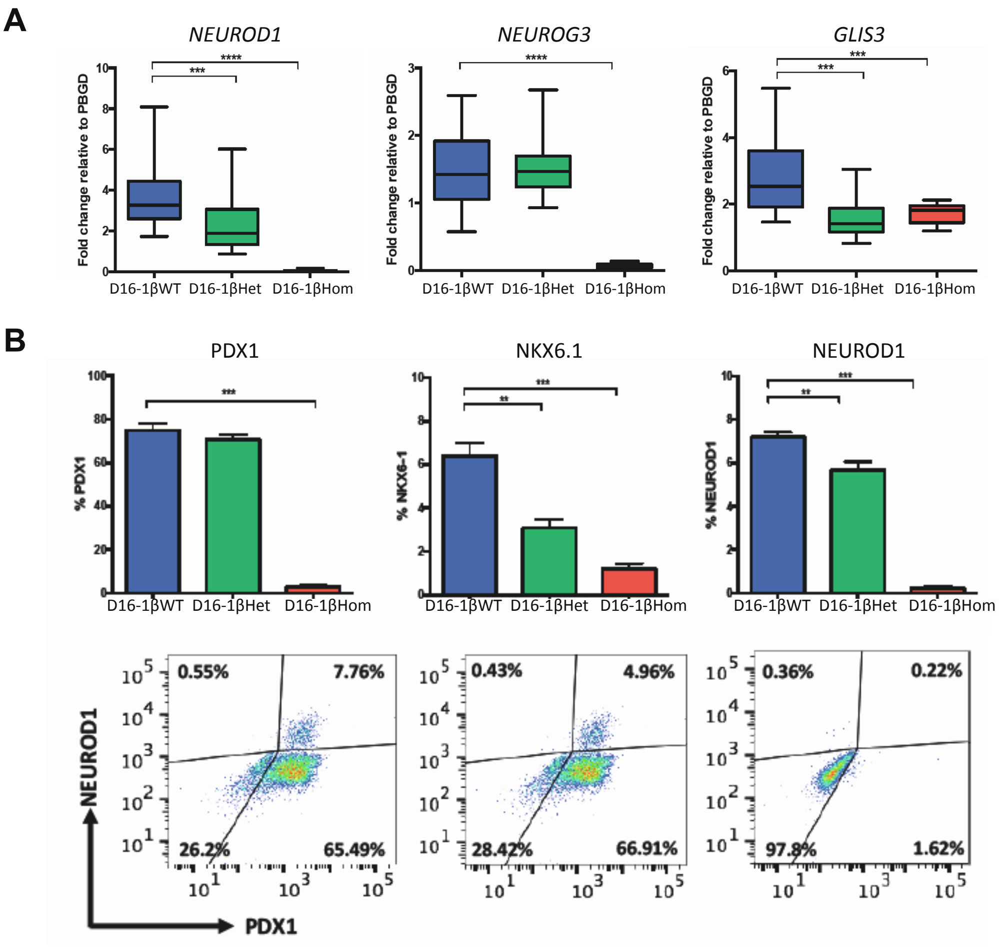

Khairi_FigS4

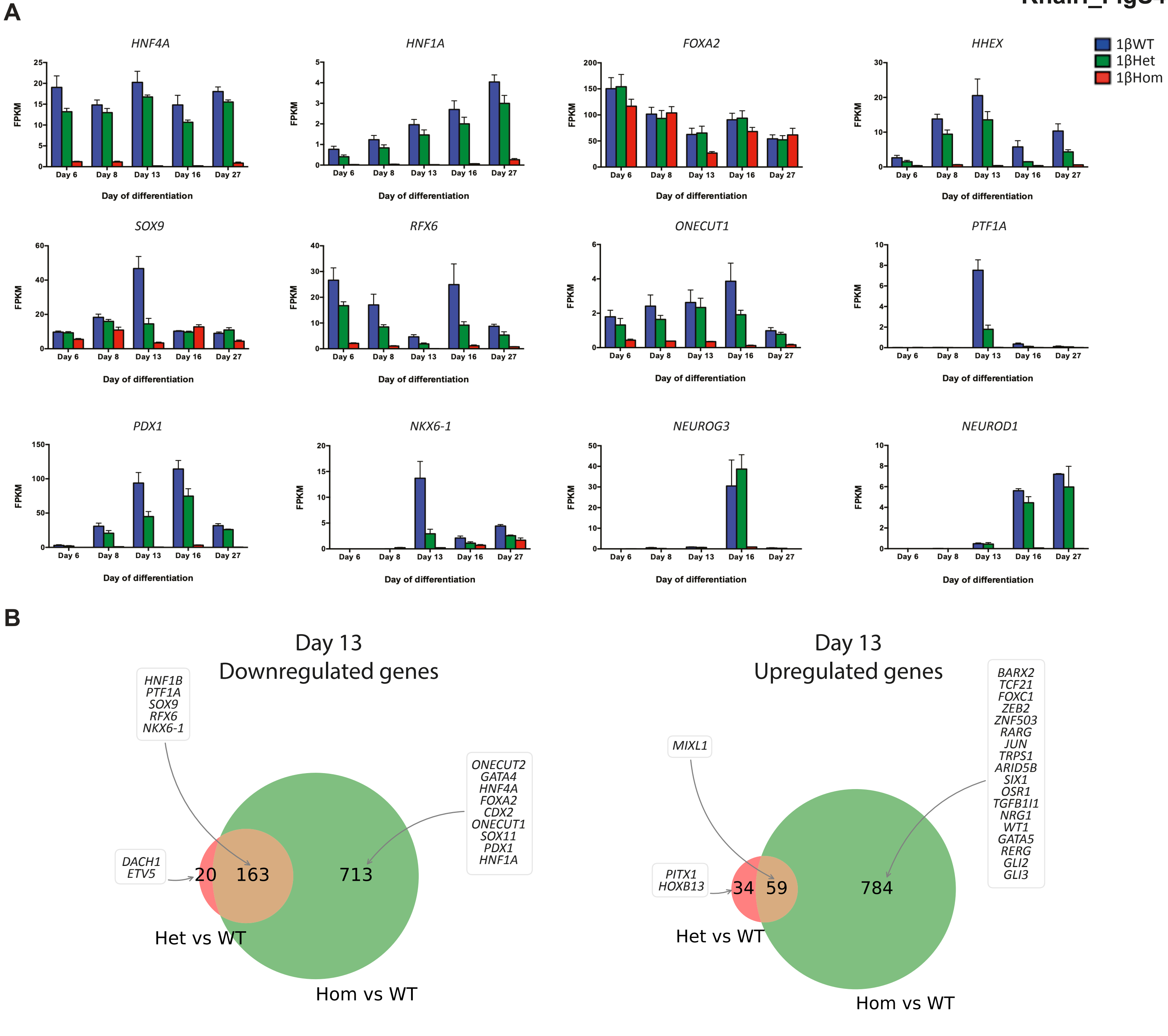

A

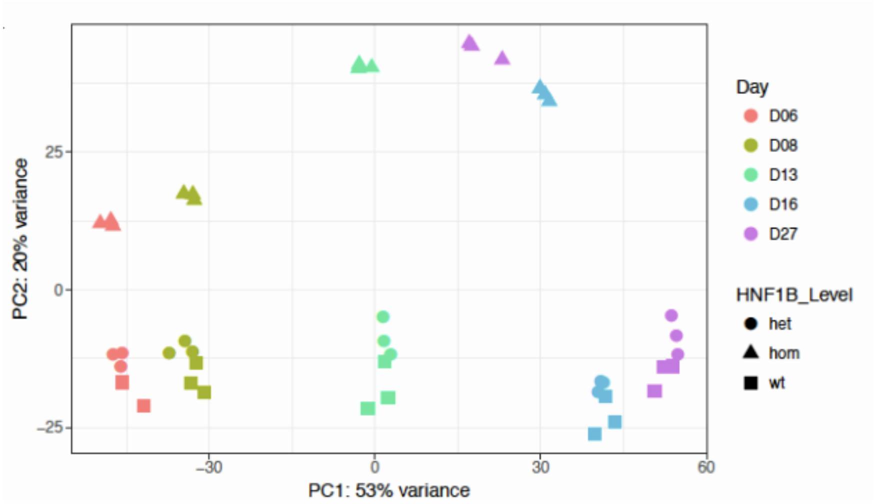
Day
D06
D08
D13
D16
D27
HNF1B_Level
het
hom
wt
PC2: 20% variance
PC1: 53% variance
25
0
−25
−30
0
30
60

B

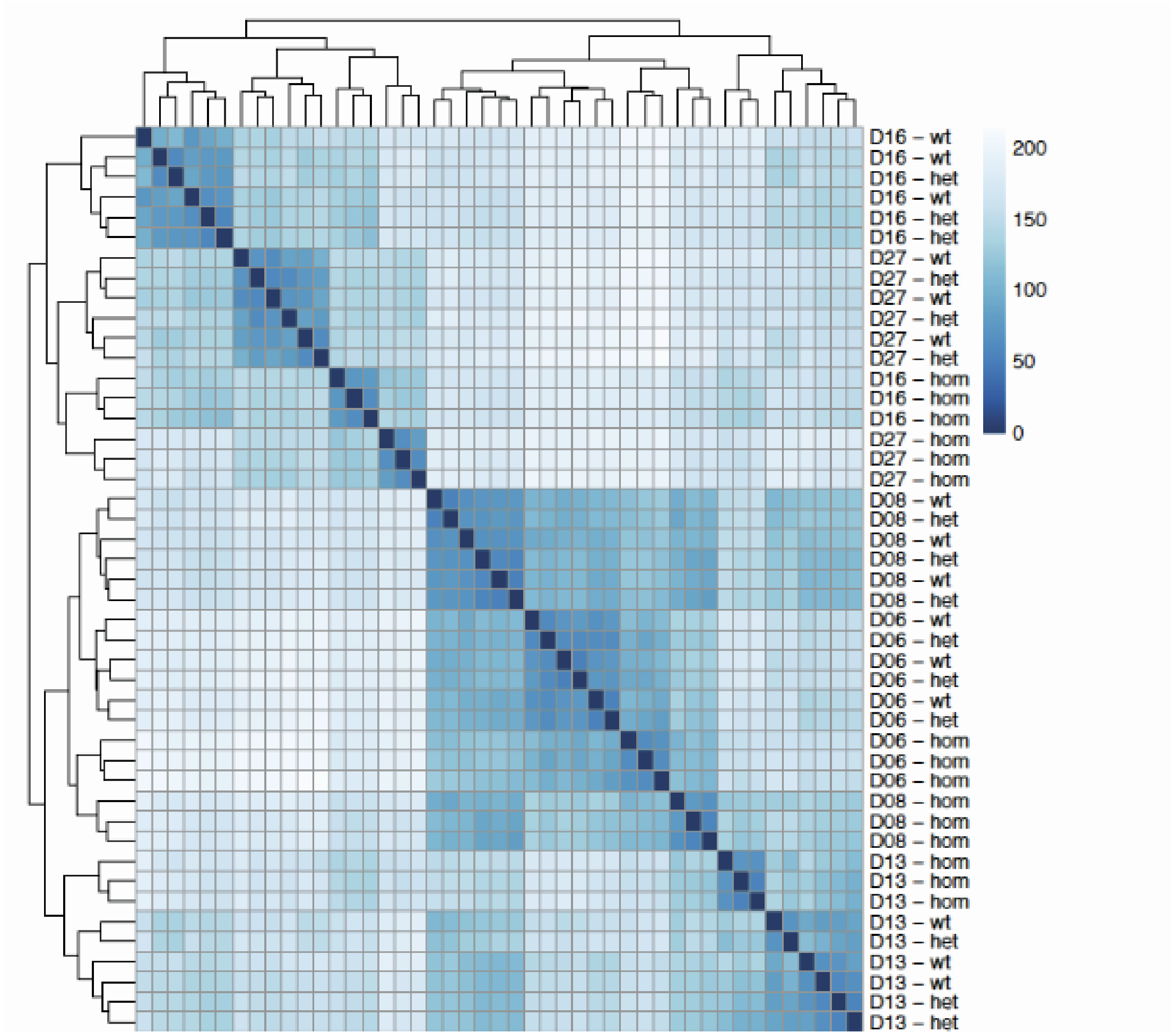
D16 – wt
D16 – wt
D16 – het
D16 – wt
D16 – het
D16 – het
D27 – wt
D27 – het
D27 – wt
D27 – het
D27 – wt
D27 – het
D16 – hom
D16 – hom
D16 – hom
D27 – hom
D27 – hom
D27 – hom
D08 – wt
D08 – het
D08 – wt
D08 – het
D08 – wt
D08 – het
D06 – wt
D06 – het
D06 – wt
D06 – het
D06 – wt
D06 – het
D06 – hom
D06 – hom
D06 – hom
D08 – hom
D08 – hom
D08 – hom
D13 – hom
D13 – hom
D13 – hom
D13 – wt
D13 – het
D13 – wt
D13 – wt
D13 – het
D13 – het
200
150
100
50
0

Khairi_FigS6

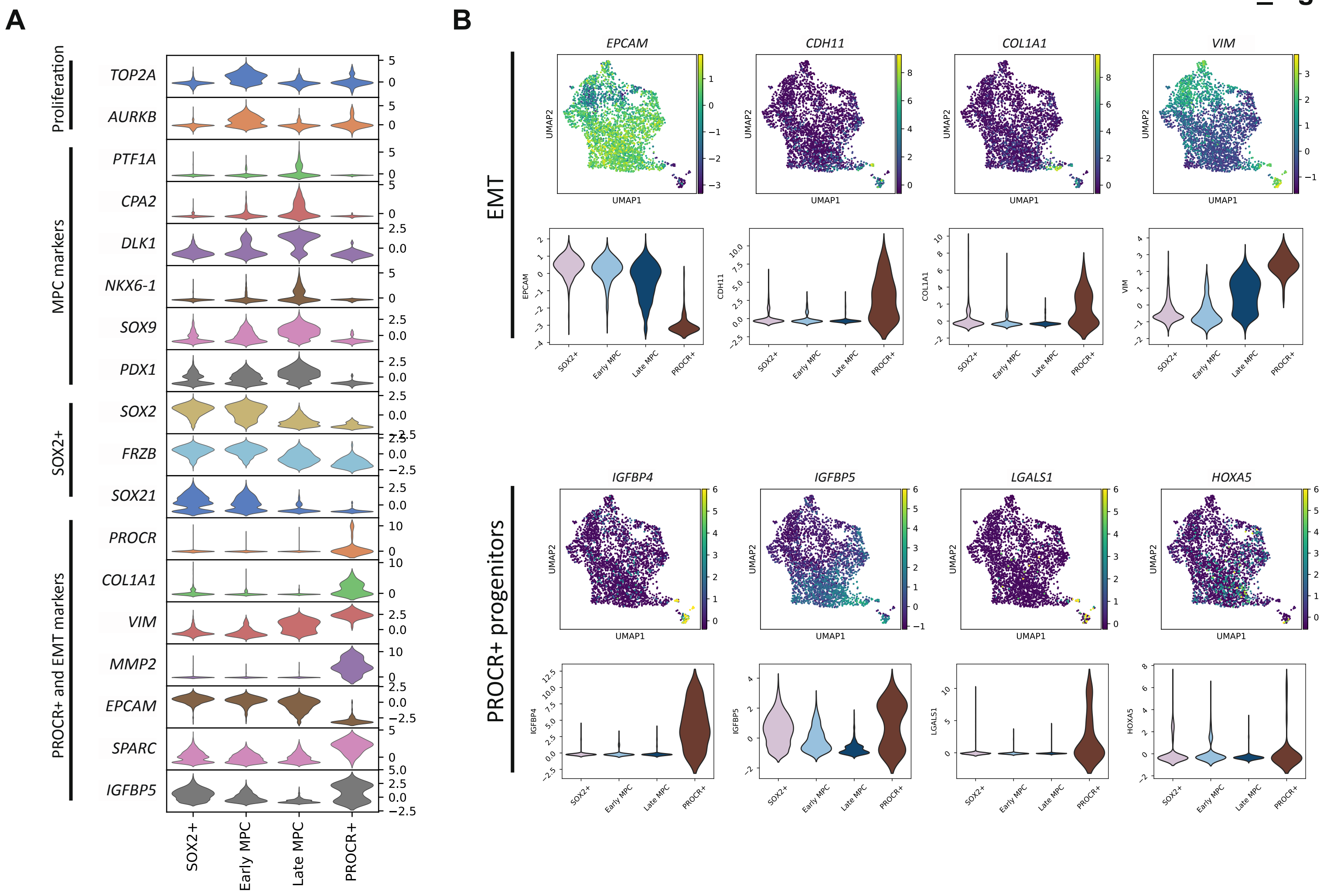

C

Genotype	Cluster # SOX2+	Early MPC	Late MPC	PROCR+	Total # cells/genotype
**1βHet**	765	302	677	25	1769
**1βWT**	653	317	425	52	1447

Khairi_FigS7

A

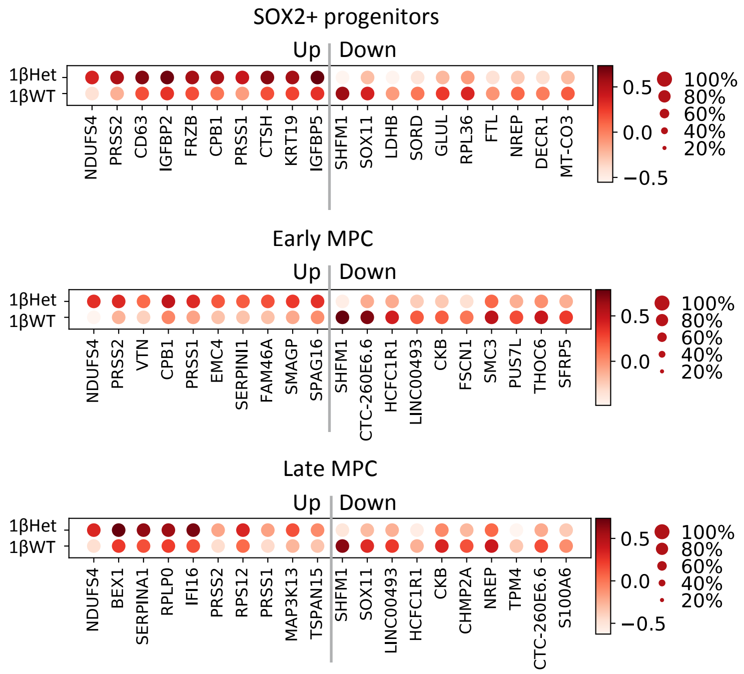

C

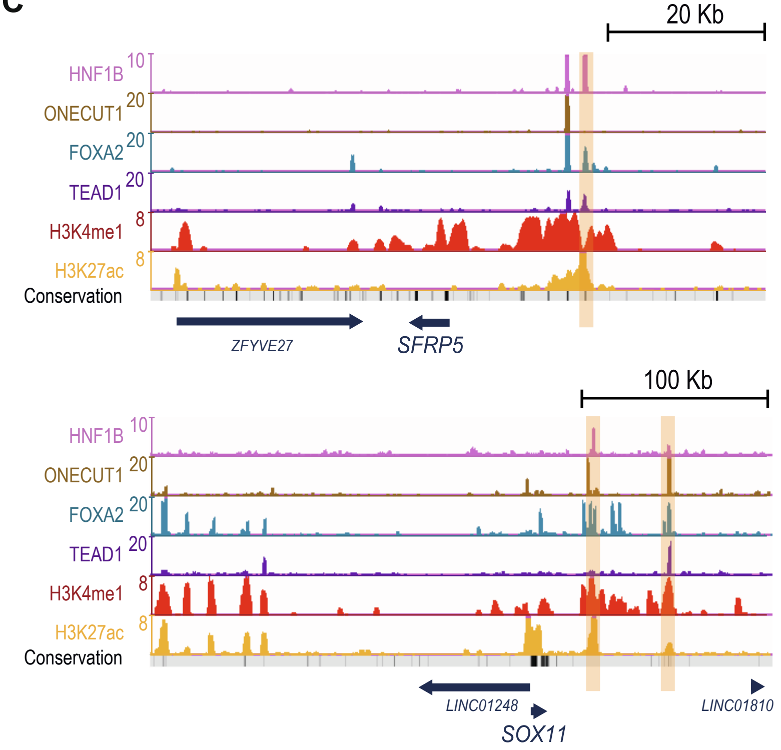

B

	Percent of genes up or downregulated in 1βHet early MPC and late MPC which are associated with at least one:			
	MPC enhancer	MPC enhancer + TEAD1	MPC enhancer + HNF1B	MPC enhancer + HNF1B + TEAD1
% of Markers **up** in **1βHet Early MPC**	50.0	47.1	29.4	29.4
% of Markers **down** in **1βHet Early MPC**	45.1	36.6	22.0	20.7
% of Markers **up** in **1βHet Late MPC**	39.6	33.6	16.8	14.8
% of Markers **down** in **1βHet Late MPC**	40.9	36.2	21.7	20.9

D

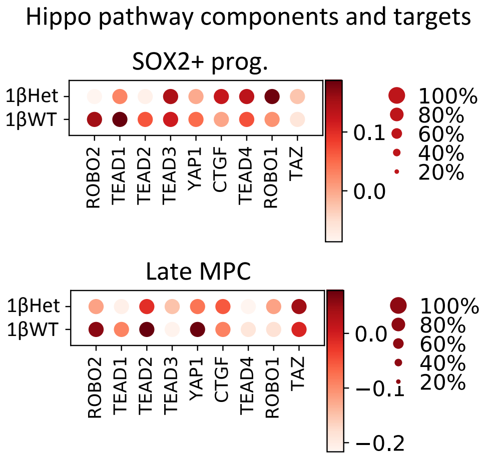

Table S1. Summary of genotypes for the targeted clones for FSPS13.B (top) and Eipl_1 (bottom) hiPSC lines. The number of clones with no integration of the puromycin resistance cassette (HNF1B WT clones) or integration of the puromycin resistance cassette in one or two alleles (HNF1B homozygous knockout) of the HNF1B gene is shown. For clones where there is integration of the puromycin resistance cassette in one allele, the 2nd allele was either WT (HNF1B heterozygous knockout) or contained an in-frame or frameshift mutation (HNF1B homozygous knockout).

**FSPS13.B**	**1 st allele**	**2 nd allele**	**Number of targeted clones**
**WT**	WT	WT	4
**Heterozygous**	Puro R cassette	WT	3
**Homozygous**	Puro R cassette	Puro R cassette	1
	Puro R cassette	NHEJ (frameshift mutation)	14 (4)

**Eipl_1**	**1 st allele**	**2 nd allele**	**Number of targeted clones**
**WT**	WT	WT	8
**Heterozygous**	Puro R cassette	WT	5
**Homozygous**	Puro R cassette	Puro R cassette	1
	Puro R cassette	NHEJ (frameshift mutation)	29 (10)

NHEJ: non-homologous end joining.

Table S2. Alignment details for raw bulk RNA-seq and ChIP-seq data used in this study.

Experiment	Sample name	Day	HNF1B genotype / Tissue	Library size	Mapped reads	Source
bulk RNA-seq in in vitro derived pancreatic progenitor and endocrine cells	13B Cl.45 D6_1	6	WT	118384519	88543640	This study
	13B Cl.6 D6_1	6	Het	116131701	86113194	This study
	13B Cl.48 D6_1	6	Hom	111558545	85740614	This study
	13B Cl.45 D6_2	6	WT	111655388	84139726	This study
	13B Cl.6 D6_2	6	Het	129777107	98644498	This study
	13B Cl.48 D6_2	6	Hom	111826972	88110998	This study
	13B Cl.45 D6_3	6	WT	139606811	103703665	This study
	13B Cl.6 D6_3	6	Het	106679668	80614687	This study
	13B Cl.48 D6_3	6	Hom	128714446	98328972	This study
	13B Cl.45 D8_1	8	WT	123888095	93134642	This study
	13B Cl.6 D8_1	8	Het	147013538	112050531	This study
	13B Cl.48 D8_1	8	Hom	123679567	93162048	This study
	13B Cl.45 D8_2	8	WT	127493326	98438445	This study
	13B Cl.6 D8_2	8	Het	127798804	95737326	This study
	13B Cl.48 D8_2	8	Hom	122936103	92031674	This study
	13B Cl.45 D8_3	8	WT	117085741	86900383	This study
	13B Cl.6 D8_3	8	Het	119824087	90038548	This study
	13B Cl.48 D8_3	8	Hom	119466266	90660554	This study
	13B Cl.45 D13_1	13	WT	142169329	102528677	This study
	13B Cl.6 D13_1	13	Het	135094009	93279477	This study
	13B Cl.48 D13_1	13	Hom	112511990	85968763	This study
	13B Cl.45 D13_2	13	WT	118693241	83580395	This study
	13B Cl.6 D13_2	13	Het	132360151	94662742	This study
	13B Cl.48 D13_2	13	Hom	140083454	103631461	This study
	13B Cl.45 D13_3	13	WT	127000896	96077132	This study
	13B Cl.6 D13_3	13	Het	117594841	84714110	This study
	13B Cl.48 D13_3	13	Hom	122503320	92967061	This study
	13B Cl.45 D16_1	16	WT	117505611	81310336	This study
	13B Cl.6 D16_1	16	Het	130164511	91700486	This study
	13B Cl.48 D16_1	16	Hom	141466373	100263082	This study
	13B Cl.45 D16_2	16	WT	147170171	96305978	This study
	13B Cl.6 D16_2	16	Het	147452466	87898580	This study
	13B Cl.48 D16_2	16	Hom	133070182	90936195	This study
	13B Cl.45 D16_3	16	WT	113804780	84558461	This study
	13B Cl.6 D16_3	16	Het	134215663	93391591	This study
	13B Cl.48 D16_3	16	Hom	120543917	81094469	This study
	13B Cl.45 D27_1	27	WT	125249986	90666868	This study
	13B Cl.6 D27_1	27	Het	116555579	87162835	This study
	13B Cl.48 D27_1	27	Hom	116216729	85354846	This study
	13B Cl.45 D27_2	27	WT	143787152	94199999	This study
	13B Cl.6 D27_2	27	Het	118236331	82906196	This study
	13B Cl.48 D27_2	27	Hom	127138325	80287366	This study
	13B Cl.45 D27_3	27	WT	133940466	92711641	This study
	13B Cl.6 D27_3	27	Het	130352438	93655003	This study
	13B Cl.48 D27_3	27	Hom	137048178	95732442	This study
ChIP-seq	FOXA2	NA	*In vitro* MPCs	25760337	21579594	E-MTAB-1990 *
	ONECUT1	NA	*In vitro* MPCs	25606334	20745554	E-MTAB-1990 *
	HNF1B	NA	*In vitro* MPCs	25645350	19915667	E-MTAB-1990 *
	TEAD1	NA	*In vitro* MPCs	25813944	20389873	E-MTAB-3061 *
	H3K27ac	NA	*In vitro* MPCs	22219409	17739290	E-MTAB-3061 *
	H3K4me1	NA	*In vitro* MPCs	51072767	44298878	E-MTAB-1990 *
	INPUT	NA	*In vitro* MPCs	49562149	37147369	E-MTAB-1990 *

* data from ArrayExpress Archive

Table S5. Day13 10x single-cell RNA-seq initial clustering. Top 50 cell cluster markers

Marker #	Cluster			
	SOX2+	Early MPC	Late MPC	PROCR+
1	RP11-834C11.4	NDFIP1	HMGB2	TFPI
2	SEMA3C	MEST	H2AFZ	VIM
3	PTPN13	FTL	SMC4	COL3A1
4	SOX2	DLK1	CENPU	TMSB10
5	DSP	SERPINB6	CKS1B	MAP1B
6	IGFBP5	CLU	CDK1	TPM4
7	IGFBP2	AMBP	KIAA0101	RHOC
8	TMSB4X	DUSP5	ZWINT	SFRP1
9	TAGLN2	LAPTM4B	TUBA1B	HEY1
10	ANXA3	PHGDH	NUSAP1	PFN1
11	PERP	SOX9	UBE2C	PHLDA1
12	KLF5	VIM	MAD2L1	PTMS
13	NR2F1	PABPC1	HMGN2	TMSB4X
14	PAM	HMGCS2	HMGB1	COL1A2
15	PPDPF	TTYH1	ASPM	PTN
16	MYL12B	ALDH1A1	BRCA2	GNG11
17	PLEKHA5	RAMP1	CENPF	ETS1
18	CD63	LDHB	DEK	ACTG1
19	ANXA2	ZFP36L2	PRC1	NES
20	NPW	GATM	TUBB	SPARC
21	IGDCC3	KIRREL2	CENPW	MAGED2
22	CLDN4	COX7C	CBX5	MMP2
23	PDLIM1	BTG1	TUBB4B	IFITM3
24	UCP2	SCD	NUCKS1	FAM212A
25	GSTP1	RAB3B	SGOL1	BGN
26	TPM1	PKDCC	TOP2A	CFL1
27	FOXP1	BEX1	MKI67	BASP1
28	EPCAM	CPA2	SMC2	IGFBP4
29	KRT19	SERINC5	TPX2	IFITM2
30	PGM2L1	RPLP1	RAD51AP1	TLN1
31	CLDN6	NQO2	KIF11	ARPC2
32	CTSH	APOE	TK1	SEPT11
33	RUNX1	SLC4A4	TYMS	SPRY1
34	SPINT1	TM4SF4	C21orf58	COL4A1
35	UNC13C	ECE1	RRM2	TUBA1A
36	TNNC1	PDX1	PTMA	ATP5E
37	WFDC2	LIN28A	BIRC5	THY1
38	DSTN	CAMK2N1	KIF15	HSPB1
39	PRSS8	TCF7L2	ORC6	39326
40	SPINT2	ID2	ATAD5	MEF2C
41	ELF1	PLK2	MIS18BP1	ITGA1
42	FRZB	SMC3	CENPK	FILIP1
43	CD9	JAG1	KIF20B	ACTB
44	STARD10	LAMA1	PBK	PLAT
45	HOTAIRM1	ATP5A1	CLSPN	MLLT11
46	EPSTI1	PRTG	TACC3	SERPINH1
47	CELF2	HEY1	DIAPH3	NFIA
48	C2orf54	FN1	TMPO	ANXA6
49	SOX21	FLRT3	NUF2	TPM2
50	EZR	RPLP0	DLGAP5	SNCA

EXPERIMENTAL PROCEDURES

hiPSC generation and characterization

Two hiPSC lines, FSPS13.B and Eipl_1 were used for genome editing and pancreatic differentiation experiments. The hiPSCs were derived from human skin fibroblasts and peripheral blood (http://www.hipsci.org/lines/#/lines/HPSI0813i-fpdm_2, http://www.hipsci.org/lines/#/lines/hpsi0114i-eipl_1). Ethics approval was obtained from the National Research Ethics Service (NRES) Committee East of England, Cambridge East (Ethics reference no. 09/h0304/77).

hiPSC culture

Undifferentiated hiPSCs were routinely cultured under feeder-free conditions on vitronectin-coated (STEMCELL Technologies #07180) tissue culture plates (Corning) with Essential 8 Medium (Life Technologies #A1517001). The medium was changed every day, and cells were passaged every 4-5 days using 0.5 mM EDTA (Life Technologies, #15575-020) to dissociate cells. In all hiPSC cultures, 10 μM Rho-associated protein kinase (ROCK) inhibitor, Y-27632 (Selleck Chemicals, #S1049), was only added into the culture media when thawing hiPSCs. Human iPSCs were maintained at 37°C with 5% CO_2 and regularly tested negative for mycoplasma contamination and for chromosomal aberrations.

Cell preparation for pancreatic differentiation

Human iPSCs were passaged and seeded onto 12-well plates using E8 supplemented with Y27632 (Rho-associated, coiled-coil containing protein kinase, ROCK Inhibitor; 10μM). Cells were plated as single cells and the plating density was optimised for each hiPSC line (approximately 80,000 to 100,000 cells per well of a 12-well plate). After 24 hours, the media was replaced with fresh E8 media without ROCK Inhibitor.

Pancreatic differentiation protocol

To induce definitive-endoderm differentiation (days 1-3), cells were cultured in CDM-PVA supplemented with Activin (100ng/ml), FGF2 (80ng/ml), BMP4 (10ng/ml), Ly294002 (10μM) and CHIR99021 (3μM) on day 1, CDM-PVA supplemented with Activin (100ng/ml), FGF2 (80ng/ml), BMP4 (10ng/ml) and Ly294002 (10μM) on day 2 then RPMI/B27 media containing Activin (100ng/ml), FGF2 (80ng/ml) on day 3. For primitive gut tube differentiation (days 4-6), cells were cultured in Adv-BSA media supplemented with SB-431542 (10μM), FGF10 (50ng/ml), RA (3μM), Noggin (150μg/ml) and L-Ascorbic acid (250μM) for 3 days. For posterior foregut differentiation (days 7-8), cells were cultured in Adv-BSA with FGF10 (50ng/ml), RA (3μM), Noggin (150μg/ml), KAAD-cyclopamine (0.238μM), PdBU (50nM) and L-Ascorbic acid (250μM). Pancreatic progenitor specification (days 9- 13) was induced by culturing cells in RA (1μM), Noggin (150μg/ml), KAAD-cyclopamine (200ng/ml), EGF (100ng/ml), Nicotinamide (10mM), and L-Ascorbic acid (250μM) for 5 days. Cells were then grown in Adv-BSA containing glucose (final concentration 25mM), B27 (1%), RA (100nM), DAPT (1μM), Alk5i (10μM) and the small molecule BNZ (0.1mM) for 3 days to induce maturation of pancreatic progenitors to endocrine progenitor cells (fifth stage; days 14-16). For maturation of endocrine cells and further differentiation into C-peptide-producing beta cells, cells were cultured for 3 additional days in Adv-BSA containing B27 (1%), RA (100nM) and Alk5i (10μM) followed by 11 days in Adv-BSA containing B27 (1%), RA (100nM) (sixth stage; days 17-27).

List of abbreviations used in **Figure 1A**: A, activin A; F, fibroblast growth factor 2; B, bone morphogenetic protein; Ly, LY294002; Chir, Chir99021; B27, B-27 Supplement® (ThermoFisher Scientific, Waltham, MA, USA); RA; retinoic acid; NOG, noggin; SB; SB-431542; F10, fibroblast growth factor 10; VitC, Vitamin C; Cyclo, cyclopamine; PdBU, phorbol 12,13-dibutyrate; EGF, epidermal growth factor; NA, nicotinamide; Alk5i, TGFβ type I receptor kinase (Alk5) inhibitor; BNZ, 6-Benzoyladenosine-3',5'-cyclic monophosphate; DAPT, N-(N [3,5-diflurophenylacetyl]-L-alanyl)-S-phenylglycine t-butyl ester. CDM, chemically defined medium; PVA, Polyvinyl Alcohol; RPMI, Roswell Park Memorial Institute medium; Adv-DMEM/F-12; Advanced Dulbecco's Modified Eagle Medium/Ham's F-12.

Generation of clonal hiPSC mutant lines

Assembly of Cas9, gRNA and donor vectors. Cas9 nuclease target regions in exon 1 of the HNF1B gene and suitable guide RNA (gRNA) sequences were identified using the CRISPR design tool provided by the Zhang laboratory (Ran et al., 2013). The human codon-optimized Cas9 expression plasmid was obtained from Addgene (hCas9 Plasmid #41815,). To construct the gRNA vector, gRNA sequences were cloned into a U6 BsaI gRNA backbone vector, containing a hU6 promoter and a Kanamycin resistance cassette (obtained from Professor Bill Skarnes' group at the Wellcome Trust Sanger Institute, Hinxton, Cambridge). The success of the gRNA assembly was verified by Sanger sequencing.

A donor vector aimed at terminating transcription of HNF1B prematurely by inserting a 'donor template' through homologous recombination was also constructed. The donor vector contains 5' and 3' homology arms each 1kb in length recognising the flanking regions of the gRNA target site, an Ef1a promoter, a puromycin antibiotic resistant cassette and a polyA tail. The final construct was sequenced to confirm that the donor vector was cloned successfully.

Electroporation and screening of drug-resistant clones. Cas9 nuclease, gRNA and final donor vectors were transfected into cells using the Amaxa Nucleofector® Technology and Human Stem Cell Nucleofector® Kit 1 (Lonza, #VAPH-5012). Cells were harvested into a single cell suspension using Accutase (Stem Cell Technologies #07920). 1 x 10^6 cells were used for each nucleofection. Nucleofection was performed according to the manufacturer's instructions using Nucleofector® Program B-016. Following nucleofection, cells were plated in E8 media supplemented with ROCK inhibitor. 48 hours after nucleofection, selection was commenced using puromycin (1 µg/ml; Sigma-Aldrich, #P8833) for 5 days. Single colonies were picked and screened to detect wild-type, heterozygous and homozygous HNF1B knockout clones.

Multiplex fluorescence in situ hybridization (M-FISH) karyotyping

For each cell line, 10-20 randomly selected metaphases were karyotyped based on multiplex fluorescence *in situ* hybridization (M-FISH) with human 24-colour painting probe and DAPI-banding pattern analyses.

Western blotting

Cells were harvested and lysed using the cell lysis buffer, CelLytic M reagent (Sigma-Aldrich, C2978) with PhosStop phosphatase inhibitor cocktail (Roche, # 4906837001) and cOmplete protease inhibitor cocktail (Roche, #11697498001). Cell lysates were centrifuged at 14,000 rpm at 4°C for 5 minutes. The supernatants were collected and protein concentrations were determined by Bradford assay (Protein Assay Dye Reagent Concentrate, Bio-Rad) according to the manufacturer's protocol. The normalized cell lysates were heat denatured then subjected to SDS-PAGE electrophoresis on NuPAGE Novex 4-12% Bis-Tris Protein Gels using the XCell SureLock Mini-Cell (Invitrogen) system. The protein samples were next transferred onto a PVDF membrane (Bio-Rad, #162-0177) using the Mini Trans-Blot Cell (Bio-Rad). Membranes were blocked in 4% powdered skimmed milk diluted in 0.05% Tween-20 in D-PBS for 1 hour at room temperature. The membrane was incubated with primary antibodies overnight at 4°C, followed by incubation with horseradish peroxidase (HRP)-conjugated secondary antibodies for 1 hr at room temperature. Protein bands were detected via chemiluminescence using the Pierce ECL detection kit (Thermo Fisher Scientific). Antibodies used for western blotting are listed below.

Immunofluorescence (IF) staining

Cells in 12 well plates were fixed in 500 µl of 4% paraformaldehyde (PFA; VWR, #43368.9M) solution for 20 min at 4°C. They were then washed thrice in D-PBS followed by blocking in 10% donkey serum (AbD Serotec, #C06SB) in 0.1% Triton X-100 in D-PBS (PBST) for 20 min at room temperature. Cells were then incubated overnight at 4°C with primary antibodies diluted in 1% donkey serum in PBST. Cells were then washed thrice with PBST and incubated with fluorescence-dye conjugated secondary antibodies diluted in 1% donkey serum in PBST for 1 hr

at room temperature. Antibodies used for immunostaining are listed below. Images were taken using a Zeiss LSM 700 confocal microscope (Carl Zeiss, Jena, Germany).

Fluorescence activated cell sorting (FACS) analysis

Cells in 12 well plates were washed twice in D-PBS and incubated in Accutase for 5 min at 37°C. The cells were dissociated by gentle pipetting and then re-suspended and washed twice with D-PBS. The cell suspension was filtered through a 40μm filter and then fixed by incubating in 4% PFA solution diluted in D-PBS for 20 min at 4°C, then washed twice in D-PBS. Cell surface marker staining; CXCR4 or Live/dead staining (LIVE⁄DEAD Fixable Violet Dead Cell Stain Kit; Molecular Probes, #L34955, 1:1,000); was performed by incubating cells in FACS buffer or 5% FBS in D-PBS for 30 minutes or 1 hour on ice. For intracellular staining, cells were permeabilised in 1% saponin (Sigma-Aldrich, #47036-50G-F) in D-PBS for 30 min at room temperature. Cells were then incubated with primary antibody diluted in staining solution (1% saponin and 5% FBS in D-PBS) for 2 hr at room temperature. Cells were washed three times with staining solution and incubated with secondary antibodies diluted in staining solution for 30 min at room temperature. Cells were washed thrice in staining solution and re-suspended in 2% FBS diluted in D-PBS prior to analysis. Analyses were performed using a BD LRSFortessa cell analyser (BD Biosciences). Data analyses were performed using FlowJo. All gates shown on scatterplots were set according to the undifferentiated population control. Antibodies used for FACS analyses are listed below.

Antibodies used in this work

The antibodies used in this work for western blotting, IF and/or FACS included: Goat anti-NANOG (R&D, AF1997, RRID:AB_355097), Goat anti-OCT3/4 (Santa Cruz, sc8628, RRID:AB_653551), Mouse anti-OCT3/4 (Santa Cruz, sc5279, RRID:AB_628051), Goat anti-SOX2 (R&D, AF2018, RRID:AB_355110), Rabbit anti-SOX2 (Millipore, AB5603, RRID:AB_2286686), Goat anti-SOX17 (R&D, AF1924, RRID:AB_355060), Goat anti-Brachyury (R&D, AF2085,

RRID:AB_2200235), Rabbit anti-PAX6 (Cambridge BioScience , PRB-278P-100, RRID:AB_291612), Rabbit anti-EOMES (Abcam, ab23345, RRID:AB_778267), Goat anti-FOXA2 (R&D, AF2400, RRID:AB_2294104), Mouse anti-CXCR4 (R&D, MAB173, RRID:AB_2089398), Mouse anti-GATA4 G-4 (Santa Cruz, sc25310, RRID:AB_627667), Rabbit anti-GATA6 D61E4 (CST, #5851, RRID:AB_10705521), Goat anti-HNF1B C-20 (Santa Cruz, sc7411, RRID:AB_2116769), Rabbit anti-HNF1B (Santa Cruz, sc22840, RRID:AB_2279595), Rabbit anti-HNF4A H-171 (Santa Cruz, sc8987, RRID:AB_2116913), Mouse anti-HEX (Abcam, ab117864, RRID:AB_10900199), Mouse anti-CDX2 (CDX-88) (Abcam, ab86949, RRID:AB_10671889), Goat anti-PDX1 (R&D, AF2419, RRID:AB_355257), Rabbit anti-PDX1 (CST, #5679, RRID:AB_10706174), Rabbit anti-HNF6 H-100 (Santa Cruz, sc13050, RRID:AB_2251852), Rabbit anti-SOX9 (Millipore, AB5535, RRID:AB_2239761), Mouse anti-NKX6-1 (DSHB, F55A12, RRID:AB_532379), Sheep anti-NGN3 (R&D, AF3444, RRID:AB_2149527), Goat anti-GCC G-17 (Santacruz, sc7780, RRID:AB_641025), Rabbit anti-SST (Daka, A0566, RRID:AB_2688022), Mouse anti-C-PEP (Acris Antibodies, BM270S, RRID:AB_978884), Rat anti-INS (DSHB, GN-ID4, RRID:AB_2255626), Goat anti-CHGA (Santa Cruz, sc1488, RRID:AB_2276319), Mouse anti-Alpha-tubulin (Sigma, T6199, RRID:AB_477583), Donkey anti-goat 488 (Invitrogen, A-11055, RRID:AB_2534102), Donkey anti-mouse 488 (Invitrogen, A-21202, RRID:AB_141607), Donkey anti-rat 488 (Invitrogen, A-21208, RRID:AB_141709), Donkey anti-sheep 488 (Invitrogen, A-11015, RRID:AB_141362), Donkey anti-rabbit 488 (Invitrogen, A-21206, RRID:AB_2535792), Donkey anti-goat 647 (Invitrogen, A-21447, RRID:AB_141844), Donkey anti-mouse 647 (Invitrogen, A-31571, RRID:AB_162542), Donkey anti-sheep 647 (Invitrogen, A21448, RRID:AB_1500712), Donkey anti-rabbit 647 (Invitrogen, A-31573, RRID:AB_2536183), Goat anti-mouse HRP (Sigma, A2554, RRID:AB_258008), Goat anti-rabbit HRP (Sigma, A0545, RRID:AB_257896).

RNA isolation and qRT-PCR

Total RNA was isolated using the RNeasy Mini Kit (Qiagen, #74106) and eluted in 30 µl of RNase free water. 500 ng of isolated total RNA was used for cDNA synthesis with SuperScript II Reverse Transcriptase (Invitrogen, #18064014). Quantitative Real-Time PCR was performed using the SensiMix SYBR Low-Rox Kit (Bioline, #QT625-20) on the Mx3005P Real-Time PCR system (Agilent) according to the manufacturer's instructions. Samples were run in technical triplicates and normalized to PBGD. Gene-specific primers are listed below:

**Gene**	**Primer**	**Sequence (5' to 3')**
PBGD	F	GGAGCCATGTCTGGTAACGG
	R	CCACGCGAATCACTCTCATCT
POU5F1 / OCT4	F	AGTGAGAGGCAACCTGGAGA
	R	ACACTCGGACCACATCCTTC
NANOG	F	CATGAGTGTGGATCCAGCTTG
	R	CCTGAATAAGCAGATCCATGG
SOX2	F	TGGACAGTTACGCGCACAT
	R	CGAGTAGGACATGCTGTAGGT
SOX17	F	CGCACGGAATTTGAACAGTA
	R	GGATCAGGGACCTGTCACAC
CXCR4	F	CACCGCATCTGGAGAACCA
	R	GCCCATTTCCTCGGTGTAGTT
GATA6	F	TTCGTTTCCTGGTTTGAATTCC
	R	TGCAATGCTTGTGGACTCTAC
Brachyury	F	TGCTTCCCTGAGACCCAGTT
	R	GATCACTTCTTTCCTTTGCATCAAG
PAX6	F	CTTTGCTTGGGAAATCCGAG
	R	AGCCAGGTTGCGAAGAACTC
FOXA2	F	GGGAGCGGTGAAGATGGA
	R	TCATGTTGCTCACGGAGGAGTA
HNF1B	F	TCACAGATACCAGCAGCATCAGT
	R	GGGCATCACCAGGCTTGTA
HNF4A	F	CATGGCCAAGATTGACAACCT
	R	TTCCCATATGTTCCTGCATCAG
HEX	F	GCCCTTTTACATCGAGGACA
	R	AGGGCGAACATTGAGAGCTA
ONECUT1	F	GTGTTGCCTCTATCCTTCCCAT
	R	CGCTCCGCTTAGCAGCAT
PTF1A		Hs_PTF1A_1_SG QuantiTect Primer Assay, Quiagen QT0021802
SOX9		Hs_SOX9_1_SG QuantiTect Primer Assay, Qiagen QT00001498
MNX1	F	CACCGCGGGCATGATC
	R	ACTTCCCCAGGAGGTTCGA
PDX1	F	AAGTCTACCAAAGCTCACGCG

	R	GTAGGCGCCGCCTGC
NKX6-1	F	GGCCTGTACCCCTCATCAAG
	R	TCCGGAAAAAGTGGGTCTCG
NEUROG3	F	GCTCATCGCTCTCTATTCTTTTGC
	R	GGTTGAGGCGTCATCCTTTCT
NEUROD1	F	AGACGCTTTGCAAGGGCTTA
	R	TCCGAGGATTGAGTTGCAGG
GLIS3	F	GTCCATGGATTTTATGGGCAGC
	R	CAAACGAAGGCACCACACTG
CHGA	F	GCAGAGGACCAGGAGCTAGA
	R	CAGGGGCTGAGAACAAGAGA
INS	F	CAGGAGGCGCATCCACA
	R	AAGAGGCCATCAAGCAGATCA
GCG	F	AAGCATTTACTTTGTGGCTGGATT
	R	TGATCTGGATTTCTCCTCTGTGTCT
SST	F	CCCCAGACTCCGTCAGTTTC
	R	TCCGTCTGGTTGGGTTCAG

RNA-seq experiments

For the bulk RNA-seq experiments, one HNF1B$^{+/+}$, one HNF1B$^{+/-}$ and one HNF1B$^{-/-}$ (targeted wild-type) clone from the FSPS13.B hiPSC line were differentiated along the pancreatic lineage. RNA was extracted using the GenElute Mammalian Total RNA Miniprep Kit (Sigma-Aldrich) according to manufacturer's protocol. RNA-seq library construction and sequencing was carried out by the DNA pipelines core facility at the Wellcome Sanger Institute. Standard Illumina unstranded poly-A enriched libraries were prepared and sequenced using Illumina HiSeq 2500 v4 (Illumina, San Diego, CA, USA), with 75bp paired-end reads per sample and a library fragment size of 100-1000 bp. Three independent experiments (biological triplicates generated from FSPS13.B clones) were sequenced for each clone at each stage of differentiation.

RNA-seq data analysis and functional annotation

Bioinformatics analyses were carried out following standard procedures (Conesa et al., 2016). Reads were aligned to the reference human genome assembly GRCh38 with TopHat v2.0.13 (Kim et al., 2013) with a transcript index built using Ensembl version 76 gene annotation. Reads

with Mapping Quality Value <10 were filtered out with Samtools. featureCounts was used on paired-end reads to count fragments in annotated gene features, with parameters '-p -C -T 8 -t exon -g gene_id' (Liao et al., 2014), and genes with no counts were filtered out. Differentially expressed genes were identified using DESeq2 R/Bioconductor package (Love et al., 2014). Genes with fold change ≥2 and adjusted p-value (using Benjamin-Hochberg correction) <0.05 were identified as differentially expressed (**Table S3**). Counts were normalized using the fragments per kilobase of transcript per million mapped reads (FPKM). Principal component plot of the samples was performed in DESeq2. Sample information together with the total number of aligned fragments and mapped reads with quality score ≥10 are shown in **Table S2**. For further analysis (**Figure 4**), differentially expressed genes were then selected if the three replicates had an expression value >0.5 FPKM in at least one of the genotypes. Functional annotation was performed with DAVID (Huang da et al., 2009), using the Gene Ontology (GO) tool, focusing in the biological processes (BP). Significantly enriched terms were defined with a Benjamini value >0.05 (**Table S4**).

Apoptosis assay

Apoptotic cells were detected using the Annexin V Apoptosis Detection Kit FITC (Thermo Fisher Scientific, Waltham, MA, USA). Cells were detached and harvested using Accutase and washed once in PBS, then once in 1x Binding Buffer. Cells were filtered and then resuspended in 1x Binding Buffer at 2.5x106 cells/ml. Cells were subsequently aliquoted in four conditions – Annexin V only, Annexin V and Propidium Iodide (PI), PI only, and no staining. 5μL of fluorochrome-conjugated Annexin V was added to 100μl of the cell suspension and incubated for 15 minutes at room temperature. After two washes with 1x Binding Buffer, cells were incubated for 10 minutes with 5μL PI (10μg/ml) to visualize dead cells. Cells were analysed by flow cytometry within four hours of staining. Flow cytometric data were analysed with FlowJo software.

Cell proliferation assay

Cell proliferation was measured using the Click-iT® EdU Flow Cytometry Assay Kit (Invitrogen). 5-ethynyl-2'-deoxyuridine (EdU) was added to the culture medium at 10µM for 2 hours. Cells were then detached and harvested using Accutase as previously discussed. 1x106 cells were aliquoted and washed once with 3mL of 1% BSA in PBS. Cells were then incubated in 100µL of Click-iT fixative for 15 minutes at room temperature, protected from light. Cells were washed with 3mL of 1% BSA in PBS and incubated in 100µl of 1x Click-iT saponin based permeabilization and wash reagent for 15 minutes. 500µl of Click-iT reaction cocktail (CuSO4 10µl, Pacific Blue azide 2.5µl, 1x Reaction Buffer Additive 50µl, and PBS 438µl) was added to each sample and the reaction mixture was incubated for 30minutes at room temperature, protected from light. Cells were washed once with 3mL of 1x Click-iT saponin based permeabilization and wash reagent and then resuspended in 1mL of 1x Click-iT saponin based for staining for DNA content using the FxCycle™ Far Red stain (Invitrogen). 1µl of FxCycle™ Far Red stain (final concentration 200nM) and 20mg/mL of RNase A was added to each flow cytometry sample and incubated for 30 minutes at room temperature. FxCycle™ Far Red stains RNA as well as dsDNA, so addition of RNase A (Thermo Fisher Scientific) is required for DNA content analysis. Samples were analysed on a flow cytometer without washing, using 405/450nm (Pacific Blue) and 640/670nm (Far Red) excitation and emission spectra.

Glucose-stimulated insulin secretion

Human iPSC-derived pancreatic endocrine cells at day 27 were preincubated in DMEM supplemented with 2.5µM glucose (low glucose media) for 60 minutes at 37°C. To measure basal C-peptide release, cells were incubated in low glucose media for 60 minutes at 37°C. To estimate glucose-induced C-peptide secretion, the media was replaced by DMEM supplemented with 22.5 mM glucose (high glucose media) and alternatively with DMEM supplemented with 2.5 mM glucose for 60 minutes at 37°C. The low glucose – high glucose stimulation was repeated for a second cycle. Finally, cells were incubated in low-glucose DMEM with 30mM potassium

chloride (KCl) for 30 min. The supernatant at each stage was collected and stored at -80°C for determination of C-peptide release. C-peptide ELISA was measured using the Mercodia C-peptide ELISA kit (Mercodia, Uppsala, Sweden), following the manufacturer's recommendations. Absorbance was read at 450nm on an Infinite 200 Pro plate reader (Tecan, Männedorf, Switzerland).

Preparation of differentiated cells for single-cell sequencing

Single cell libraries from D13.1βHet and D13.1βWT samples were generated using the Chromium Single Cell 3′ library and gel bead kit v2 (PN #120237) from 10x Genomics. Briefly, to reach a target cell number of 2,000 cells per sample, 3,500 cells per sample were loaded onto a channel of the 10x chip to produce Gel Bead-in-Emulsions (GEMs). This underwent reverse transcription to barcode RNA before cleanup and cDNA amplification followed by enzymatic fragmentation and 5′adaptor and sample index attachment. Libraries were sequenced on the HiSeq4000 (Illumina) with 125 bp paired-end sequencing.

Analysis of single cell RNA-seq data

Filtering, alignment to the GRCh38 human genome version 28 (Ensembl 92) and unique molecular identifier (UMI)-collapsing were performed using the Cell Ranger (v2.01) pipeline with default mapping arguments (10X Genomics). All further analyses were run with python 3 using the Scanpy API package (Wolf et al., 2018). To further remove low quality cells, we filtered cells with a high fraction of counts from mitochondrial genes (7% or more) indicating stressed or dying cells, and cells expressing less than 3000 genes. In addition, genes with expression in less than 10 cells were excluded. To improve the quality of the analysis, we also excluded outlier cells with >7000 genes detected, or with less that 1.5% fraction of counts from mitochondrial genes. Cell by gene count matrices of D13-1βWT and D13-1βHet samples were then concatenated to a single matrix and each cell was then normalized by total counts over all genes,

so that every cell has the same total count after normalization. Values were next log transformed. This output matrix was input to all further analyses.

Low dimensional embedding, visualization and clustering. A single-cell neighbourhood graph was computed on the 30 first principal components using 40 nearest neighbours. Clusters were identified using the Louvain algorithm (with resolution = 0.4) as implemented in louvain-igraph (https://github.com/vtraag/louvain-igraph) and adopted by Scanpy. Visualisation in 2D was performed using the dimensionality reduction algorithm UMAP (Uniform Manifold Approximation and Projection). Cell types were annotated based on the expression of known marker genes.

Marker gene identification and subtype characterization. Characteristic gene signatures were identified by testing for differential expression of a subgroup against all other cells or between two subgroups as outlined in the text using the tl.rank_genes_groups function of Scanpy, setting the Wilcoxon rank-sum test as statistical method.

Pseudotime analysis. To infer a pseudotemporal ordering of the cells, diffusion pseudotime (dpt) (Haghverdi et al., 2016) was used as implemented in Scanpy (tl.dpt) setting a root cell within the starting population (SOX2+ cluster).

Software specifications. All analyses from UMI count matrices were run with python 3 with the Scanpy API v.1.4 and anndata v.0.6.19. Versions of packages required by Scanpy that might influence numerical results are as follows: numpy v.1.16.2, scipy v.1.2.1, pandas v.0.24.1, scikit-learn v.0.20.2, statsmodels v.0.9.0, python-igraph v.0.8.2, louvain v.0.6.1.

Analysis of ChIP-seq data

Publicly available raw datasets were obtained from the Sequence Read Archive (SRA) database as listed in **Table S2**. Raw sequence reads were aligned to the human (UCSC hg19) genome using Bowtie v1.1.2 (Langmead et al., 2009), and further processed as previously described (Cebola et al., 2015; Pasquali et al., 2014). In brief, only sequences uniquely aligned with ≤1 mismatch were retained. Post-alignment processing of sequence reads included *in silico*

extension and signal normalization based on the number of million mapped reads. Reads were extended to a final length equal to MACS fragment size estimation (Zhang et al., 2008), and only unique reads were retained. For signal normalization, the number of reads mapping to each base in the genome was counted using the genomeCoverageBed command from BedTools (Quinlan and Hall, 2010). Processed files were visualized in the UCSC genome browser (Kent et al., 2002). Transcription factor enrichment sites were detected with MACS v1.4.2 (Zhang et al., 2008) using default parameters and a *P* value of 10^{-5}. Transcription factor binding sites were associated to the nearby genes using GREAT v4.0.4 with default settings

Quantification and statistical analysis

For both FSPS13.B and Eipl_1, we used 3 wild-type clones (HNF1B+/+; one non-targeted wild-type and two targeted wild-type clones), 3 heterozygous clones (HNF1B+/-) and 2 homozygous clones (HNF1B-/-; one with puromycin cassette in both alleles and one with puromycin cassette in 1st allele and indel in 2nd allele). The clone identities are shown in **Figures S2C, S2E**. The data in the main and supplementary figures are pooled from experiments using FSPS13.B and Eipl_1 clones for qPCR, flow cytometry and Elisa. Quantification data are presented as mean ± SEM. Data from clonal lines of the same genotype were combined for calculating the significance of the differences between different genotypes. To directly compare two groups, Student's t test with two-tailed distribution was used to test for statistical significance. P values less than 0.05 was considered statistically significant. All statistical analyses were performed using GraphPad Prism 6.0 (GraphPad Software Inc., San Diego, CA, USA) or the R statistical environment.

REFERENCES

Cebola, I., Rodriguez-Segui, S.A., Cho, C.H., Bessa, J., Rovira, M., Luengo, M., Chhatriwala, M., Berry, A., Ponsa-Cobas, J., Maestro, M.A., *et al.* (2015). TEAD and YAP regulate the enhancer network of human embryonic pancreatic progenitors. Nat Cell Biol *17*, 615-626.

Conesa, A., Madrigal, P., Tarazona, S., Gomez-Cabrero, D., Cervera, A., McPherson, A., Szcześniak, M.W., Gaffney, D.J., Elo, L.L., Zhang, X., *et al.* (2016). A survey of best practices for RNA-seq data analysis. Genome Biology *17*, 13.

Haghverdi, L., BÃ¼ttner, M., Wolf, F.A., Buettner, F., and Theis, F.J. (2016). Diffusion pseudotime robustly reconstructs lineage branching. Nature Methods *13*, 845-848.

Huang da, W., Sherman, B.T., and Lempicki, R.A. (2009). Systematic and integrative analysis of large gene lists using DAVID bioinformatics resources. Nat Protoc *4*, 44-57.

Kent, W.J., Sugnet, C.W., Furey, T.S., Roskin, K.M., Pringle, T.H., Zahler, A.M., and Haussler, D. (2002). The human genome browser at UCSC. Genome Res *12*, 996-1006.

Kim, D., Pertea, G., Trapnell, C., Pimentel, H., Kelley, R., and Salzberg, S.L. (2013). TopHat2: accurate alignment of transcriptomes in the presence of insertions, deletions and gene fusions. Genome Biology *14*, R36.

Langmead, B., Trapnell, C., Pop, M., and Salzberg, S.L. (2009). Ultrafast and memory-efficient alignment of short DNA sequences to the human genome. Genome Biol *10*, R25.

Liao, Y., Smyth, G.K., and Shi, W. (2014). featureCounts: an efficient general purpose program for assigning sequence reads to genomic features. Bioinformatics *30*, 923-930.

Love, M.I., Huber, W., and Anders, S. (2014). Moderated estimation of fold change and dispersion for RNA-seq data with DESeq2. Genome Biology *15*, 550.

Pasquali, L., Gaulton, K.J., Rodriguez-Segui, S.A., Mularoni, L., Miguel-Escalada, I., Akerman, I., Tena, J.J., Moran, I., Gomez-Marin, C., van de Bunt, M., *et al.* (2014). Pancreatic islet enhancer clusters enriched in type 2 diabetes risk-associated variants. Nat Genet *46*, 136-143.

Quinlan, A.R., and Hall, I.M. (2010). BEDTools: a flexible suite of utilities for comparing genomic features. Bioinformatics *26*, 841-842.

Wolf, F.A., Angerer, P., and Theis, F.J. (2018). SCANPY: large-scale single-cell gene expression data analysis. Genome Biol *19*, 15.

Zhang, Y., Liu, T., Meyer, C.A., Eeckhoute, J., Johnson, D.S., Bernstein, B.E., Nusbaum, C., Myers, R.M., Brown, M., Li, W., *et al.* (2008). Model-based analysis of ChIP-Seq (MACS). Genome Biol *9*, R137.
