# Supplementary material for: Modeling HNF1B-associated monogenic diabetes using human iPSCs reveals an early stage impairment of the pancreatic developmental program
Source: Stem Cell Reports. 2021 Aug 26;16(9):2289–304. doi: 10.1016/j.stemcr.2021.07.018 (PMC8452540; doi:10.1016/j.stemcr.2021.07.018)
Supplement: Document S2. Article plus supplemental information [file mmc6.pdf]

Stem Cell Reports
Article
OPEN ACCESS

Modeling HNF1B-associated monogenic diabetes using human iPSCs reveals an early stage impairment of the pancreatic developmental program

Ranna El-Khairi,[1,6,2] Evelyn Olszanowski,[3,4] Daniele Muraro,[1,2] Pedro Madrigal,[1,2] Katarzyna Tilgner,[2] Mariya Chhatriwala,[1,2] Sapna Vyas,[2] Crystal Y. Chia,[1,2] Ludovic Vallier,[1,2,6,7,*] and Santiago A. Rodríguez-Seguí[3,5,7,*]
[1]Wellcome Medical Research Council Cambridge Stem Cell Institute, Anne McLaren Laboratory for Regenerative Medicine, University of Cambridge, Cambridge, UK
[2]Wellcome Sanger Institute, Hinxton, Cambridge, UK
[3]Instituto de Fisiología, Biología Molecular y Neurociencias (IFIBYNE), CONICET-Universidad de Buenos Aires, Ciudad Universitaria, Buenos Aires, Argentina
[4]Departamento de Química Biológica, Facultad de Ciencias Exactas y Naturales, Universidad de Buenos Aires, Buenos Aires, Argentina
[5]Departamento de Fisiología, Biología Molecular y Celular, Facultad de Ciencias Exactas y Naturales, Universidad de Buenos Aires, Buenos Aires, Argentina
[6]Department of Surgery, University of Cambridge, Cambridge, UK
[7]These authors contributed equally
*Correspondence: lv225@cam.ac.uk (L.V.), srodriguez@fbmc.fcen.uba.ar (S.A.R.-S.)
https://doi.org/10.1016/j.stemcr.2021.07.018

SUMMARY

Heterozygous mutations in *HNF1B* in humans result in a multisystem disorder, including pancreatic hypoplasia and diabetes mellitus. Here we used a well-controlled human induced pluripotent stem cell pancreatic differentiation model to elucidate the molecular mechanisms underlying HNF1B-associated diabetes. Our results show that lack of HNF1B blocks specification of pancreatic fate from the foregut progenitor (FP) stage, but HNF1B haploinsufficiency allows differentiation of multipotent pancreatic progenitor cells (MPCs) and insulin-secreting β-like cells. We show that HNF1B haploinsufficiency impairs cell proliferation in FPs and MPCs. This could be attributed to impaired induction of key pancreatic developmental genes, including *SOX11*, *ROBO2*, and additional TEAD1 target genes whose function is associated with MPC self-renewal. In this work we uncover an exhaustive list of potential *HNF1B* gene targets during human pancreas organogenesis whose downregulation might underlie HNF1B-associated diabetes onset in humans, thus providing an important resource to understand the pathogenesis of this disease.

INTRODUCTION

Maturity-onset diabetes of the young (MODY) is the most common form of monogenic diabetes, and it is characterized by autosomal dominant inheritance, onset typically before 25 years of age, and hyperglycemia due to β cell failure. In particular, hepatic nuclear factor 1β (HNF1B), associated with MODY5 (Horikawa et al., 1997), plays an important role in the normal development of the kidney, liver, pancreas, bile ducts, and urogenital tract, through tissue-specific regulation of gene expression in these organs (Barbacci et al., 1999; Coffinier et al., 1999). In humans, heterozygous mutations in *HNF1B* result in a multisystem disorder. The most common clinical features include renal disease, pancreatic hypoplasia, and diabetes mellitus, which typically develops during adolescence or early adulthood.

More than 50 splice-site, nonsense, missense, and frameshift mutations in the *HNF1B* gene have been reported to date, as well as partial or whole gene deletions (Clissold et al., 2014). Patients with whole-gene deletions do not exhibit a phenotype different from those with coding or splice-site mutations, thus suggesting that dysfunction is due to a gene-dosage effect, i.e., haploinsufficiency (Bellanne-Chantelot et al., 2005; Edghill et al., 2006b). Haploinsufficiency is an important contributor to human disease; however, the mechanism by which a reduced dosage of a transcription factor affects downstream target genes to cause a disease is poorly understood, mostly due to the lack of an appropriate model system.

The pathophysiology of diabetes mellitus in patients with *HNF1B* mutations is mainly attributed to β cell dysfunction and reduced insulin secretion, which is likely to be a consequence of pancreatic hypoplasia. Interestingly, mouse models often do not recapitulate the disease phenotype in humans. As an example, mice with heterozygous deletions of *Hnf1a*, *Hnf4a*, or *Hnf1b* do not develop diabetes (El-Khairi and Vallier, 2016; Harries et al., 2009; Lau et al., 2018). This species divergence and the difficulty in accessing patient samples led us to model HNF1B deficiency *in vitro* using human pluripotent stem cells (hPSCs). Several studies in the past decade have used genetically engineered hPSC culture systems for differentiation into pancreatic cells to further expand our understanding of the roles of various genes in pancreas development and function (recently reviewed in Burgos et al., 2021).

In this study, we established a well-controlled human induced pluripotent stem cell (hiPSC) pancreatic differentiation model to elucidate the molecular mechanisms underlying HNF1B-associated diabetes and pancreatic

This is an open access article under the CC BY license (http://creativecommons.org/licenses/by/4.0/).

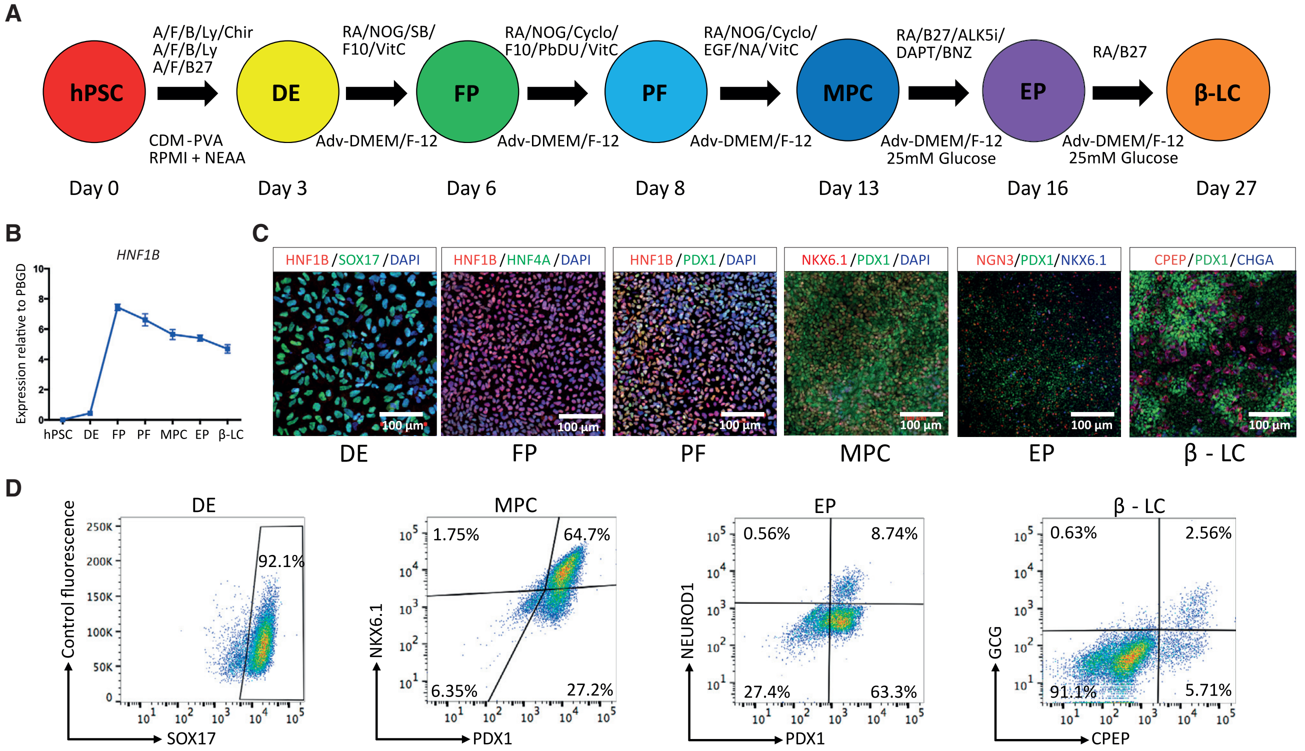

Figure 1. HNF1B expression during hiPSC pancreatic differentiation

(A) Overview of the protocol used to differentiate hiPSCs into pancreatic β-like cells. hPSC, human induced pluripotent stem cell; DE, definitive endoderm, FP, foregut progenitor; PF, posterior foregut; MPC, multipotent pancreatic progenitor cell; EP, endocrine progenitor; β-LC, β-like cell. A, activin A; F, fibroblast growth factor 2; B, bone morphogenetic protein; CDM, chemically defined medium. Refer to the supplemental experimental procedures for additional abbreviations.

(B) The mRNA expression pattern for *HNF1B* during hPSC differentiation into pancreatic β-like cells. The mRNA levels were measured by qRT-PCR (n = 5 independent experiments at each stage of differentiation using the FSPS13.B wild-type clone) and normalized to the housekeeping gene *PBGD*.

(C) Representative immunostaining of HNF1B and other stage-specific markers.

(D) Representative FACS dot plots of cells stained for the stage-specific markers SOX17, PDX1, NKX6.1, NEUROD1, CPEP, and GCG. The percentage of each cell population is indicated in the corresponding quadrant for all FACS plots.

hypoplasia. We generated isogenic HNF1B mutant lines to investigate the influence of HNF1B dosage on pancreatic differentiation. Our findings reveal that homozygous knockout of *HNF1B* resulted in failure of foregut and pancreatic progenitor development. Heterozygous knockout of *HNF1B*, on the other hand, resulted in impairment of pancreatic progenitor and endocrine cell production. Despite the lower efficiency in producing β-like cells, these were functional to the same extent as their counterparts derived from wild-type (WT) hiPSCs. RNA sequencing (RNA-seq) and in-depth transcriptomic analyses showed that low dosages of HNF1B in pancreatic progenitor cells alter their early stage specification, downregulating the expression of several genes with known or suspected roles in pancreas development. Our results are consistent with a model in which HNF1B haploinsufficiency impairs the expansion and maintenance of pancreatic progenitor cells *in vitro*. *In vivo*, this would likely result in reduced β cell numbers at birth and increased diabetes susceptibility later in life.

RESULTS

HNF1B is expressed during the *in vitro* differentiation of human iPSCs into the pancreatic lineage

Directed differentiation of hiPSCs into pancreatic cells was undertaken using a protocol developed in our laboratory (Figure 1A). This 27-day protocol, a revised version of an 18-day chemically defined protocol previously published by our group (Cho et al., 2012), was applied using two hiPSC lines (FSPS13.B and Eipl_1). Consistent with findings using our previous protocol, HNF1B expression was upregulated at the foregut progenitor (FP) stage (Figure 1B)

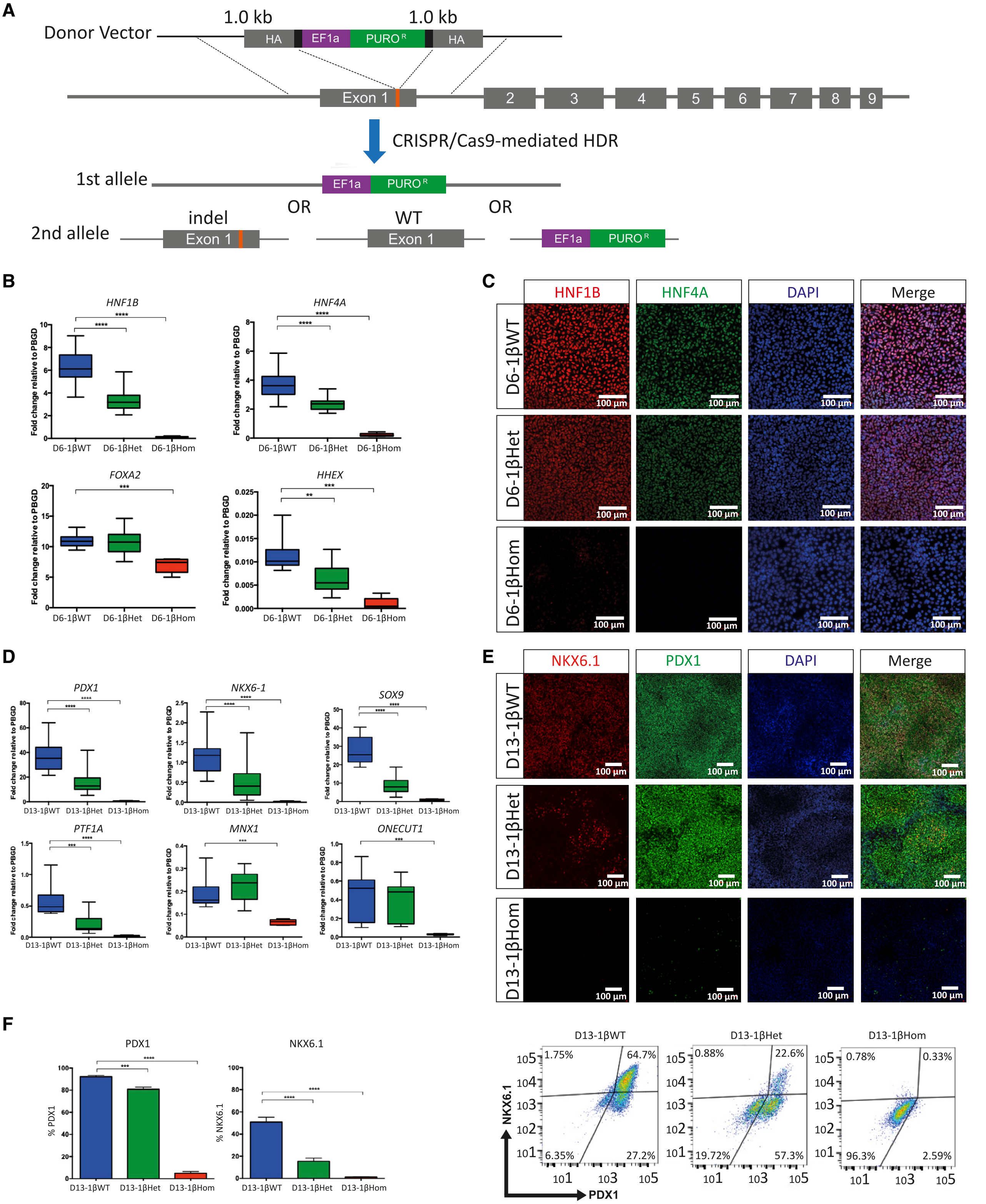

(legend on next page)

and was co-expressed with other FP markers such as HNF4A (Figures 1C and S1A–S1C, day 6). At the posterior foregut stage, PDX1^{+} cells co-expressing HNF1B were identified, but NKX6.1 was not detected (Figures 1C and S1A–S1C, day 8). At the pancreatic multipotent progenitor cell (MPC) stage, >90% PDX1^{+} cells were detected, and HNF1B was still co-expressed with PDX1 in almost all cells, and around 50% of cells co-expressed NKX6.1 (~60% in FSPS13.B and ~40% in Eipl_1) (Figures 1C, 1D, S1A–S1C and data not shown). At the endocrine progenitor (EP) and β-like cell stages the expression of HNF1B was decreased (Figure S1C). Consistent with these findings, Hnf1b expression is excluded from β cells at comparable stages in adult mice and is restricted to adult ductal cells (Haumaitre et al., 2005; Maestro et al., 2003; Nammo et al., 2008). Expression of the EP cell markers NGN3 and NEUROD1 peaked at day 16 (EP) and around 8%–10% of the cells expressed NEUROD1 at this stage (Figures 1C and 1D). Expression of pancreatic hormonal markers (chromogranin A, insulin, glucagon, and somatostatin) significantly increased at day 27. By day 27 around 7%–8% of the cells expressed CPEP and around 50%–60% of these cells were monohormonal (4%–5% of total cells) (Figures 1C, 1D, S1A–S1C). Taken together, these results show that our *in vitro* protocol of cell differentiation follows a natural path of development with HNF1B expression starting at the foregut stage.

HNF1B is required for efficient foregut and pancreatic progenitor formation

We then used the CRISPR-Cas9 genome editing system to generate homozygous and heterozygous HNF1B knockout hiPSCs (Figure 2A). Insertion of a puromycin-resistance cassette allowed for reliable selection of targeted clones and resulted in large disruptions in the open reading frame of the *HNF1B* gene in one or both alleles (Figures 2A, S2A, and S2B, Table S1). To control for potential CRISPR-Cas9 off-target effects and line-to-line variations, we analyzed six HNF1B heterozygous and four HNF1B homozygous hiPSCs for the FSPS13.B and Eipl_1 hiPSC lines, and compared them with six isogenic HNF1B WT control hiPSC lines. Notably, the expression of the pluripotency markers *NANOG*, *OCT4*, and *SOX2* was not affected in the hiPSCs edited with this approach (Figure S2C). Western blotting confirmed the absence of HNF1B protein in homozygous mutant lines at the FP stage (Figure S2D). We also detected reduced HNF1B expression in HNF1B heterozygous cells at the FP stage by qRT-PCR and immunostaining (Figures 2B, 2C, and S2E). Hereafter, we will refer to cells derived from the WT (HNF1B$^{+/+}$), heterozygous (HNF1B$^{+/-}$), and homozygous (HNF1B$^{-/-}$) mutant lines as 1βWT, 1βHet, and 1βHom, respectively, preceded by the differentiation stage day. Thus, D13-1βWT stands for day 13 cells derived from WT (HNF1B$^{+/+}$) hiPSCs, and so on.

A more detailed analysis of the differentiation outcomes for these cells revealed that D3-1βWT, D3-1βHet, and D3-1βHom hiPSCs could be differentiated into definitive endoderm cells expressing *SOX17* and *CXCR4* with comparable efficiencies, as determined by qRT-PCR (Figure S2F) and fluorescence-activated cell sorting (FACS) analysis for SOX17^{+} cells (Figure S2G). Subsequently, D6-1βHom mutant lines failed to differentiate to FP (HNF1B^{+}/HNF4A^{+}) cells, while HNF1B and HNF4A expression was significantly reduced in D6-1βHet cells compared with D6-1βWT cells (Figures 2B and 2C). Consistent with this, PDX1^{+}/NKX6.1^{-} and PDX1^{+}/NKX6.1^{+} cells failed to form in D8-1βHom and D13-1βHom cells, respectively (Figures 2D and 2E and data not shown). This is likely due to the earlier requirement for HNF1B at the FP stage. On the other hand, D13-1βHet mutant lines produced a lower number of

Figure 2. Derivation and characterization of HNF1B mutant hiPSC lines

(A) CRISPR guide RNA design for generating HNF1B mutants from the FSPS13.B parental line. The schematic shows the human *HNF1B* locus and indicates the CRISPR-Cas9 cut site, lying in exon 1. The target sequences of the CRISPR guide RNA and the corresponding protospacer-adjacent motif sequence are indicated in black and red, respectively. The "knock-in" vector introduces a puromycin-resistance cassette. Successful homologous recombination resulted in both heterozygous HNF1B$^{+/-}$ and homozygous HNF1B$^{-/-}$ mutant hiPSCs.

(B) Expression of *HNF1B*, *HNF4A*, *FOXA2*, and *HHEX* at the foregut progenitor stage of differentiation. The mRNA levels were measured by qRT-PCR and normalized to the housekeeping gene *PBGD*. Data were pooled from n = 5 independent experiments for each of the eight FSPS13.B clones and n = 3 independent experiments for each of the eight Eipl_1 clones, with clone identities as per Figure S2C. Student's t test with two-tailed distribution was used for statistical analysis. All data are presented as the mean ± SEM. $^{**}p < 0.01$, $^{***}p < 0.001$, and $^{****}p < 0.0001$.

(C) Representative immunofluorescence images showing wild-type HNF1B$^{+/+}$ (D6-1βWT), heterozygous HNF1B$^{+/-}$ (D6-1βHet), and homozygous HNF1B$^{-/-}$ (D6-1βHom) mutant cells at the foregut progenitor stage of differentiation.

(D) Expression of key pancreatic developmental genes in D13-1βWT, D13-1βHet, and D13-1βHom cells. The mRNA levels were measured by qRT-PCR and normalized to the housekeeping gene *PBGD*. Replicates and statistics are as indicated for (B).

(E) Representative immunostaining showing PDX1 and NKX6.1 co-expression at the pancreatic progenitor stage of differentiation in D13-1βWT, D13-1βHet, and D13-1βHom cells.

(F) FACS analysis of PDX1^{+}, NKX6.1^{+} pancreatic progenitor cells derived from 1βWT, 1βHet, and 1βHom hiPSCs at day 13 of the differentiation protocol and representative plots. Replicates and statistics are as indicated for (B).

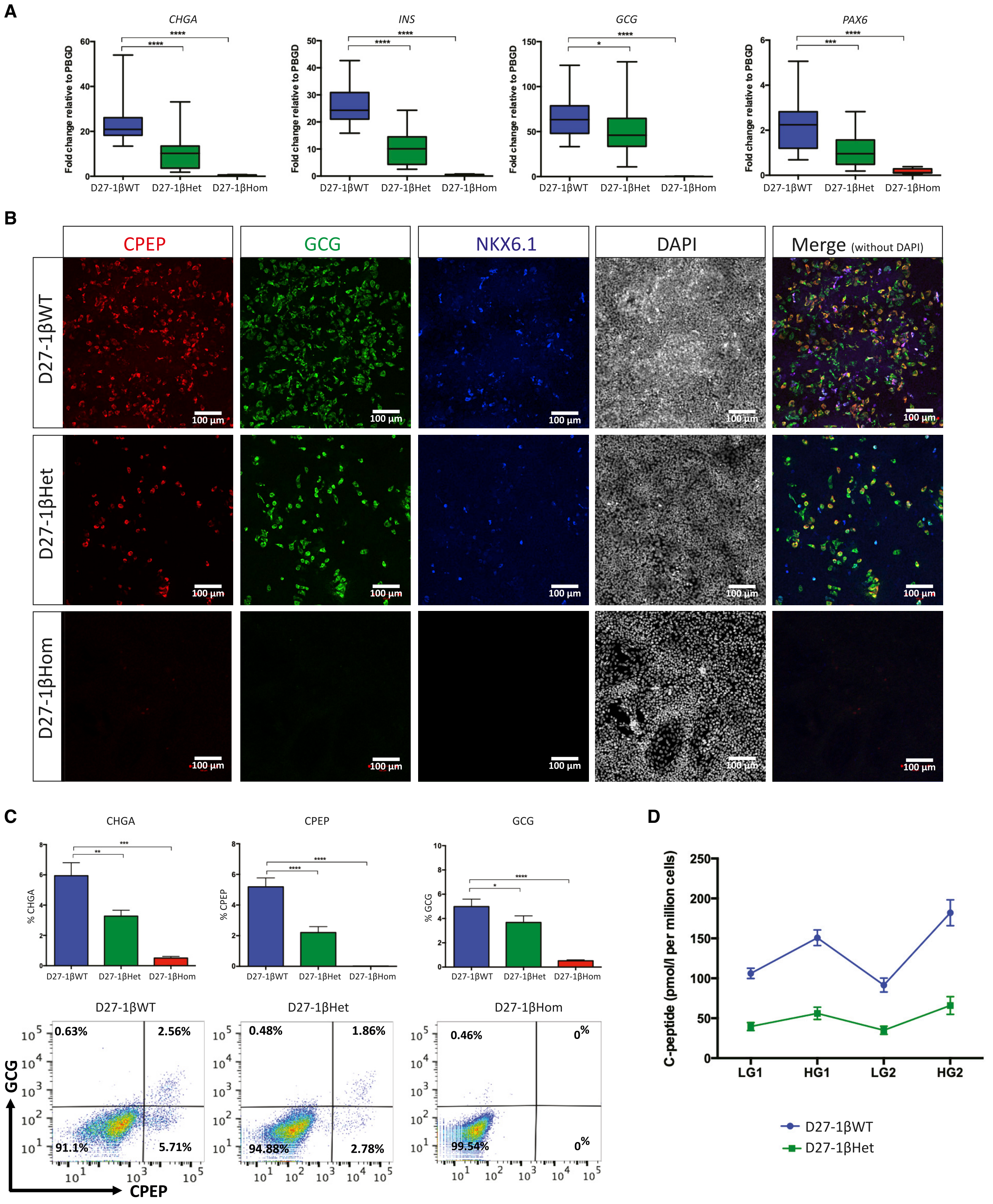

(legend on next page)

PDX1$^+$/NKX6.1$^+$ pancreatic progenitor cells. Other pancreatic progenitor markers, such as *SOX9*, *PTF1A*, and *HNF6/ONECUT1*, also showed statistically significantly reduced expression levels between D13-1βHet and D13-1βWT cells (Figure 2D). These data demonstrate that HNF1B is essential for the efficient formation of posterior foregut, while a decrease in its expression affects production of pancreatic cells.

HNF1B haploinsufficiency impairs the formation but not the functionality of β-like cells

To investigate the functional consequences of HNF1B haploinsufficiency, we further differentiated cells into EPs and then hormonal/β-like cells. As expected from our previous findings, 1βHom mutant lines failed to form EPs or hormonal cells (Figures 3 and S3). The expression of key EP cell markers such as *NEUROD1* and *NEUROG3* was completely abolished in D16-1βHom cells, while *GLIS3* expression was significantly reduced (Figure S3A). In contrast, *NEUROG3* expression was unaffected in D16-1βHet cells, while *NEUROD1* and *GLIS3* were expressed at lower levels compared with D16-1βWT cells (Figure S3A). By the final stage of the differentiation protocol, D27-1βHet cells showed reduced expression of several endocrine markers important for β cell function (Figures 3A and 3B). Interestingly, 1βHet hiPSCs were still able to form CPEP$^+$ hormonal cells, but the percentage was greatly reduced compared with 1βWT cells (2%–3% versus 5%–6%, Figure 3C). Notably, approximately 60%–70% of CPEP$^+$ cells were monohormonal for both D27-1βWT and D27-1βHet cells. However, the D27-1βHet mutant clones had fewer CPEP$^+$ cells co-expressing NKX6.1 (Figure 3B), which is known to play important roles in maintaining adult β cell function (Taylor et al., 2013).

Functional assays on hiPSC-derived β-like cells showed that D27-1βHet cells exhibited reduced glucose-stimulated insulin secretion (GSIS) compared with D27-1βWT cells (Figure 3D). The total concentration of C-peptide secreted was reduced, but not the ratio of C-peptide secreted in high glucose (22.5 mM) to low glucose (2.25 mM), after correcting for the reduced number of CPEP$^+$ β-like cells in 1βHet mutant cells (ratio 1.99 versus 1.91, p = 0.41). This was seen for both FSPS13.B and Eipl_1 hiPSCs. Eipl_1 iPSCs produced reduced numbers of CPEP$^+$ cells and GSIS compared with FSPS13.B iPSCs (data not shown). These data confirm that the absence of HNF1B entirely blocks pancreatic development. A decrease in HNF1B expression, on the other hand, appears to affect the production of pancreatic progenitor cells without entirely inhibiting their capacity to differentiate into insulin-secreting β-like cells.

HNF1B activates key genes at the foregut progenitor and posterior foregut stages to allow specification of pancreatic progenitor cells

To understand the molecular mechanisms underlying how the loss of function of one or both alleles of HNF1B impairs pancreatic progenitor cell development, we used bulk RNA-seq to profile the transcriptomes of 1βWT, 1βHet, and 1βHom hiPSC-derived progenitors at the FP stage (day 6) when HNF1B starts to be significantly expressed, and at posterior foregut (day 8), MPC (day 13), EP (day 16), and hormonal cell/β-like cell (day 27) stages. RNA-seq was profiled from triplicate differentiation experiments for each cell genotype and differentiation stage (Table S2). As expected, the gene expression pattern of key pancreatic differentiation markers was consistent with the results obtained by qRT-PCR (Figure S4).

Principal-component analysis separated 1βWT, 1βHet, and 1βHom cells by day of differentiation in the main component (with PC1 explaining 53% of the variance, Figure S5A). The second principal component explained 20% of the variance and separated cells by genotype. As expected, 1βWT and 1βHet cell lines were transcriptionally more similar to each other than to 1βHom cells. Calculation of the sample-to-sample distance matrix for all samples and replicates followed by unsupervised hierarchical clustering grouped together the sample replicates by day and then by

Figure 3. HNF1B haploinsufficiency impairs β-like cell differentiation

(A) Expression of *CHGA*, *INS*, *GCG*, and *PAX6* in D27-1βWT, D27-1βHet, and D27-1βHom cells. The mRNA levels were measured by qRT-PCR and normalized to the housekeeping gene *PBGD*. Data were pooled from n = 5 independent experiments for each of the eight FSPS13.B clones and n = 3 independent experiments for each of the eight Eipl_1 clones, with clone identities as per Figure S2C. Student's t test with two-tailed distribution was used for statistical analysis. All data are presented as the mean ± SEM unless otherwise indicated. *p < 0.05, **p < 0.01, ***p < 0.001, and ****p < 0.0001.

(B) Representative immunofluorescence images showing CPEP, GCG, and NKX6.1 co-staining in D27-1βWT, D27-1βHet, and D27-1βHom cells.

(C) Percentage of cells expressing CHGA, CPEP, and GCG and representative FACS dot plots of cells derived from 1βWT, 1βHet, and 1βHom hiPSCs at day 27 of the differentiation protocol stained for CPEP and GCG. Replicates and statistics are as indicated for (A).

(D) C-peptide secretion from β-like cells derived from HNF1B$^{+/+}$ (D27-1βWT) and HNF1B$^{+/-}$ (in D27-1βHet) hiPSCs. Cells were incubated in high-glucose (22.5 mmol/L) and low-glucose (2.25 mmol/L) culture medium for two rounds of stimulations. Replicates were as indicated for (A). Error bars indicate SEM.

1βHom genotype, but was not able to clearly cluster 1βWT and 1βHet cells (Figure S5B). These analyses therefore confirm that the absence of HNF1B has a transcriptional impact on pancreas specification from hPSCs at the foregut stage and on, while heterozygous knockouts have a limited but still detectable effect.

A more detailed analysis revealed the sets of differentially expressed genes from pairwise comparisons among samples derived from 1βHet and 1βHom cells, compared with their 1βWT counterparts. Consistent with the global analysis described above, we detected a much larger fraction of genes differentially expressed in 1βHom cells than in 1βHet cells (Table S3, Figures 4A–4D and S4B). Interestingly, the early effect of a low HNF1B dosage (in D6-1βHet and D8-1βHet cells) was mainly the downregulation of tens of genes, with almost no genes upregulated in these samples (Table S3). Most of the downregulated genes in 1βHet-derived progenitors at these stages could not be associated with known definitive endoderm/FP functions. We note, however, the consistent downregulation of the HNF1A antisense long non-coding RNA (lncRNA *HNF1A-AS1*) in all 1βHet and 1βHom samples from all stages (Figure 4E). In particular, *HNF1A-AS1* expression was downregulated, but not abolished, in samples derived from 1βHet cells, without impairing *HNF1A* expression. In sharp contrast, *HNF1A-AS1* expression was completely abolished in all samples derived from 1βHom cells, and its associated gene, coding for the transcription factor HNF1A, was not expressed in these cells (Figure 4E). Notably, *HNF1A-AS1* presents active chromatin marks at its promoter and nearby regulatory regions (H3K27ac and H3K4me1) in *in vitro* MPCs (data from our previous study, Cebola et al., 2015) and strong HNF1B and FOXA2 chromatin immunoprecipitation sequencing (ChIP-seq) binding sites at its promoter (Figure 4F). Thus, *HNF1A-AS1* could be one of the earliest HNF1B directly regulated gene targets. We also detect TEAD1 and FOXA2 binding at an active enhancer region upstream of the HNF1A promoter, potentially involved in the regulation of *HNF1A* and/or *HNF1A-AS1*. Other genes downregulated in 1βHom-derived progenitors included well-known pancreatic regulators such as *HNF4A*, *FGFR4*, *HHEX*, *SFRP5*, and *PDX1* (Figures 4A–4D, Table S3). These results suggest that the effect of early HNF1B activation, at days 6 and 8, is mainly the upregulation of a few key specific genes. The numbers of up- and downregulated genes then increase in D13-1βHet, D16-1βHet, and D27-1βHet cells. Taken together, these findings are consistent with an activator role for HNF1B at the FP and posterior foregut stages.

To gain further insights into the molecular pathways controlled by HNF1B in the initial stages of pancreas specification, we functionally annotated the sets of up- and downregulated genes in 1βHet- and 1βHom-derived cells, compared with 1βWT cells. Given that pancreas specification is abolished in 1βHom cells, we characterized in further detail the pathways enriched in these cells from day 6 to day 13. Biological pathway analysis of downregulated genes revealed significant enrichment for terms associated with lipid and retinoic acid metabolism (D6-1βHom and D8-1βHom) and endocrine pancreas development (D13-1βHom, Table S4, Figure 4G). In contrast, upregulated genes were enriched in annotations associated with alternative developmental pathways, notably heart, kidney, and nervous system development. Interestingly, upregulated genes in D13-1βHom cells were enriched in "negative regulation of cell proliferation" (Table S4, Figure 4G), suggesting that a low dosage of HNF1B at this stage could be associated with impairment of MPC population expansion. Despite not finding enrichment for this category in the D13-1βWT versus D13-1βHet cell comparison, we note that the expression of this gene set presents the same downregulation trend as in D13-1βHom samples, although at milder fold changes (Figure 4G). Considered together, these observations show that HNF1B plays a central role in the specification of the foregut toward the pancreatic lineages by controlling key master regulators, while its full expression could be necessary for proliferation of MPCs.

HNF1B haploinsufficiency impairs cell proliferation in foregut and pancreatic multipotent progenitor cells

We next interrogated whether the negative regulation of cell proliferation in D13 progenitor cells could be one of the effects of the lack or low dosages of HNF1B early during pancreas development. Indeed, the numbers of cells harvested at days 6 and 13 were significantly lower in 1βHom cells compared with 1βWT cells (Figure 5A), starting at the FP stage (1.46×10^6 cells versus 2.2×10^6 versus 2.37×10^6, $p < 0.05$, $p = 0.24$). The difference was larger, for both 1βHom and 1βHet cells, at the MPC stage (3.78×10^6 versus 5.38×10^6 versus 7.33×10^6, $p < 0.05$, $p < 0.05$). To explain the reduction in cell number, we compared the rate of apoptosis and cell proliferation during the differentiation of 1βWT, 1βHet, and 1βHom cells. Apoptosis assays (using propidium iodide and Annexin V staining) performed at the FP and MPC stages showed no significant difference in the number of cells in early or late apoptosis between 1βWT, 1βHet, and 1βHom cell lines (Figure 5B). The number of proliferating cells was next determined using 5-ethynyl-2′-deoxyuridine (EdU) incorporation. A significant reduction in cell proliferation was seen in D6-1βHet and D6-1βHom cells at the FP stage compared with their 1βWT counterparts (Figure 5C). There was a significant decrease in the number of cells in S phase and a corresponding increase in the percentage of cells at G1 and G2/M phase, as non-proliferating cells accumulated at these stages. At the MPC stage, there was a significant decrease in the number of cells in S phase in both D13-1βHet and D13-1βHom

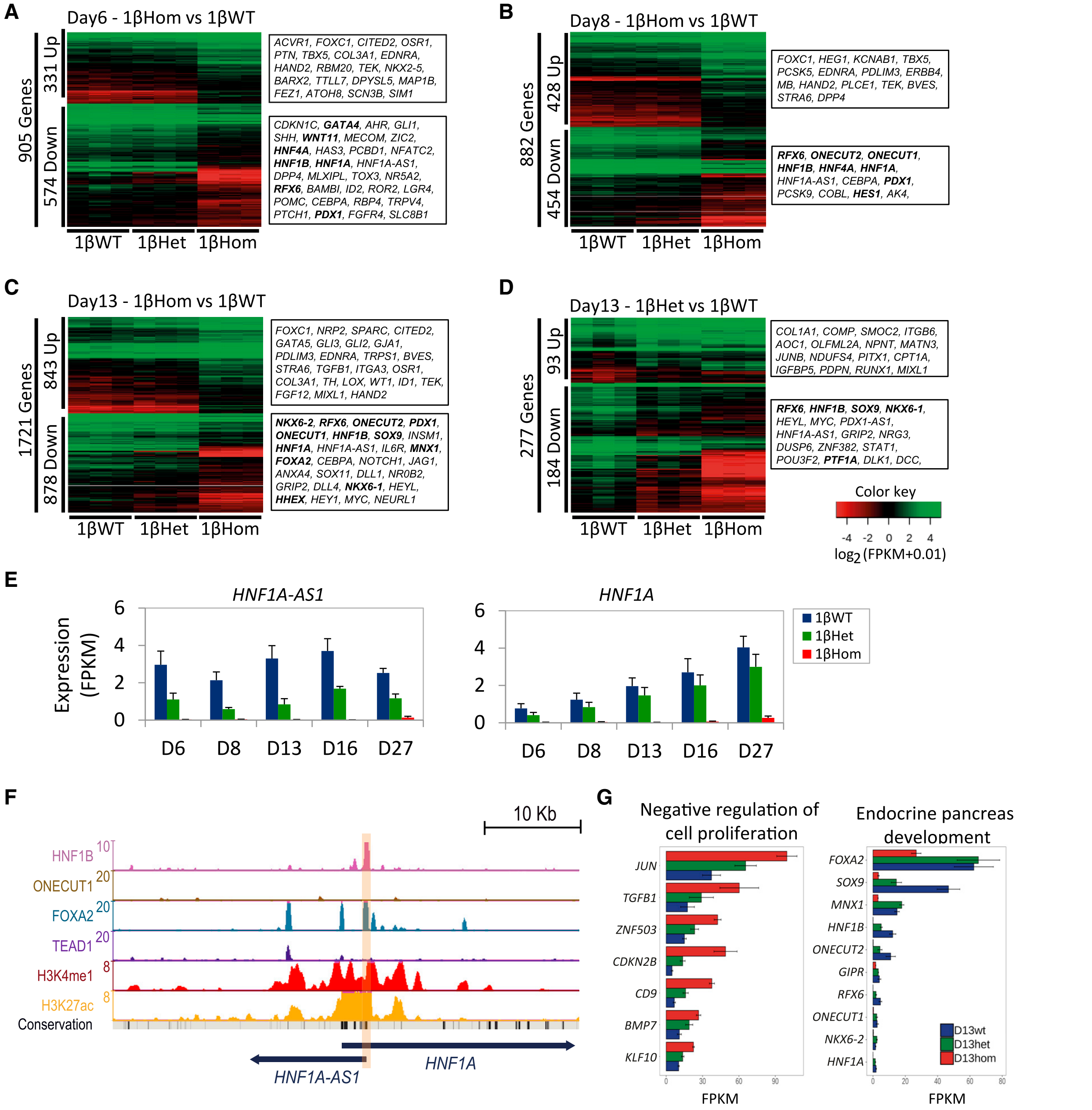

Figure 4. HNF1B activates key genes at the foregut progenitor and posterior foregut stages to allow specification of pancreatic progenitor cells

(A–C) Expression of differentially regulated genes between 1βWT and 1βHom for the three genotypes at day 6 (A), day 8 (B), and day 13 (C) of the differentiation protocol.

(D) Expression of differentially regulated genes between 1βWT and 1βHet for the three genotypes at day 13 of the differentiation protocol. The bolded genes on the right are known to be important for pancreas development.

(E) Expression of *HNF1A-AS1* and *HNF1A* for all genotypes and differentiation stages of the *in vitro* protocol.

(F) UCSC genome browser snapshot of the *HNF1A* genomic locus. ChIP-seq was used to locate binding sites of HNF1B, ONECUT1, FOXA2, and TEAD1 in MPCs. ChIP-seq for H3K4me1 and H3K27ac histone modifications denotes the epigenomic printing of active enhancers. HNF1B binding at the *HNF1A-AS1* promoter is highlighted in light orange.

(G) Expression levels of a selection of genes from the gene ontology enriched terms “endocrine pancreas development” and “negative regulation of cell proliferation” across the three genotypes.

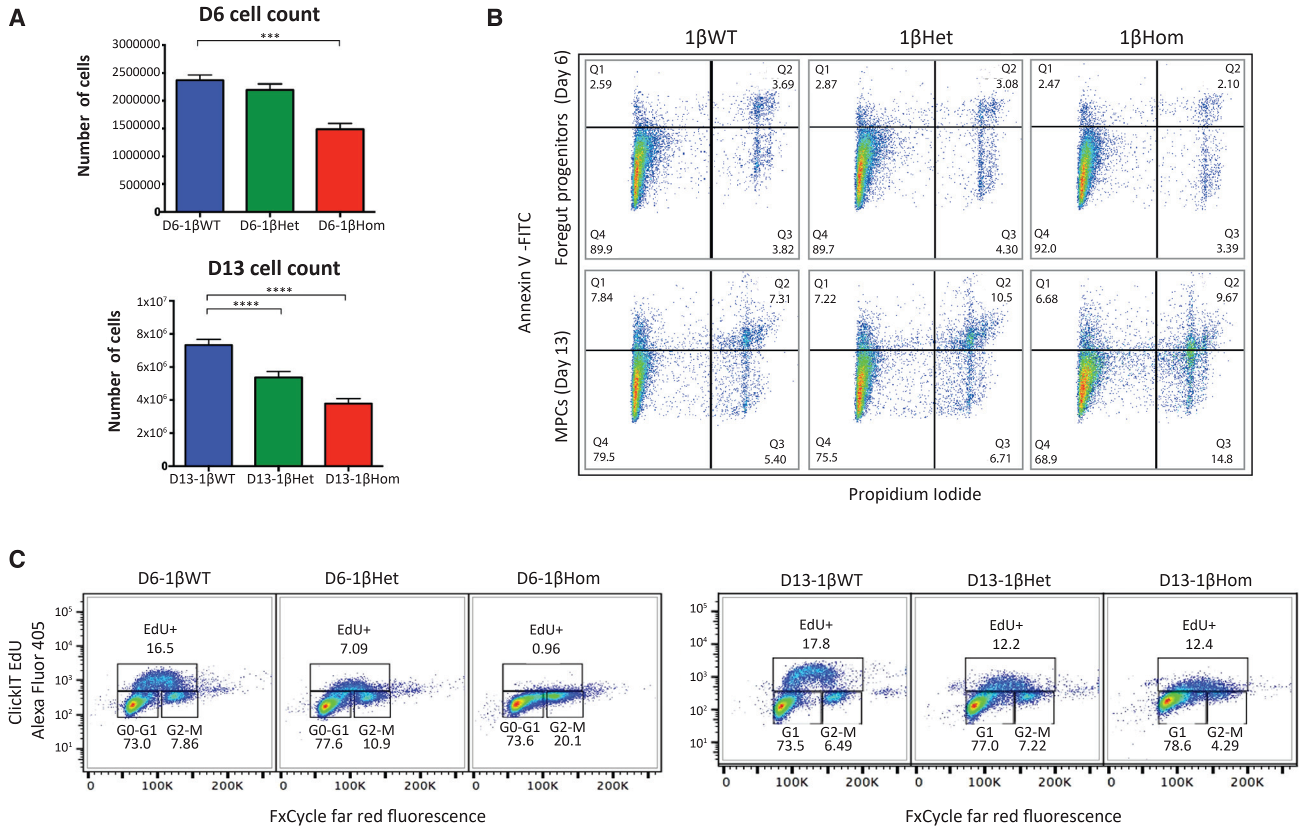

Figure 5. HNF1B haploinsufficiency impairs cell proliferation in foregut and pancreatic multipotent progenitor cells

(A) Cell count determined by the number of cells harvested from one well of a 12-well plate of 1βWT, 1βHet, and 1βHom cells taken at the foregut progenitor stage (D6) and pancreatic progenitor stage (D13) of differentiation. n = 3 independent experiments derived from FSPS13.B clones. Student's t test with two-tailed distribution was used for statistical analysis. Data are presented as the mean ± SEM. ***$p < 0.001$ and ****$p < 0.0001$.

(B) Apoptosis assay in 1βWT, 1βHet, and 1βHom cells taken at the foregut progenitor stage (D6) and pancreatic progenitor stage (D13) of differentiation.

(C) EdU staining showing percentage of cells in G1, S, and G2M phase for plates of 1βWT, 1βHet, and 1βHom cells taken at the foregut progenitor stage (D6) and pancreatic progenitor stage (D13) of differentiation. n = 3 independent experiments derived from FSPS13.B clones.

compared with D13-1βWT cells. There was no consistent decrease in proliferation among the D13-1βHet and D13-1βHom cells, suggesting that by this stage 1βHom cells have a different identity and respond differently to external stimuli. Taken together, these results suggest that the loss of one functional *HNF1B* allele results in decreased cell proliferation, which impairs the production of pancreatic progenitor cells from foregut cells. This could potentially explain the reduced production of $CPEP^+$ β-like cells later on.

Potential alternative paths for production of endocrine cells from *in vitro*-derived pancreatic progenitor cells at day 13

The results from the previous sections suggest that D13-1βHet cells present a broadly similar gene expression profile (Figures S5A and S5B). Yet, they have impaired cell proliferation and impaired β-like cell production, which are, however, functionally similar to those derived from 1βWT progenitors. To gain a deeper understanding of the mechanisms that allow specification of the pancreatic endocrine lineage in cells with low HNF1B dosages, we performed single-cell RNA-seq (scRNA-seq) in 1βWT and 1βHet cells from day 13, a stage at which the HNF1β dosage on the number of differentially regulated genes became more evident.

An unsupervised graph-based clustering allowed identification of four cell clusters, each containing both 1βHet- and 1βWT-derived cells (Figures 6A, 6B, and S6C). Analysis of the combined expression profiles for these clusters matched highly proliferative MPCs ("early

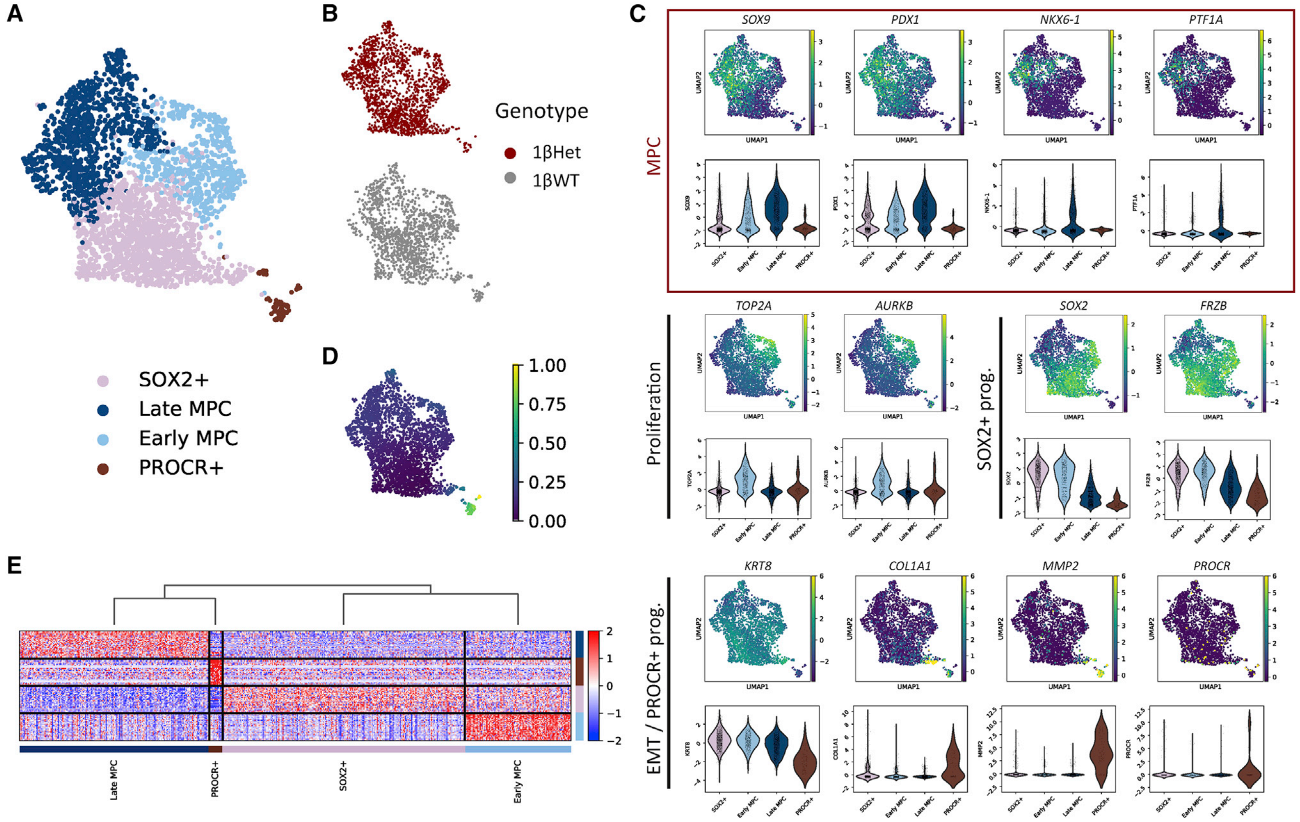

Figure 6. scRNA-seq analysis reveals cell populations derived *in vitro* from 1βWT and 1βHet hiPSCs

(A) Uniform manifold approximation and projection (UMAP) plot of 3,216 single-cell transcriptomes profiled from the day 13 differentiation time point (1βWT and 1βHet samples). Colors in the UMAP on the right highlight clustering into four main cell subtypes that were matched with their closest *in vivo* progenitor cell type as described in the main text.

(B) UMAP plots showing the distribution of clustered cells colored according to the genotype.

(C) Feature and violin plots showing expression of selected progenitor cell genes in human *in vitro*-derived pancreatic cells clustered as in (A). The red rectangle highlights the MPC markers.

(D) Pseudotime order of *in vitro*-derived progenitor cells shown in the UMAP plot in (A).

(E) Heatmap of the top 50 enriched genes for each cluster. Each column represents a single cell and each row represents one signature gene. The colors ranging from blue to red indicate low to high relative gene expression levels. The dendrogram on top of the heatmap indicates that late MPC and PROCR$^+$ cells have closer transcriptional profiles according to these markers.

MPC”) and less proliferative MPCs (“late MPC”), as recently described for *in vitro* human pancreatic differentiation protocols at a similar differentiation stage (Veres et al., 2019). These cells showed combined expression of MPC markers *PDX1*, *SOX9*, *PTF1A*, *DLK1*, and *NKX6-1*, with late MPC having the highest expression for all these markers (Figures 6C and S6A). Late MPCs also expressed higher levels of *CPA2*. We also detected a cluster of progenitor cells with high *SOX2* and *FRZB* expression (“SOX2$^+$,” Figures 6A and 6C, Table S5). A similar cluster was also described in a subset of pancreatic progenitor cells derived *in vitro* (Veres et al., 2019) and ascribed to non-endocrine committed progenitors. Notably, SOX2 has been detected early during pancreas specification in the pre-pancreatic gut region and reported to be soon excluded from pancreatic buds (Wilson et al., 2005). These cells also expressed high levels of *SOX21*, which has been previously detected in the mouse developing pancreas (Wilson et al., 2005). Importantly, *SOX2* and *SOX21* expression levels were rapidly downregulated in the other cell clusters, thus suggesting that these cells represent pre-pancreatic gut progenitor cells. As well, SOX2$^+$ progenitor cells expressed moderate levels of *PDX1* and *SOX9*, while *NKX6-1* and *PTF1A* were barely detected (Figure 6C). The expression of all these markers gradually increased from SOX2$^+$ progenitors to early MPC, having the highest levels in late MPC (Figure 6C).

We additionally detected a fourth cluster that presented co-expression of epithelial and mesenchymal markers, including *KRT8*, *COL1A1*, and *MMP2* (Figures 6C, S6A, and S6B), indicative of the epithelial-to-mesenchymal transition (EMT). Interestingly, this cluster also expressed several markers matching a recently reported Procr^{+} progenitor population present in adult mouse pancreatic islets (Wang et al., 2020), including *PROCR*, *SPARC*, and *IGFBP5* (Figure S6A), in addition to co-expressing the EMT markers mentioned above and being *NEUROG3*$^{-}$ (not detected in any of the cell clusters of our scRNA-seq dataset). Notably, Procr^{+} progenitors from adult mouse islets were reported to give rise to all endocrine cells without passing through an Ngn3^{+} cell stage. A pseudotime analysis further supported the progenitor match for our clusters (Figure 6D), showing a differentiation cluster order from SOX2^{+} progenitors either toward PROCR^{+} progenitors or into early MPC and late MPC. Notably, a clustering analysis performed using the top 50 markers for each cell cluster revealed that SOX2^{+} and early MPCs have closer transcriptional profiles, consistent with their early progenitor stage (Figure 6E). Conversely, late MPCs and PROCR^{+} cells were first clustered together, in agreement with the advanced differentiated stage of these cells.

HNF1B haploinsufficiency impairs the early stage pancreatic developmental program by altering expression of key non-canonical Wnt and Hippo signaling pathway components

We next sought to identify the transcriptional effects of low HNF1B dosages in each cell cluster. The proportion of 1βWT and 1βHet cells (after normalizing by the total number of cells per genotype) did not change for SOX2^{+} progenitors, but low HNF1B dosages switched the balance between early MPC, late MPC, and PROCR^{+} progenitors (Figure 7A). In other words, the number of late MPCs in D13-1βHet samples was higher than in their 1βWT counterparts, and this increase appeared to take place mainly at the expense of the early MPC population. This finding is in agreement with the decreased proliferation in bulk D13-1βHet cell cultures (Figure 5), since early MPCs are highly proliferative progenitors, as evidenced by the increased expression of the proliferation markers *TOP2A* and *AURKB* (Figure S6A). These results suggest that HNF1B plays an important role in allowing the proliferative early MPC stage.

Looking at the genes that are differentially expressed in 1βHet for each cluster revealed significant downregulation of some known and potentially novel pancreatic regulators (Figure S7A, Table S6). In addition to *HNF1B* itself, these included *SOX11* (in the SOX2^{+} progenitor, early MPC and late MPC clusters); *SOX4*, *TEAD1*, *GATA6*, and *HMGA2* (in the SOX2^{+} progenitor cluster); and *ONECUT2* in the early MPC cluster (Cebola et al., 2015; Sarkar et al., 2008; Wilson et al., 2005; Yu et al., 2019). Interestingly, several genes upregulated in 1βHet in the SOX2^{+} progenitor, early MPC, and late MPC cluster cells coded for pancreatic exocrine enzymes, including serine proteases *PRSS1* and *PRSS2* and carboxypeptidase *CPB1*. However, most of the differentially expressed genes in the presence of low HNF1B dosages have unannotated functions in pancreas development.

Given that 1βHet samples had a larger number of late MPCs at the expense of early MPCs, we further inquired the relevance of genes differentially regulated in these clusters in the context of pancreas development. For this purpose, we filtered them by its association with our previously reported set of 9,669 MPC enhancers (Cebola et al., 2015). As expected, a large fraction was associated with MPC enhancers (45.1%/40.9% of downregulated genes and 50%/39.6% of upregulated genes in 1βHet early MPC/late MPC, respectively), and most of these genes were also associated with nearby TEAD1 binding sites (Table S7). Interestingly, almost all genes associated with HNF1B-bound MPC enhancers were also TEAD1 targets (Figure S7B). These included the transcription factor *SOX11* and the transmembrane receptor *ROBO2* (downregulated in 1βHet early MPC and late MPC clusters, Figures 7B–7D and S7C). Notably, *Robo1* and *Robo2* have been recently reported to play a key role during pancreas development by controlling expression of Tead transcription factors and its downstream transcriptional activity, ultimately regulating the expansion of the pancreatic progenitor cell pool (Escot et al., 2018). We thus interrogated the expression of *ROBO1*, *ROBO2*, all four TEAD transcription factors, *YAP1*, *TAZ*, and the well-established TEAD target gene *CTGF*. Indeed, *ROBO2* downregulation in 1βHet early MPCs appears to be compensated for by increased *ROBO1* expression in these cells (Figure 7C). As well, while *TEAD1*, *TEAD2*, *TEAD3*, *YAP1*, and *CTGF* are expressed at lower levels in 1βHet early MPCs, *TEAD4* and *TAZ* appear to be slightly upregulated. Thus, *ROBO2* downregulation in 1βHet early MPCs could affect Hippo signaling in these cells, an adverse effect that appears to be partially compensated for by *ROBO1*, *TEAD4*, and *TAZ* upregulation. Similar *ROBO2*/*ROBO1*, *TEAD*, and *YAP* regulatory events were observed in cells from SOX2^{+} and late MPC clusters (Figure S7D). Notably, the epigenomic loci containing the *ROBO2* and *ROBO1* genes reveals several HNF1B- and TEAD1-bound MPC enhancers at the promoter and upstream regulatory regions of *ROBO2*, with regulatory regions near the *ROBO1* gene located downstream of its promoter (Figure 7D). Additional TEAD1 targets downregulated in 1βHet cells included *SFRP5* in early MPCs and *FZD5* in late MPCs (Figures 7B and S7C, Table S7), known regulators of the non-canonical Wnt and/or

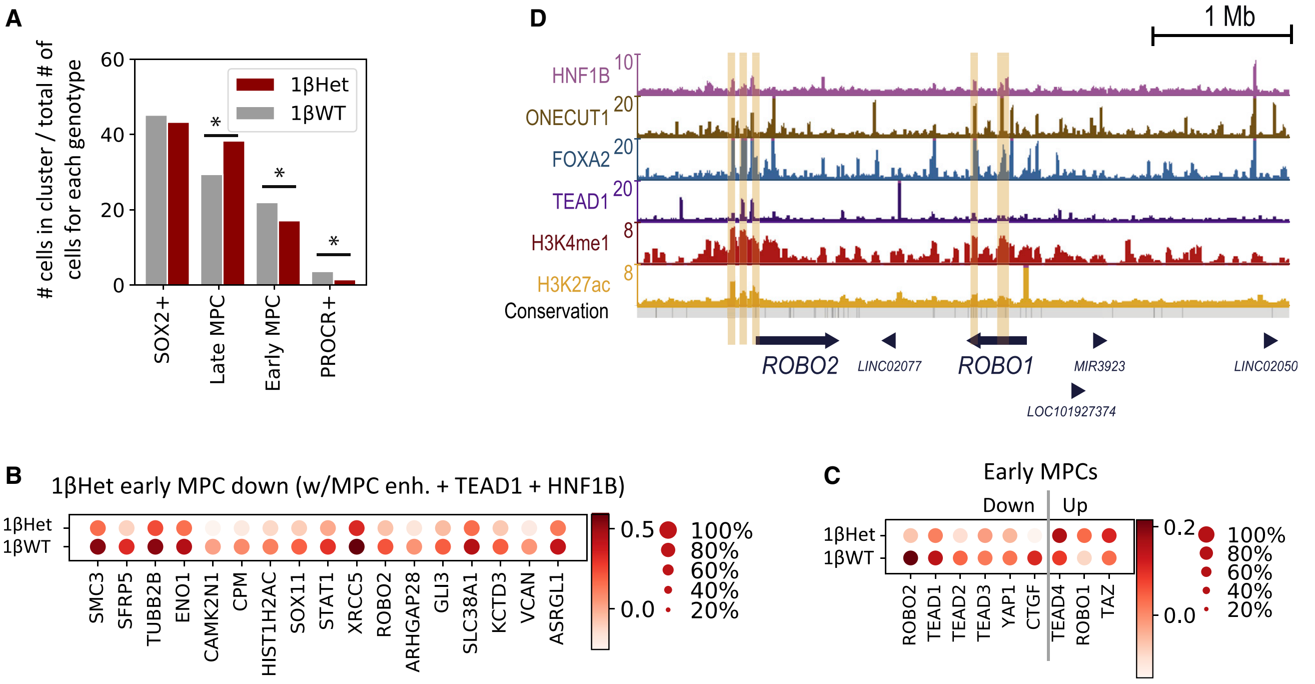

Figure 7. HNF1B haploinsufficiency impairs the early stage pancreatic developmental program by altering expression of key non-canonical Wnt and Hippo signaling pathway components

(A) The distribution of clustered cell types by genotype. The numbers of clustered 1βWT and 1βHet cells were normalized independently by genotype; the sum of all red bars accounts for 100% of 1βHet cells and that of the gray bars for 100% of 1βWT cells.*$p < 0.001$.

(B) Dot plot showing the expression of genes significantly downregulated in 1βWT and 1βHet early MPCs. Genes were filtered by the association with at least one MPC enhancer (as previously defined in Cebola et al., 2015) that presents both TEAD1 and HNF1B ChIP-seq enrichment. Color intensity indicates mean expression (normalized) in a cluster, dot size indicates the proportion of cells in a cluster expressing the gene.

(C) Dot plot showing expression of *ROBO1*, *ROBO2*, selected Hippo pathway components, and its known target *CTGF* in 1βWT and 1βHet early MPCs. Color intensity indicates mean expression (normalized) in a cluster, dot size indicates the proportion of cells in a cluster expressing the gene.

(D) UCSC genome browser snapshot of the *ROBO1* and *ROBO2* genomic locus. ChIP-seq was used to locate binding sites of HNF1B, ONECUT1, FOXA2, and TEAD1 in MPCs (data from Cebola et al., 2015). ChIP-seq for H3K4me1 and H3K27ac histone modifications denotes the epigenomic printing of active enhancers. MPC enhancers enriched in HNF1B signal in this locus are highlighted in light orange.

Hippo signaling pathways (Rodriguez-Seguel et al., 2013; Sharon et al., 2019), thus potentially accounting for the reduced cell proliferation observed in these cells. To conclude, these analyses reveal that HNF1B haploinsufficiency could result in the defective regulation of effectors of the Wnt and Hippo pathways, which in turn could decrease proliferation of early MPCs.

DISCUSSION

We describe here the use of a well-controlled hiPSC pancreatic differentiation model to elucidate the molecular mechanisms underlying HNF1B-associated diabetes and pancreatic hypoplasia. Our results reveal that a lack of HNF1B blocks specification of pancreatic fate from FPs. Indeed, upregulated genes in D6-1βHom and D8-1βHom cells are enriched in annotations associated with heart, kidney, and nervous system development, suggesting that absence of HNF1B affects foregut patterning by allowing cells to adopt alternative fates. These results suggest that homozygous loss of HNF1B protein expression in the human embryo is likely to be lethal due to a primary defect in gut tube/foregut formation. Conversely, HNF1B haploinsufficiency allows differentiation of MPCs and ultimately β-like cells. The β-like cells differentiated from 1βHet cells are functional, to the same degree as their 1βWT-derived counterparts, but the former are produced in a smaller amount.

In sharp contrast with a previous study reporting the use of MODY5 patient-derived hiPSCs for differentiation of pancreatic progenitors and β-like cells (Teo et al., 2016), here we detect downregulation of *PDX1* and other important pancreatic regulators, including *PTF1A*, *NKX6.1*, *SOX9*, and *RFX6*, in D13-1βHet cells (MPC stage). The

main difference among the studies resides in the strategy used to model HNF1B-associated diabetes. Teo and colleagues used hiPSCs derived from MODY5 patients carrying an S148L mutation that potentially affects the DNA binding efficiency of HNF1B. Thus, binding of mutated HNF1B to its genomic regulatory regions could be less efficient, but not abolished. In this context, an increase in gene expression could be a compensatory mechanism to enhance HNF1B activity. As well, these researchers used non-isogenic hiPSCs derived from another family member and a non-related individual as control cell lines. Thus, the results could be influenced by additional contributions from the genetic background, as discussed in more detail in a recent review (Burgos et al., 2021). On the other hand, for the studies presented here, we used a better controlled cell model in which we completely disrupted *HNF1B* expression from one or both alleles, and used isogenic non-mutated hiPSCs as controls. Our strategy more accurately reflects HNF1B haploinsufficiency, since we see that *HNF1B* expression is reduced by half in D6-1βHet and D13-1βHet cells. Notably, this model might more closely recapitulate the mechanisms underlying HNF1B-associated diabetes in MODY5 patients with nonsense or frameshift mutations, in which the HNF1B protein function is more severely compromised (Edghill et al., 2006a).

One of the earliest transcriptional events we noticed in samples derived from both 1βHom and 1βHet cells is the failure to upregulate *HNF1A-AS1*, whose promoter is bound by HNF1B in WT *in vitro* MPCs. However, while progenitors derived from 1βHom cells have impaired expression of both *HNF1A-AS1* and *HNF1A*, samples derived from their 1βHet counterparts express this lncRNA at considerably lower levels and *HNF1A* close to WT levels. This could be one of the main differences allowing for the early transcriptional divergence among progenitors derived from 1βHom and 1βHet cells, as soon as from the foregut stage (day 6). Interestingly, a very recent work by Ferrer and colleagues shows that *HNF1A-AS1* (renamed as *HASTER* in their work) maintains the expression of *HNF1A* at physiological cell-specific levels through positive and negative feedback loops (Beucher et al., 2021). In the model proposed, increased *HNF1A-AS1* expression (and thus activation of its promoter) downregulates *HNF1A* mRNA expression levels by "sequestering" binding of an *HNF1A* intronic enhancer from the *HNF1A* promoter. Notably, although forced high protein levels of either HNF1A or HNF1B increase *HNF1A-AS1* expression in the EndoC-βH3 cell line, only elevated HNF1A protein levels are able to downregulate the endogenous *HNF1A* mRNA expression (Beucher et al., 2021). This is consistent with our findings because, although low levels of HNF1B in D8-1βHet and D13-1βHet cells result in an important decrease in *HNF1A-AS1* expression, we do not see a concomitant increase in *HNF1A* mRNA levels. It should be noted, however, that embryonic and adult levels of *HNF1A* could be driven by different enhancers. Indeed, while Ferrer and colleagues focus on an enhancer located in an intron of *HNF1A* as the regulatory element "sequestered" by the *HNF1A-AS1* promoter, in our work we detect a TEAD1- and FOXA2-bound regulatory region located upstream of the *HNF1A* promoter. A more detailed study of the interplay between *HNF1A-AS1* and *HNF1A* expression in this model of HNF1B-associated diabetes is an exciting area for future research.

We further report here that HNF1B haploinsufficiency impairs cell proliferation in foregut and MPCs *in vitro*. The quantity of MPCs produced is likely to be influenced by other transcription factors and environmental cues, thereby explaining the variability in the penetrance of HNF1B mutations in human. Importantly, this decrease in MPC number originates from a defect not only in cell proliferation, but also in specification. Indeed, a dose-sensitive effect of HNF1B loss was observed, since heterozygous knockout of HNF1B in hiPSCs resulted in significant impairment, but not complete loss, of pancreatic progenitor cell development. While $PDX1^{+}$ cells can be produced with similar efficiency from 1βWT and 1βHet at day 13 (MPC stage), there is a significant reduction of $PDX1^{+}$/$NKX6.1^{+}$ MPCs. Single-cell transcriptomic analyses reveal that HNF1B haploinsufficiency switches the balance of progenitor cell populations derived *in vitro*. Thus, a low dosage of HNF1B in progenitor cells alters the early stage pancreatic specification program, downregulating the expression of several genes with known or suspected roles in pancreas development. Our analyses suggest that, on one hand, this could be due to impaired early MPC specification from gut progenitor cells. At this time point, 1βHet $SOX2^{+}$ progenitors fail to upregulate *SOX11*, *SOX4*, *GATA6*, and *HMGA2*, among other genes potentially involved in the early specification of pancreatic MPCs. Later on, at the MPC stage, 1βHet early MPCs express lower levels of key pancreatic developmental genes, including *SOX11*, *ROBO2*, and additional TEAD1 target genes whose function could be associated with MPC self-renewal (Cebola et al., 2015; Escot et al., 2018; Willmann et al., 2016; Yu et al., 2019). Interestingly, it has been recently reported in the mouse that *Robo1* and *Robo2* are required to stabilize the pancreatic cell identity after fate induction and, later on, for expansion of the pancreatic progenitor cell pool (Escot et al., 2018). Robo receptors can control the expression of Tead transcription factors and its downstream transcriptional activity. These findings are consistent with our previous report describing a key role for TEAD and YAP in controlling the gene expression program in MPCs (Cebola et al., 2015), and are in agreement with the results

presented here. Taken together, these observations allow us to hypothesize that downregulation of some TEAD target genes in 1βHet early MPCs, potentially mediated by impaired *ROBO2* expression, might enhance cell differentiation at the expense of MPC pool self-renewal. We note that *ROBO1* expression could be partially compensating for this effect in early MPCs, thus allowing a plausible explanation for the less efficient, but not truncated, production of β cells from 1βHet hiPSCs.

Other factors that could underlie the adverse effects of HNF1B haploinsufficiency on pancreas development include *SOX4* and *SOX11*. Of these, *SOX11* was robustly downregulated in all cell clusters derived from D13-1βHet samples. *SOX4*, on the other hand, is downregulated in D13-1βHet SOX2$^+$ cells. Both factors have been previously described in the context of pancreas development. Sox4 knockout in mice results in *Sox11*, *Neurog3*, and *Neurod1* upregulation in the E12.5 pancreas (Wilson et al., 2005). Sox11 knockout mice present hypoplasia of the pancreas (Sock et al., 2004). Likewise, the reduced *SOX11* expression in D13.1βHet SOX2$^+$ progenitors and early MPCs found in our HNF1B-deficient pancreatic cell differentiation model could at least partially explain the organ hypoplasia found in patients with HNF1B-associated diabetes.

Taken together, our findings show that HNF1B haploinsufficiency could result in pancreas hypoplasia in humans due to an altered production of multipotent progenitors. The downstream molecular mechanisms could involve several gene candidates, including *ROBO2*, *SOX4*, and *SOX11*. Downregulation of this set of genes has the potential to alter the early stage pancreatic specification program, which at this time point involves TEAD and YAP gene target regulation through the Hippo signaling pathway. The modulation of these factors during fetal life by environmental stimuli could compensate in part for the decrease in HNF1B expression, explaining the variable penetrance of HNF1B-associated diabetes. Future studies addressing the functional role of these factors could help to develop new therapies against this disease.

EXPERIMENTAL PROCEDURES

hiPSC generation, characterization, and differentiation

Two hiPSC lines, FSPS13.B and Eipl_1, were used for genome editing and pancreatic differentiation experiments. The hiPSCs were derived from human skin fibroblasts and peripheral blood. Ethics approval was obtained from the National Research Ethics Service Committee East of England, Cambridge East (Ethics Reference 09/h0304/77). Clonal hiPSC mutant lines were generated using the CRISPR-Cas9 technology as described in detail in the supplemental experimental procedures. hiPSCs were cultured and differentiated as previously described (Chia et al., 2019; Cho et al., 2012) with minor modifications as described in the supplemental experimental procedures.

Western blot, immunofluorescence, FACS, qRT-PCR, apoptosis, and cell proliferation assays

Methods for western blot, immunofluorescence, FACS, qRT-PCR, apoptosis, and cell proliferation assays have been described previously (Chia et al., 2019; Cho et al., 2012; Yiangou et al., 2019), and detailed information is provided in the supplemental experimental procedures. Images were taken using a Zeiss LSM 700 confocal microscope (Carl Zeiss, Jena, Germany).

RNA-seq

For the bulk RNA-seq experiments, one HNF1B$^{+/+}$, one HNF1B$^{+/-}$, and one HNF1B$^{-/-}$ (targeted WT) clone from the FSPS13.B hiPSC line were differentiated along the pancreatic lineage. RNA was extracted and sequenced as previously described (Chia et al., 2019). Three independent experiments (biological triplicates generated from FSPS13.B clones) were sequenced for each clone at each stage of differentiation. Bioinformatics analyses were carried out following standard procedures (Cebola et al., 2015; Chia et al., 2019; Conesa et al., 2016).

Single-cell RNA-seq

Single-cell libraries from D13-1βHet and D13-1βWT samples were generated using the Chromium Single Cell 3′ Library & Gel Bead Kit v.2 (PN 120237) from 10× Genomics. Libraries were sequenced on the HiSeq 4000 (Illumina) with 125 bp paired-end sequencing. Analysis of scRNA-seq data included filtering, alignment to the GRCh38 human genome version 28 (Ensembl 92), and unique molecular identifier collapsing performed using the Cell Ranger (v.2.01) pipeline with default mapping arguments (10× Genomics). All further analyses were run with Python 3 using the Scanpy API package (Wolf et al., 2018). Additional details are provided in the supplemental experimental procedures.

Quantification and statistical analysis

For both FSPS13.B and Eipl_1, we used three WT clones (HNF1B$^{+/+}$; one non-targeted WT and two targeted WT clones), three heterozygous clones (HNF1B$^{+/-}$), and two homozygous clones (HNF1B$^{-/-}$; one with a puromycin cassette in both alleles and one with a puromycin cassette in the first allele and an indel in the second allele). The clone identities are shown in Figures S2C and S2E. The data in the main and supplementary figures are pooled from experiments using FSPS13.B and Eipl_1 clones for qPCR, flow cytometry, and ELISA. Quantification data are presented as the mean ± SEM. Data from clonal lines of the same genotype were combined for calculating the significance of the differences between different genotypes. To directly compare two groups, Student's t test with two-tailed distribution was used to test for statistical significance. p values less than 0.05 were considered statistically significant. All statistical analyses were performed using GraphPad Prism 6.0 (GraphPad Software, San Diego, CA, USA) or the R statistical environment.

Data and software availability

The accession number for all raw and processed sequencing data reported in this paper is NCBI Gene Expression Omnibus (https://www.ncbi.nlm.nih.gov/geo/): GSE168071.

SUPPLEMENTAL INFORMATION

Supplemental information can be found online at https://doi.org/10.1016/j.stemcr.2021.07.018.

AUTHOR CONTRIBUTIONS

R.K., K.T., M.C., S.V., and C.Y.C. performed wet lab experiments. R.K. generated RNA-seq biological samples. P.M. and E.O. performed bulk RNA-seq bioinformatic analyses. E.O., D.M., and S.A.R.-S. analyzed scRNA-seq data. S.A.R.-S. performed epigenomic analyses. R.K. and L.V. conceived the initial experiments. R.K., E.O., L.V., and S.A.R.-S. discussed the results and wrote the manuscript.

ACKNOWLEDGMENTS

We thank Professor Andrew T. Hattersley (Institute of Biomedical and Clinical Science, University of Exeter Medical School, Exeter, UK) for important discussions orienting the study. S.A.R.-S. is a career investigator from the Consejo Nacional de Investigaciones Científicas y Técnicas of Argentina (CONICET). This work was supported by the CONICET\Royal Society International Exchanges Cost Share 2018 (24020180100091CO, IEC\R2\181023) to L.V. and S.A.R.-S. The S.A.R.-S. laboratory is funded by grants from Agencia Nacional de Promoción Científica y Tecnológica of Argentina (PICT-2015 3605, PICT-2017 2071) and the Universidad de Buenos Aires (UBACYT 20020170200156BA). The L.V. laboratory is funded by the ERC advanced grant New-Chol, a core support grant from the Wellcome Trust and Medical Research Council of the Wellcome–MRC Cambridge Stem Cell Institute, and the Cambridge University Hospitals NIHR Biomedical Research Centre (BRC-1215-20014). The views expressed are those of the authors and not necessarily those of the NIHR or the Department of Health and Social Care. R.E.K. was funded by the Cambridge Wellcome Trust PhD clinical program, C.Y.C. by an ASTAR studentship, and P.M., D.M., S.V., K.T., and M.C. by the Sanger Wellcome Institute. E.O. is supported by a PhD fellowship from the CONICET.

Received: May 17, 2021
Revised: July 27, 2021
Accepted: July 28, 2021
Published: August 26, 2021

REFERENCES

Barbacci, E., Reber, M., Ott, M.O., Breillat, C., Huetz, F., and Cereghini, S. (1999). Variant hepatocyte nuclear factor 1 is required for visceral endoderm specification. Development *126*, 4795–4805.

Bellanne-Chantelot, C., Clauin, S., Chauveau, D., Collin, P., Daumont, M., Douillard, C., Dubois-Laforgue, D., Dusselier, L., Gautier, J.F., Jadoul, M., et al. (2005). Large genomic rearrangements in the hepatocyte nuclear factor-1beta (TCF2) gene are the most frequent cause of maturity-onset diabetes of the young type 5. Diabetes *54*, 3126–3132.

Beucher, A., Miguel-Escalada, I., Balboa, D., De Vas, M.G., Maestro, M.A., Garcia-Hurtado, J., Bernal, A., Gonzalez-Franco, R., Vargiu, P., Heyn, H., et al. (2021). HASTER is a transcriptional stabilizer of HNF1A. bioRxiv, 2021.2005.2012.443907.

Burgos, J.I., Vallier, L., and Rodríguez-Seguí, S.A. (2021). Monogenic diabetes modeling: in vitro pancreatic differentiation from human pluripotent stem cells gains momentum. Front. Endocrinol. *12*, 692596.

Cebola, I., Rodriguez-Segui, S.A., Cho, C.H., Bessa, J., Rovira, M., Luengo, M., Chhatriwala, M., Berry, A., Ponsa-Cobas, J., Maestro, M.A., et al. (2015). TEAD and YAP regulate the enhancer network of human embryonic pancreatic progenitors. Nat. Cell Biol. *17*, 615–626.

Clissold, R.L., Hamilton, A.J., Hattersley, A.T., Ellard, S., and Bingham, C. (2014). HNF1B-associated renal and extra-renal disease-an expanding clinical spectrum. Nat. Rev. Nephrol. *11*, 102–112.

Coffinier, C., Barra, J., Babinet, C., and Yaniv, M. (1999). Expression of the vHNF1/HNF1beta homeoprotein gene during mouse organogenesis. Mech. Dev. *89*, 211–213.

Conesa, A., Madrigal, P., Tarazona, S., Gomez-Cabrero, D., Cervera, A., McPherson, A., Szcześniak, M.W., Gaffney, D.J., Elo, L.L., Zhang, X., et al. (2016). A survey of best practices for RNA-seq data analysis. Genome Biol. *17*, 13.

Chia, C.Y., Madrigal, P., Denil, S.L.I.J., Martinez, I., Garcia-Bernardo, J., El-Khairi, R., Chhatriwala, M., Shepherd, M.H., Hattersley, A.T., Dunn, N.R., et al. (2019). GATA6 cooperates with EOMES/SMAD2/3 to deploy the gene regulatory network governing human definitive endoderm and pancreas formation. Stem Cell Reports *12*, 57–70.

Cho, C.H., Hannan, N.R., Docherty, F.M., Docherty, H.M., Joao Lima, M., Trotter, M.W., Docherty, K., and Vallier, L. (2012). Inhibition of activin/nodal signalling is necessary for pancreatic differentiation of human pluripotent stem cells. Diabetologia *55*, 3284–3295.

Edghill, E.L., Bingham, C., Ellard, S., and Hattersley, A.T. (2006a). Mutations in hepatocyte nuclear factor-1beta and their related phenotypes. J. Med. Genet. *43*, 84–90.

Edghill, E.L., Bingham, C., Slingerland, A.S., Minton, J.A., Noordam, C., Ellard, S., and Hattersley, A.T. (2006b). Hepatocyte nuclear factor-1 beta mutations cause neonatal diabetes and intrauterine growth retardation: support for a critical role of HNF-1beta in human pancreatic development. Diabet Med. *23*, 1301–1306.

El-Khairi, R., and Vallier, L. (2016). The role of hepatocyte nuclear factor 1β in disease and development. Diabetes Obes. Metab. *18*, 23–32.

Escot, S., Willnow, D., Naumann, H., Di Francescantonio, S., and Spagnoli, F.M. (2018). Robo signalling controls pancreatic progenitor identity by regulating Tead transcription factors. Nat. Commun. *9*, 5082.

Harries, L.W., Brown, J.E., and Gloyn, A.L. (2009). Species-specific differences in the expression of the HNF1A, HNF1B and HNF4A genes. PLoS One *4*, e7855.

Haumaitre, C., Barbacci, E., Jenny, M., Ott, M.O., Gradwohl, G., and Cereghini, S. (2005). Lack of TCF2/vHNF1 in mice leads to pancreas agenesis. Proc. Natl. Acad. Sci. U S A *102*, 1490–1495.

Horikawa, Y., Iwasaki, N., Hara, M., Furuta, H., Hinokio, Y., Cockburn, B.N., Lindner, T., Yamagata, K., Ogata, M., Tomonaga, O., et al. (1997). Mutation in hepatocyte nuclear factor-1 beta gene (TCF2) associated with MODY. Nat. Genet. *17*, 384–385.

Lau, H.H., Ng, N.H.J., Loo, L.S.W., Jasmen, J.B., and Teo, A.K.K. (2018). The molecular functions of hepatocyte nuclear factors â€“ in and beyond the liver. J. Hepatol. *68*, 1033–1048.

Maestro, M.A., Boj, S.F., Luco, R.F., Pierreux, C.E., Cabedo, J., Servitja, J.M., German, M.S., Rousseau, G.G., Lemaigre, F.P., and Ferrer, J. (2003). Hnf6 and Tcf2 (MODY5) are linked in a gene network operating in a precursor cell domain of the embryonic pancreas. Hum. Mol. Genet. *12*, 3307–3314.

Nammo, T., Yamagata, K., Tanaka, T., Kodama, T., Sladek, F.M., Fukui, K., Katsube, F., Sato, Y., Miyagawa, J.-i., and Shimomura, I. (2008). Expression of HNF-4 alpha (MODY1), HNF-1 beta (MODY5), and HNF-1 alpha (MODY3) proteins in the developing mouse pancreas. Gene Expr. Patterns *8*, 96–106.

Rodriguez-Seguel, E., Mah, N., Naumann, H., Pongrac, I.M., Cerda-Esteban, N., Fontaine, J.F., Wang, Y., Chen, W., Andrade-Navarro, M.A., and Spagnoli, F.M. (2013). Mutually exclusive signaling signatures define the hepatic and pancreatic progenitor cell lineage divergence. Genes Dev. *27*, 1932–1946.

Sarkar, S.A., Kobberup, S., Wong, R., Lopez, A.D., Quayum, N., Still, T., Kutchma, A., Jensen, J.N., Gianani, R., Beattie, G.M., et al. (2008). Global gene expression profiling and histochemical analysis of the developing human fetal pancreas. Diabetologia *51*, 285–297.

Sharon, N., Vanderhooft, J., Straubhaar, J., Mueller, J., Chawla, R., Zhou, Q., Engquist, E.N., Trapnell, C., Gifford, D.K., and Melton, D.A. (2019). Wnt signaling separates the progenitor and endocrine compartments during pancreas development. Cell Rep. *27*, 2281–2291.e5.

Sock, E., Rettig, S.D., Enderich, J., Bosl, M.R., Tamm, E.R., and Wegner, M. (2004). Gene targeting reveals a widespread role for the high-mobility-group transcription factor Sox11 in tissue remodeling. Mol. Cell Biol. *24*, 6635–6644.

Taylor, B.L., Liu, F.-F., and Sander, M. (2013). Nkx6.1 is essential for maintaining the functional state of pancreatic beta cells. Cell Rep. *4*, 1262–1275.

Teo, A.K., Lau, H.H., Valdez, I.A., Dirice, E., Tjora, E., Raeder, H., and Kulkarni, R.N. (2016). Early developmental perturbations in a human stem cell model of MODY5/HNF1B pancreatic hypoplasia. Stem Cell Reports *6*, 357–367.

Veres, A., Faust, A.L., Bushnell, H.L., Engquist, E.N., Kenty, J.H., Harb, G., Poh, Y.C., Sintov, E., Gurtler, M., Pagliuca, F.W., et al. (2019). Charting cellular identity during human in vitro beta-cell differentiation. Nature *569*, 368–373.

Wang, D., Wang, J., Bai, L., Pan, H., Feng, H., Clevers, H., and Zeng, Y.A. (2020). Long-term expansion of pancreatic islet organoids from resident Procr(+) progenitors. Cell *180*, 1198–1211.e9.

Wilson, M.E., Yang, K.Y., Kalousova, A., Lau, J., Kosaka, Y., Lynn, F.C., Wang, J., Mrejen, C., Episkopou, V., Clevers, H.C., et al. (2005). The HMG box transcription factor Sox4 contributes to the development of the endocrine pancreas. Diabetes *54*, 3402–3409.

Willmann, S.J., Mueller, N.S., Engert, S., Sterr, M., Burtscher, I., Raducanu, A., Irmler, M., Beckers, J., Sass, S., Theis, F.J., et al. (2016). The global gene expression profile of the secondary transition during pancreatic development. Mech. Dev. *139*, 51–64.

Wolf, F.A., Angerer, P., and Theis, F.J. (2018). SCANPY: large-scale single-cell gene expression data analysis. Genome Biol. *19*, 15.

Yiangou, L., Grandy, R.A., Morell, C.M., Tomaz, R.A., Osnato, A., Kadiwala, J., Muraro, D., Garcia-Bernardo, J., Nakanoh, S., Bernard, W.G., et al. (2019). Method to synchronize cell cycle of human pluripotent stem cells without affecting their fundamental characteristics. Stem Cell Reports *12*, 165–179.

Yu, X.X., Qiu, W.L., Yang, L., Zhang, Y., He, M.Y., Li, L.C., and Xu, C.R. (2019). Defining multistep cell fate decision pathways during pancreatic development at single-cell resolution. EMBO J. *38*, e100164.

Stem Cell Reports, Volume *16*

Supplemental Information

Modeling HNF1B-associated monogenic diabetes using human iPSCs reveals an early stage impairment of the pancreatic developmental program

Ranna El-Khairi, Evelyn Olszanowski, Daniele Muraro, Pedro Madrigal, Katarzyna Tilgner, Mariya Chhatriwala, Sapna Vyas, Crystal Y. Chia, Ludovic Vallier, and Santiago A. Rodríguez-Seguí

Modeling HNF1B-associated monogenic diabetes using human iPSCs reveals an early stage impairment of the pancreatic developmental program

By

Ranna El-Khairi[1,2], Evelyn Olszanowski[3,4], Daniele Muraro[1,2], Pedro Madrigal[1,2], Katarzyna Tilgner[2], Mariya Chhatriwala[1,2], Sapna Vyas[2], Crystal Y. Chia[1,2], Ludovic Vallier[1,2,ϕ], Santiago A. Rodríguez-Seguí[3,5,ϕ]

SUPPLEMENTARY FIGURE LEGENDS

Figure S1. Characterization of the pancreatic differentiation process by studying the expression of relevant lineage markers at different stages

(A) Separated signal and merged panels for representative immunostaining images presented in **Figure 1C**. Scale bar, 100 µm.

(B) Representative immunostaining of HNF1B and other stage-specific markers, including pluripotency markers (OCT4, NANOG, SOX2), endoderm markers (GATA6, SOX17), foregut progenitor markers (HNF4A, HNF1B), posterior foregut and pancreatic progenitor markers (HNF6, PDX1, NKX6.1, SOX9) and endocrine progenitor (PDX1, NKX6.1, NEUROG3) and hormonal cell markers (CHGA, CPEP, GCG), Scale bar, 100 mm.

(C) Expression of pluripotency markers (*POU5F1*, *NANOG*, *SOX2*), endoderm, mesoderm and neuroectoderm markers (*SOX17*, *CXCR4*, *GATA6*, *Brachyury/T*, *PAX6*), foregut progenitor markers (*HNF1B*, *HNF4A*, *FOXA2*), posterior foregut markers (*HNF1B*, *FOXA2*, *HNF4A*, *PDX1*, *SOX9*, *ONECUT1*), pancreatic progenitor markers (*PDX1*, *SOX9*, *NKX6-1*), endocrine progenitor markers (*NEUROG3*, *NEUROD1*, *PDX1*, *NKX6-1*) and hormonal cell markers (*PDX1*, *NKX6-1*, *INS*, *GCG*, *SST*, *PAX6*) during the differentiation of hiPSCs into hormonal cells. mRNA levels were measured by qRT-PCR (n=5 independent experiments at each stage of differentiation using the FSPS13.B wild-type clone) and normalised to the housekeeping gene porphobilinogen deaminase (*PBGD*). Data are presented as mean ± SEM unless otherwise indicated.

Figure S2. Derivation and characterisation of HNF1B mutant hiPSC lines. Early stage differentiation

(A) Schematic showing the human *HNF1B* genomic locus, indicating the protein domains encoded within the *HNF1B* exons.

(B) Genotyping of WT HNF1B+/+, heterozygous HNF1B+/- and homozygous HNF1B-/- mutant hiPSCs. The corresponding sequences of a representative heterozygous mutant line (2nd allele) are shown underneath the WT reference sequence.

(C) mRNA expression of the pluripotency markers *NANOG* and *OCT4* for undifferentiated HNF1B+/+ (D0-1βWT), HNF1B+/- (D0-1βHet) and HNF1B-/- (D0-1βHom) clones for the FSPS13.B and Eipl_1 hiPSC lines (n=3 independent experiments for each clone). The mRNA levels were measured by qRT-PCR and normalized to the house-keeping gene *PBGD*.

(D) Western blot showing expression of HNF1B protein at ~65kb against housekeeping control B-actin (42kb) at the foregut progenitor stage (Day 6) for HNF1B+/+ (D6-1βWT), HNF1B+/- (D6-1βHet) and HNF1B-/- (D6-1βHom) clones for the FSPS13.B and Eipl_1 hiPSC lines.

(E) mRNA expression of *HNF1B* at the foregut progenitor stage (Day 6) for HNF1B+/+ (D6-1βWT), HNF1B+/- (D6-1βHet) and HNF1B-/- (D6-1βHom) clones for the FSPS13.B and Eipl_1 hiPSC lines (n=3 independent experiments). mRNA levels were measured by qRT-PCR and normalised to the house-keeping gene *PBGD*.

(F) Expression of *SOX17* and *CXCR4* in DE cells derived from D6-1βWT, D6-1βHet and D6-1βHom cells. The mRNA levels were measured by qRT-PCR and normalized to the house-keeping gene *PBGD*. Data pooled from n=5 independent experiments for each of the 8 FSPS13.B clones, and n=3 independent experiments for each of the 8 Eipl_1 clones, clone identities as per panel (C).

(G) FACS analysis of cells stained for the DE marker SOX17. There was no significant difference in the number of cells staining for SOX17 in DE cells derived from D6-1βWT, D6-1βHet and D6-1βHom cells. Data pooled from n=5 independent experiments for each of the 8 FSPS13.B clones,

and n=3 independent experiments for each of the 8 Eipl_1 clones, clone identities as per panel (C). Student's t test with two-tailed distribution was used for statistical analysis. All data are presented as mean ± SEM unless otherwise indicated. P-values were not significant.

Figure S3. Differentiation of HNF1B+/+ and HNF1B+/- and HNF1B-/- hiPSCs produces endocrine progenitor (EP) cells (Day 16)

(A) Expression of *NEUROD1*, *NEUROG3* and *GLIS3* in pancreatic progenitor cells derived from HNF1B+/+ (D16-1βWT) and HNF1B+/- (D16-1βHet) and HNF1B-/- (D16-1βHom) hiPSC lines. mRNA levels were measured by qRT-PCR and normalized to the house-keeping gene *PBGD*. Data pooled from n=5 independent experiments for each of the 8 FSPS13.B clones, with identities as per **Figure S2C**. Student's t test with two-tailed distribution was used for statistical analysis. All data are presented as mean ± SEM unless otherwise indicated. $^{*}p < 0.05$; $^{**}p < 0.01$; $^{***}p < 0.001$ and $^{****}p < 0.0001$.

(B) Percentage of cells expressing PDX1, NKX6.1 and NEUROD1 and representative FACS dot plots of cells stained for PDX1 and NEUROD1. The percentage of each cell population is indicated in the corresponding quadrant for all FACS plots. Replicates and statistics as indicated in panel (A).

Figure S4. Differential expression of key pancreatic differentiation markers between HNF1B+/+ (1βWT) and HNF1B+/- (1βHet) and HNF1B-/- (1βHom) cells as quantified from RNA-seq data.

(A) Gene expression for key foregut and pancreatic markers as detected by RNA-seq (n = 3 at each stage of differentiation and for each genotype). Counts were normalized using the fragments per kilobase of transcript per million mapped reads (fpkm) function of the DESeq2 package. Data are presented as mean ± SEM unless otherwise indicated.

(B) Venn diagrams showing the overlap of downregulated and upregulated genes in D13-1βHom and D13-1βHet, when compared to D13-1βWT cells. Selected genes cording for transcription factors are listed next to the Venn diagrams.

Figure S5. Gene expression variation in stem, progenitor and endocrine cells derived in vitro from HNF1B+/+ and HNF1B+/- and HNF1B-/- hiPSCs

(A) Principal component analysis of expressed genes (counts >1) in HNF1B+/+ (1βWT), HNF1B+/- (1βHet) and HNF1B-/- (1βHom) cells. n= 3 independent experiments per sample.

(B) Heatmap of sample-to-sample distances using log-transformed values. Rectangles correspond to measurements from individual biological replicates.

Figure S6. scRNA-seq analysis reveals cell populations derived in vitro from 1βWT and 1βHet iPSCs

(A) Violin plots showing the expression for selected markers in human in vitro derived pancreatic cells clustered as in **Figure 6A**. Cluster PROCR+ express EMT markers.

(B) Feature and violin plots showing the expression for selected EMT and PROCR+ markers (Wang et al. 2020) in human in vitro derived pancreatic cells clustered as in **Figure 6A**.

(C) Total number of cells per cluster and differentiation stage or genotype.

Figure S7. HNF1B haploinsufficiency impairs the early stage pancreatic developmental program by altering expression of key non-canonical Wnt and Hippo signalling pathway components

(A) Dot plot showing expression of the top markers significantly up- and downregulated in 1βHet samples for the progenitor cell clusters as presented in **Figure 6A**. Color intensity indicates mean expression (normalized) in a cluster, dot size indicates the proportion of cells in a cluster expressing the gene.

(B) Percent of genes up or downregulated in 1βHet early MPC and late MPC which are associated with at least 1 MPC enhancer and/or TEAD1/HNF1B binding sites. MPC enhancers taken from Cebola et al, Nat Cell Biol 17, 615-626.

(C) UCSC genome browser snapshots of the *SFRP5* and *SOX11* genomic loci. ChIP-seq was used to locate binding sites of HNF1B, ONECUT1, FOXA2 and TEAD1 in MPCs (data from Cebola et al. 2015). ChIP-seq for H3K4me1 and H3K27ac histone modifications denotes the epigenomic printing of active enhancers. MPC enhancers enriched in HNF1B signal in this locus are highlighted in yellow.

(D) Dot plot showing expression of *ROBO1*, *ROBO2*, selected Hippo pathway components and its known target *CTGF*. Color intensity indicates mean expression (normalized) in a cluster, dot size indicates the proportion of cells in a cluster expressing the gene.

SUPPLEMENTARY TABLES

Table S1. Summary of genotypes for the targeted clones for FSPS13.B (top) and Eipl_1 (bottom) hiPSC lines. The number of clones with no integration of the puromycin resistance cassette (HNF1B WT clones) or integration of the puromycin resistance cassette in one or two alleles (HNF1B homozygous knockout) of the HNF1B gene is shown. For clones where there is integration of the puromycin resistance cassette in one allele, the 2nd allele was either WT (HNF1B heterozygous knockout) or contained an in-frame or frameshift mutation (HNF1B homozygous knockout).

Table S2. Alignment details for raw bulk RNA-seq and ChIP-seq data used in this study.

Table S3. Differentially expressed genes resulting from pairwise comparisons of samples derived from cells with different HNF1B genotypes.

Table S4. Gene ontology results. Significant down- and up-regulated gene ontology biological process (GO - BP) pathways in 1βWT vs βHet and 1βWT vs 1βHom pairwise comparisons at days 6, 8, 13, 16 and 27 of the differentiation protocol.

Table S5. Day13 10x single-cell RNA-seq initial clustering. Top 50 cell cluster markers.

Table S6. Day13 10x single-cell RNA-seq differentially expressed per genotype in each cluster ($p<0.05$).

Table S7. 1βHet differentially regulated genes in early MPC and late MPC that are associated with at least 1 MPC enhancer and/or TEAD1/HNF1B binding sites.

Khairi_FigS1

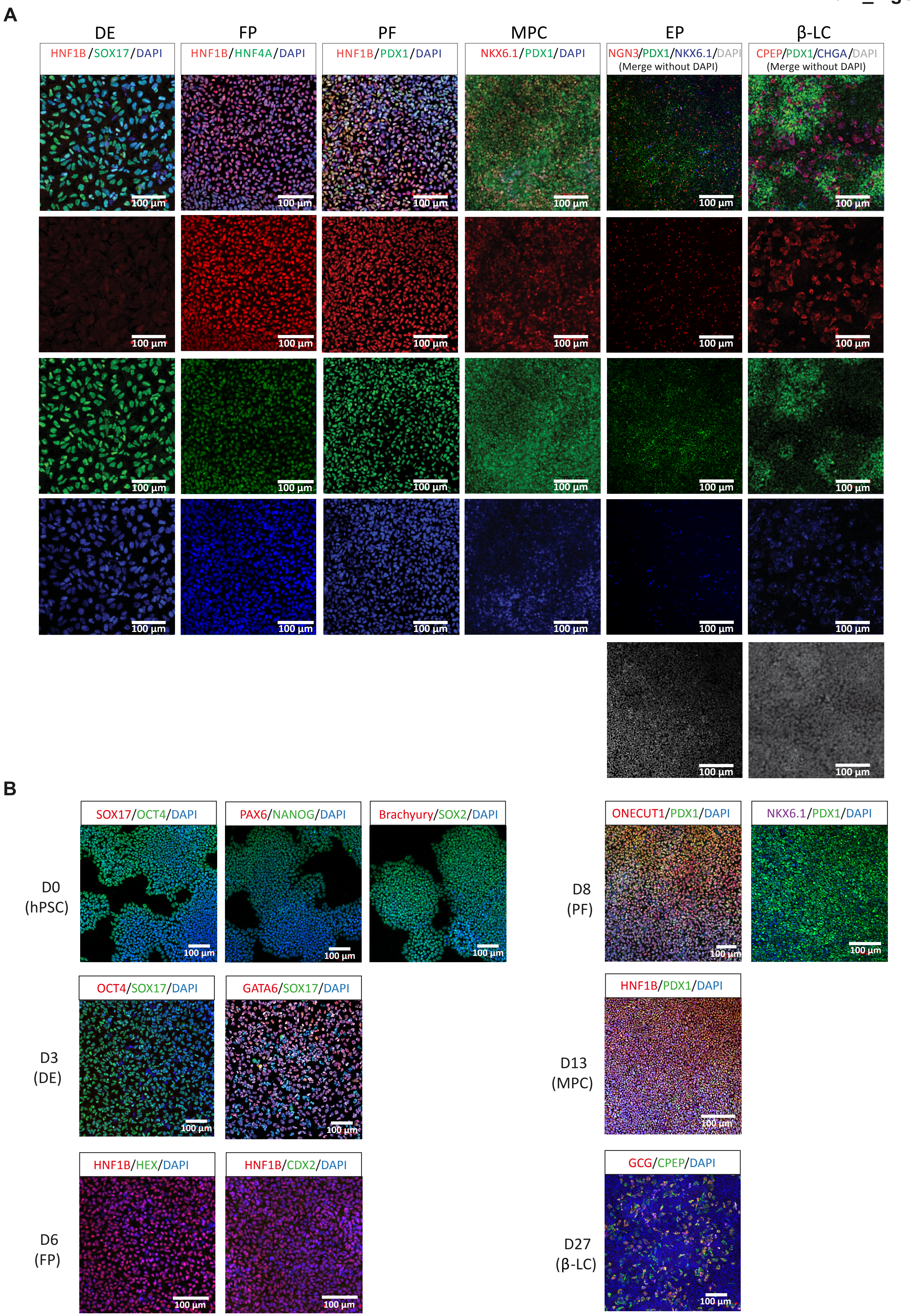

Khairi_FigS1 (cont)

C

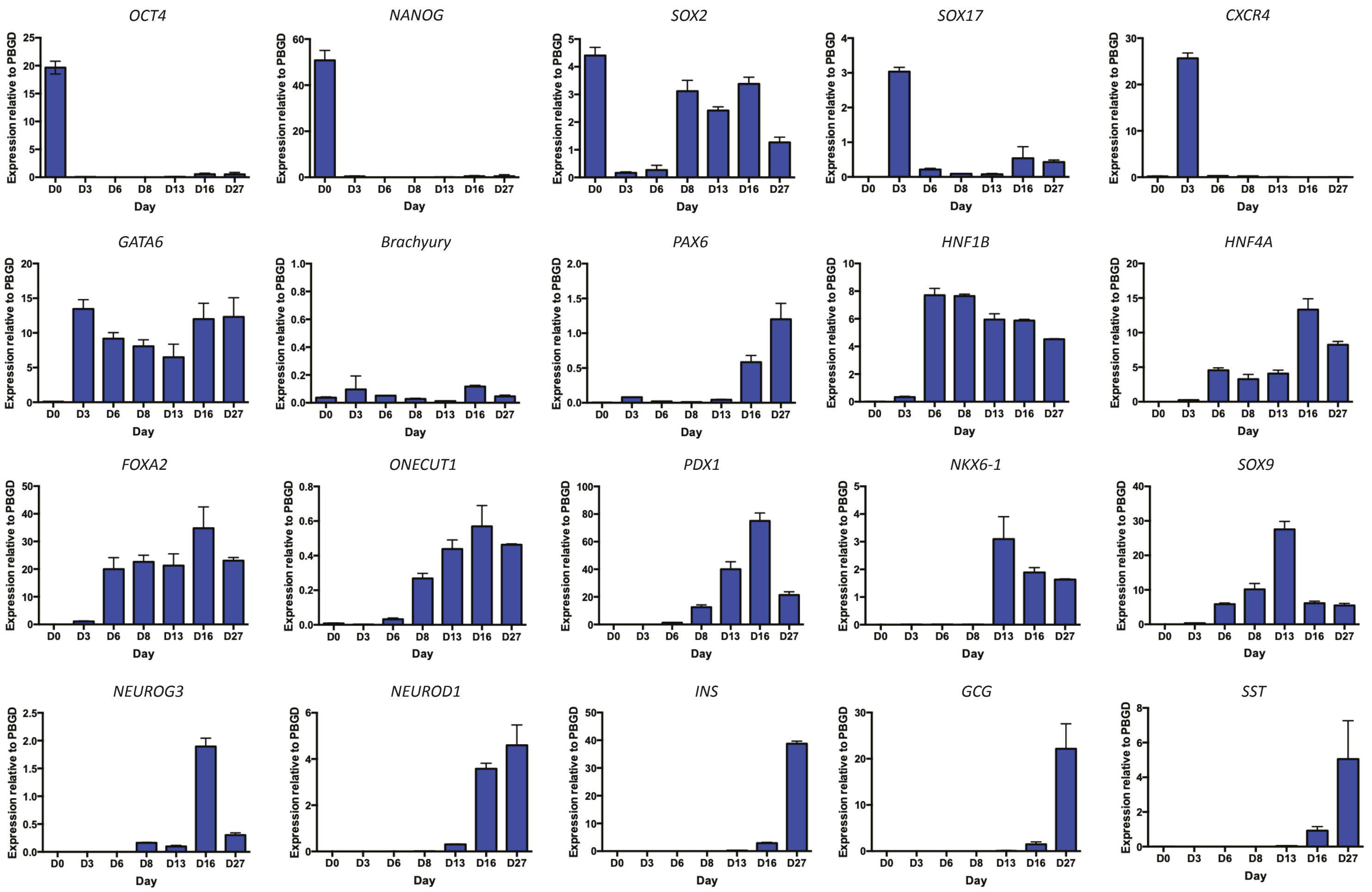

Khairi_FigS2

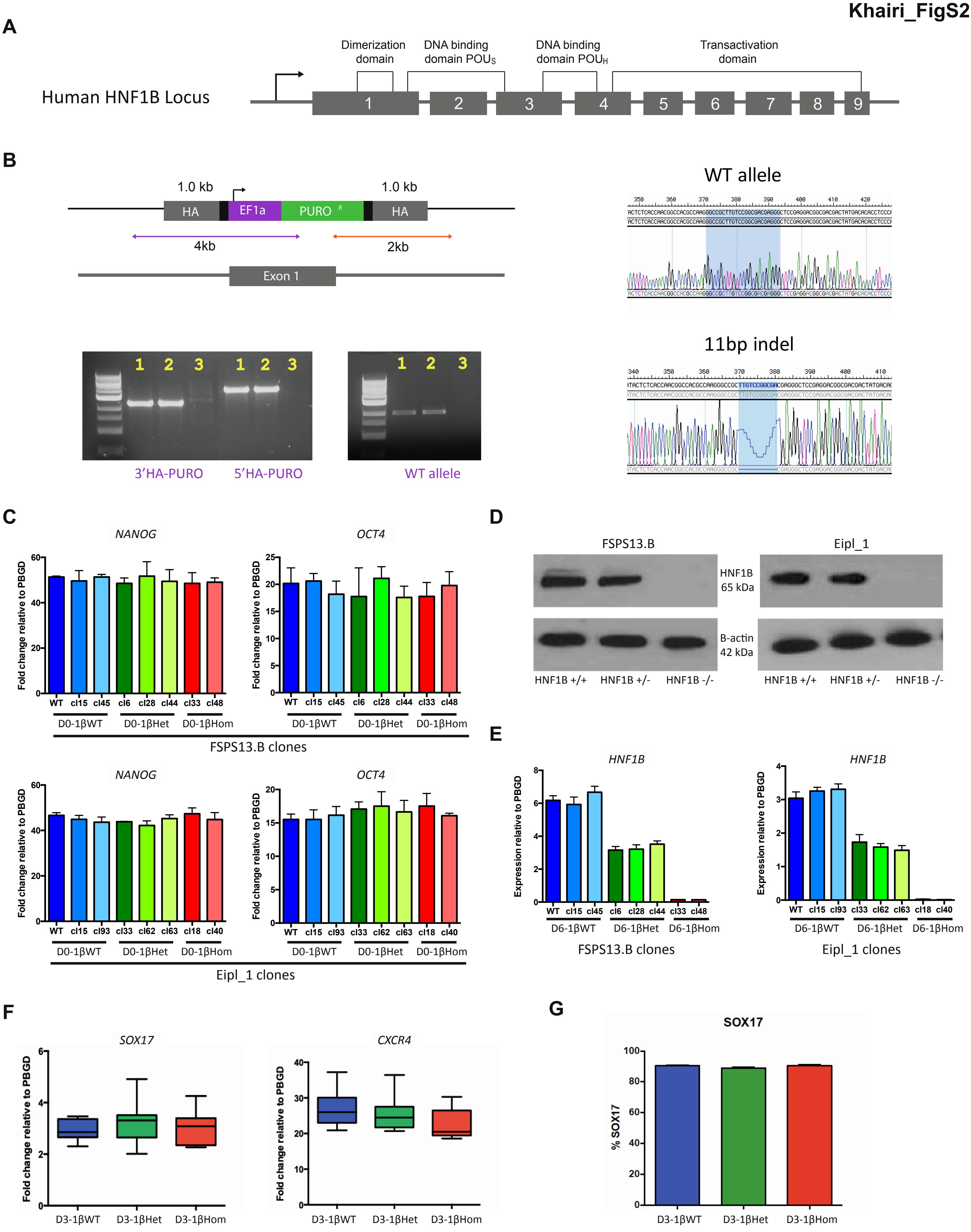

Khairi_FigS3

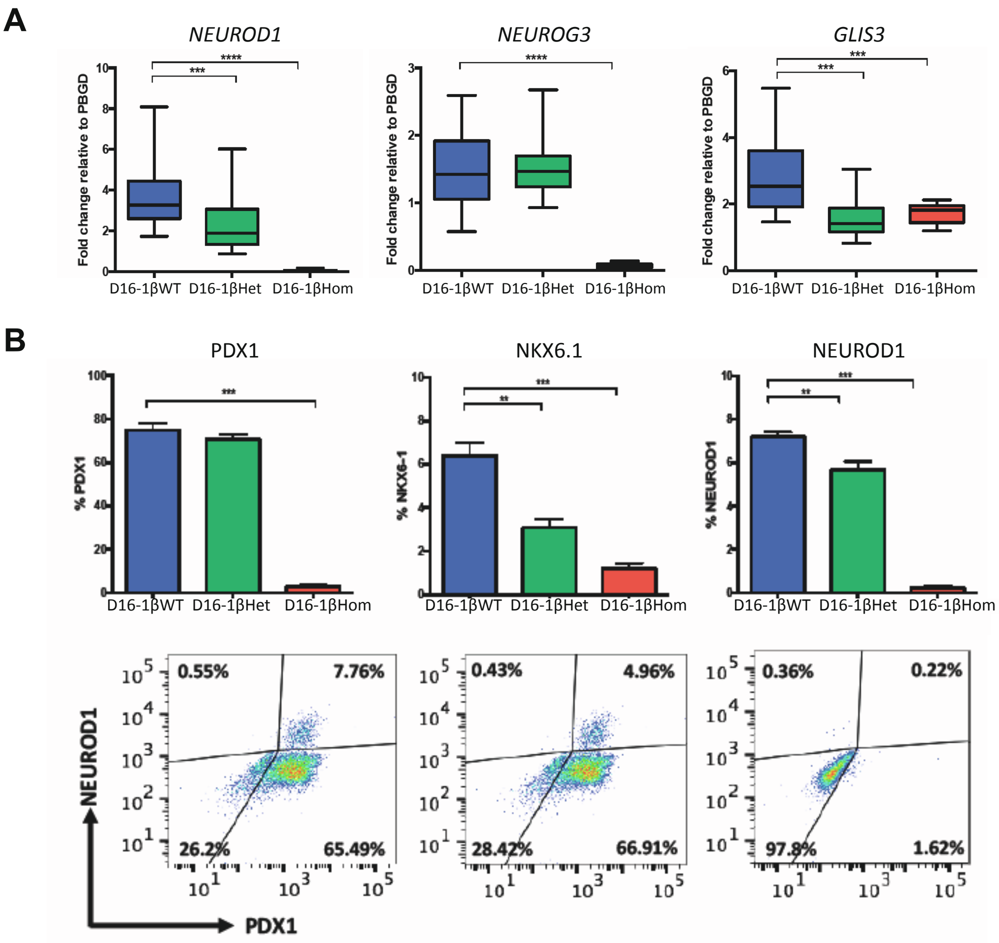

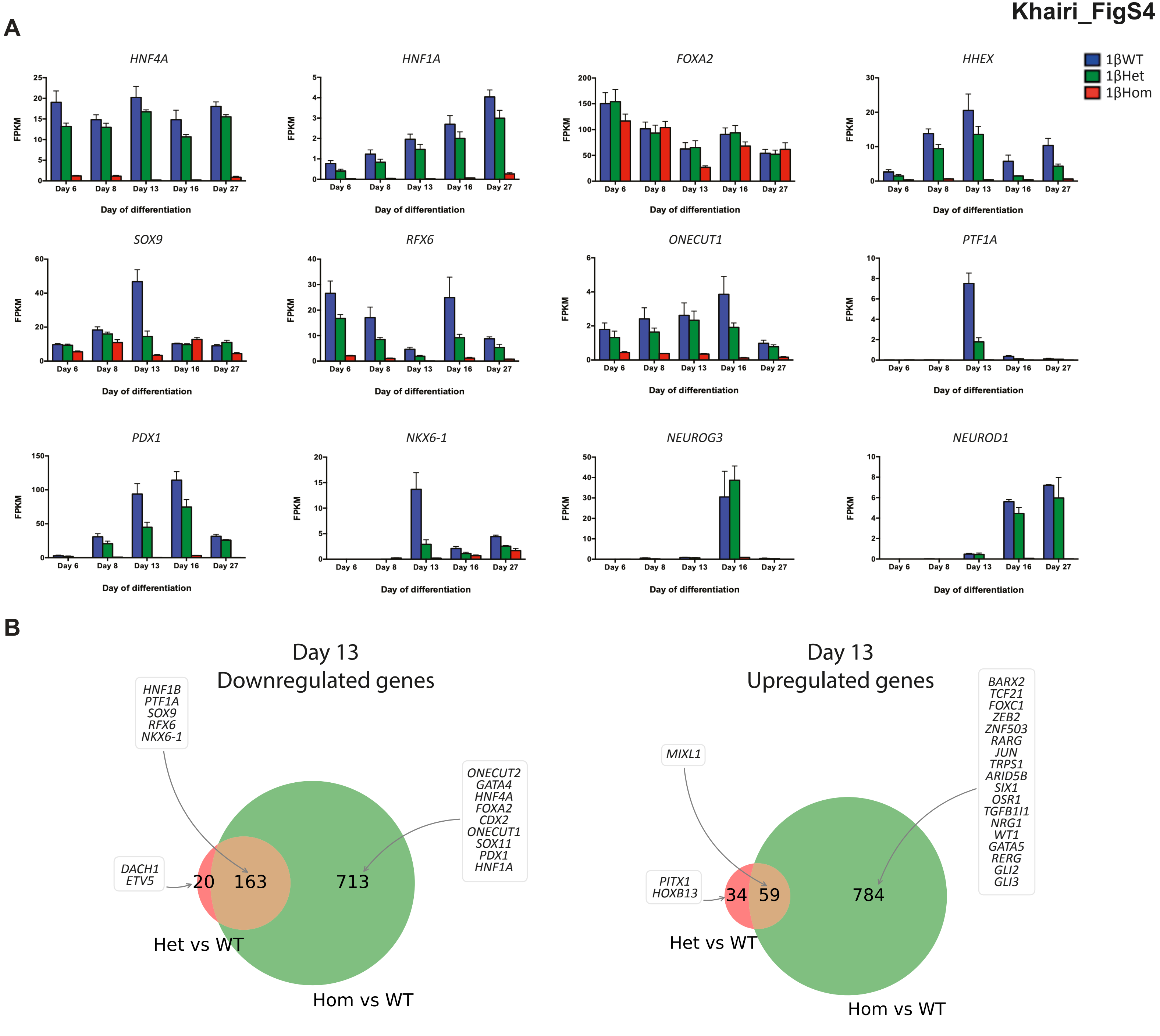
A
HNF4A
HNF1A
FOXA2
HHEX
1βWT
1βHet
1βHom
SOX9
RFX6
ONECUT1
PTF1A
PDX1
NKX6-1
NEUROG3
NEUROD1
FPKM
Day 6
Day 8
Day 13
Day 16
Day 27
Day of differentiation
B
Day 13
Downregulated genes
HNF1B
PTF1A
SOX9
RFX6
NKX6-1
ONECUT2
GATA4
HNF4A
FOXA2
CDX2
ONECUT1
SOX11
PDX1
HNF1A
DACH1
ETV5
20
163
713
Het vs WT
Hom vs WT
Day 13
Upregulated genes
MIXL1
BARX2
TCF21
FOXC1
ZEB2
ZNF503
RARG
JUN
TRPS1
ARID5B
SIX1
OSR1
TGFB1I1
NRG1
WT1
GATA5
RERG
GLI2
GLI3
PITX1
HOXB13
34
59
784
Het vs WT
Hom vs WT

A

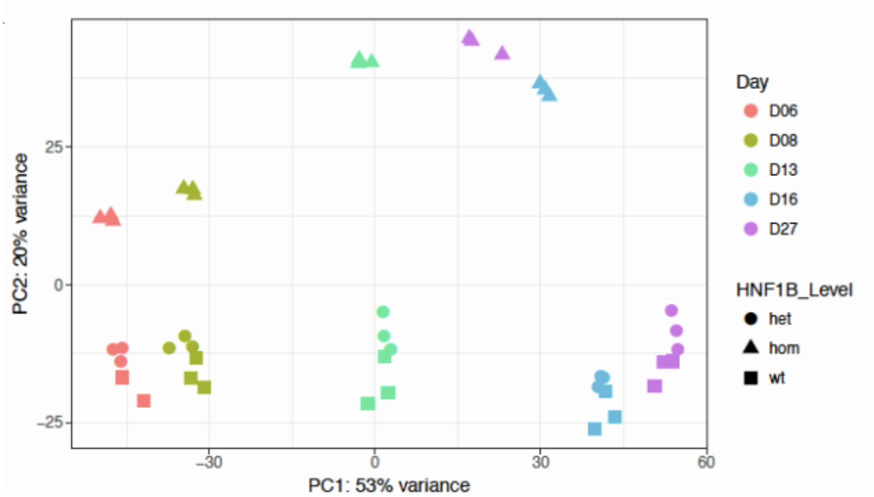

PC2: 20% variance
PC1: 53% variance
25
0
–25
–30
0
30
60
Day
D06
D08
D13
D16
D27
HNF1B_Level
het
hom
wt

B

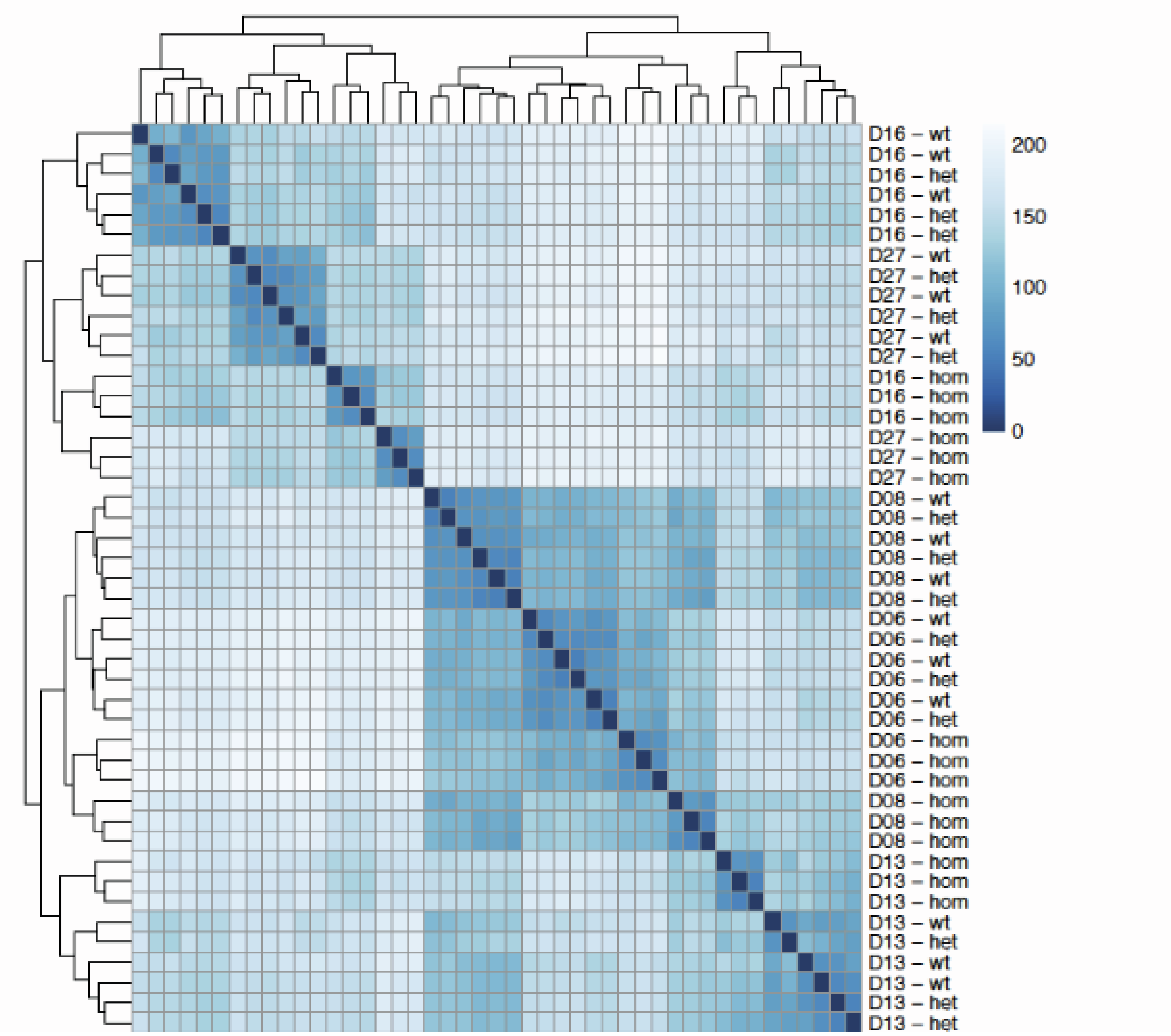

D16 – wt
D16 – wt
D16 – het
D16 – wt
D16 – het
D16 – het
D27 – wt
D27 – het
D27 – wt
D27 – het
D27 – wt
D27 – het
D16 – hom
D16 – hom
D16 – hom
D27 – hom
D27 – hom
D27 – hom
D08 – wt
D08 – het
D08 – wt
D08 – het
D08 – wt
D08 – het
D06 – wt
D06 – het
D06 – wt
D06 – het
D06 – wt
D06 – het
D06 – hom
D06 – hom
D06 – hom
D08 – hom
D08 – hom
D08 – hom
D13 – hom
D13 – hom
D13 – hom
D13 – wt
D13 – het
D13 – wt
D13 – wt
D13 – het
D13 – het
200
150
100
50
0

A

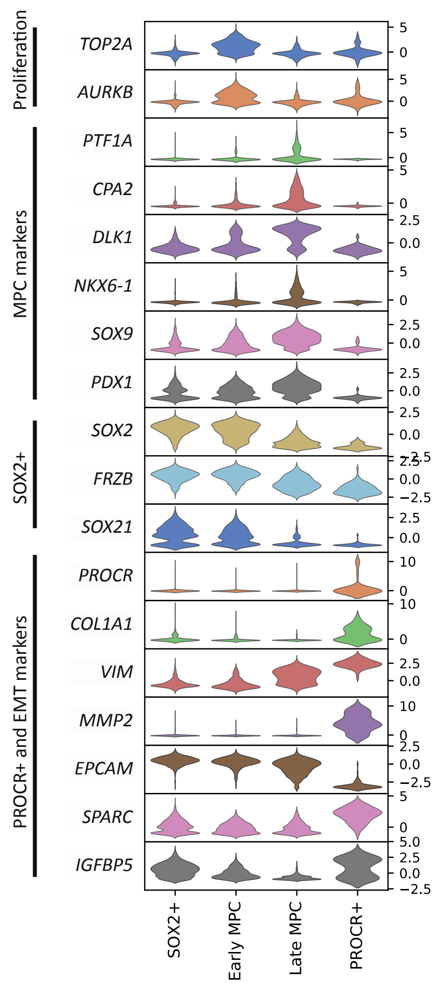

B

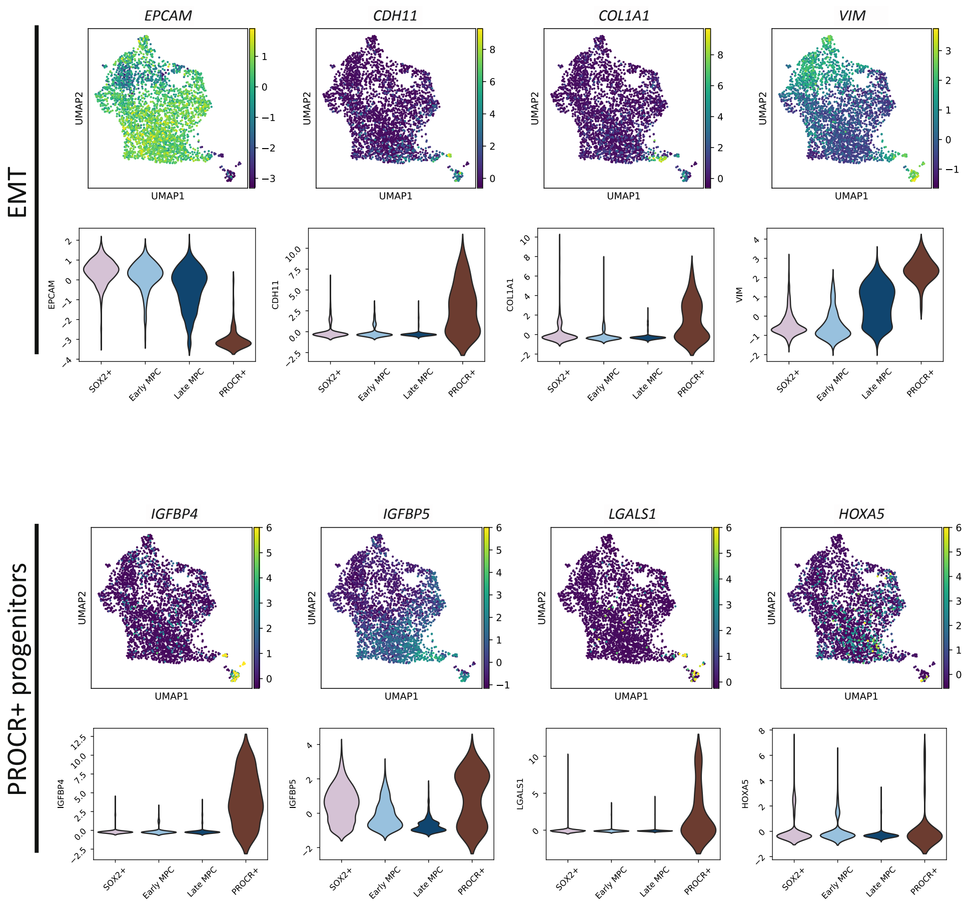

C

Genotype	Cluster #				Total # cells/genotype
	SOX2+	Early MPC	Late MPC	PROCR+	
1βHet	765	302	677	25	1769
1βWT	653	317	425	52	1447

Khairi_FigS7

A

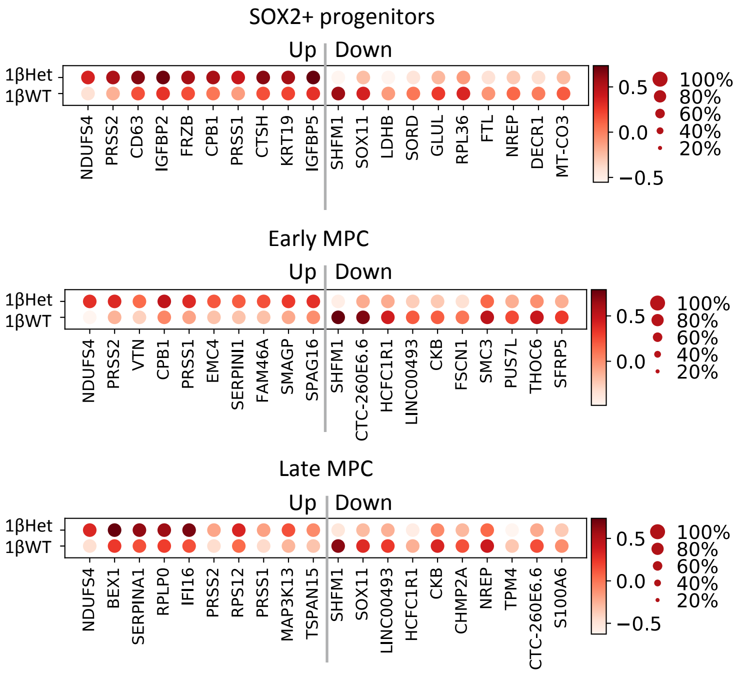

C

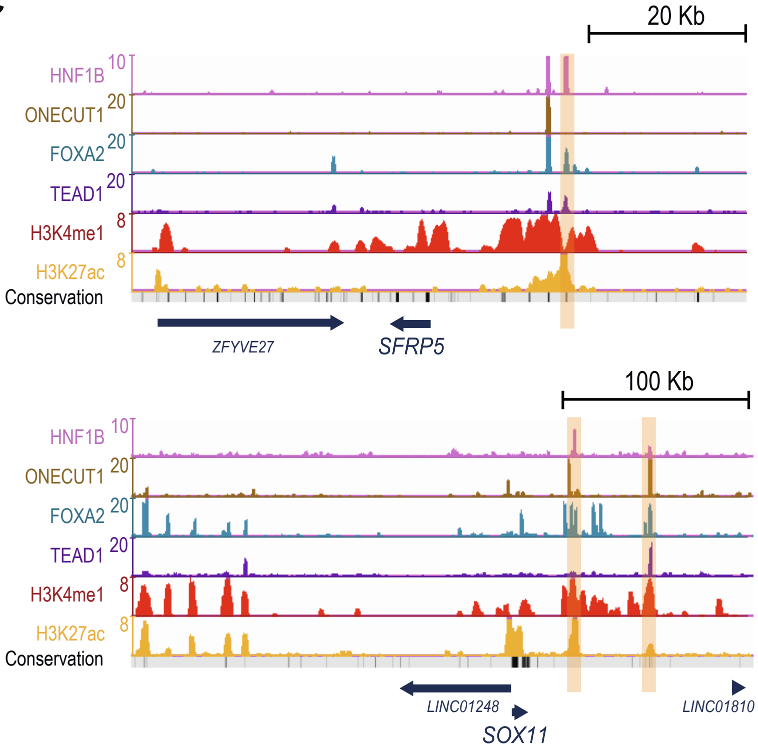

B

	Percent of genes up or downregulated in 1βHet early MPC and late MPC which are associated with at least one:			
	MPC enhancer	MPC enhancer + TEAD1	MPC enhancer + HNF1B	MPC enhancer + HNF1B + TEAD1
% of Markers **up** in **1βHet Early MPC**	50.0	47.1	29.4	29.4
% of Markers **down** in **1βHet Early MPC**	45.1	36.6	22.0	20.7
% of Markers **up** in **1βHet Late MPC**	39.6	33.6	16.8	14.8
% of Markers **down** in **1βHet Late MPC**	40.9	36.2	21.7	20.9

D

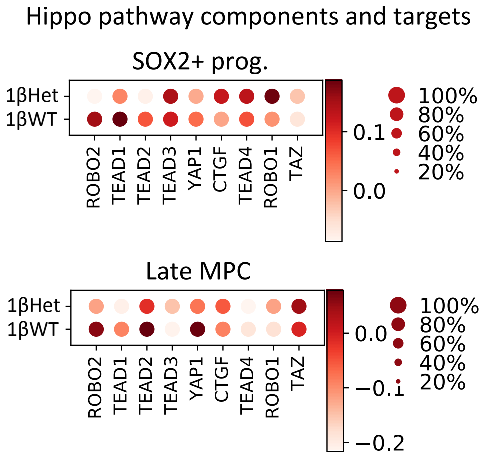

Table S1. Summary of genotypes for the targeted clones for FSPS13.B (top) and Eipl_1 (bottom) hiPSC lines. The number of clones with no integration of the puromycin resistance cassette (HNF1B WT clones) or integration of the puromycin resistance cassette in one or two alleles (HNF1B homozygous knockout) of the HNF1B gene is shown. For clones where there is integration of the puromycin resistance cassette in one allele, the 2nd allele was either WT (HNF1B heterozygous knockout) or contained an in-frame or frameshift mutation (HNF1B homozygous knockout).

**FSPS13.B**	**1 st allele**	**2 nd allele**	**Number of targeted clones**
**WT**	WT	WT	4
**Heterozygous**	Puro R cassette	WT	3
**Homozygous**	Puro R cassette	Puro R cassette	1
	Puro R cassette	NHEJ (frameshift mutation)	14 (4)

**Eipl_1**	**1 st allele**	**2 nd allele**	**Number of targeted clones**
**WT**	WT	WT	8
**Heterozygous**	Puro R cassette	WT	5
**Homozygous**	Puro R cassette	Puro R cassette	1
	Puro R cassette	NHEJ (frameshift mutation)	29 (10)

NHEJ: non-homologous end joining.

Table S2. Alignment details for raw bulk RNA-seq and ChIP-seq data used in this study.

Experiment	Sample name	Day	HNF1B genotype / Tissue	Library size	Mapped reads	Source
bulk RNA-seq in in vitro derived pancreatic progenitor and endocrine cells	13B Cl.45 D6_1	6	WT	118384519	88543640	This study
	13B Cl.6 D6_1	6	Het	116131701	86113194	This study
	13B Cl.48 D6_1	6	Hom	111558545	85740614	This study
	13B Cl.45 D6_2	6	WT	111655388	84139726	This study
	13B Cl.6 D6_2	6	Het	129777107	98644498	This study
	13B Cl.48 D6_2	6	Hom	111826972	88110998	This study
	13B Cl.45 D6_3	6	WT	139606811	103703665	This study
	13B Cl.6 D6_3	6	Het	106679668	80614687	This study
	13B Cl.48 D6_3	6	Hom	128714446	98328972	This study
	13B Cl.45 D8_1	8	WT	123888095	93134642	This study
	13B Cl.6 D8_1	8	Het	147013538	112050531	This study
	13B Cl.48 D8_1	8	Hom	123679567	93162048	This study
	13B Cl.45 D8_2	8	WT	127493326	98438445	This study
	13B Cl.6 D8_2	8	Het	127798804	95737326	This study
	13B Cl.48 D8_2	8	Hom	122936103	92031674	This study
	13B Cl.45 D8_3	8	WT	117085741	86900383	This study
	13B Cl.6 D8_3	8	Het	119824087	90038548	This study
	13B Cl.48 D8_3	8	Hom	119466266	90660554	This study
	13B Cl.45 D13_1	13	WT	142169329	102528677	This study
	13B Cl.6 D13_1	13	Het	135094009	93279477	This study
	13B Cl.48 D13_1	13	Hom	112511990	85968763	This study
	13B Cl.45 D13_2	13	WT	118693241	83580395	This study
	13B Cl.6 D13_2	13	Het	132360151	94662742	This study
	13B Cl.48 D13_2	13	Hom	140083454	103631461	This study
	13B Cl.45 D13_3	13	WT	127000896	96077132	This study
	13B Cl.6 D13_3	13	Het	117594841	84714110	This study
	13B Cl.48 D13_3	13	Hom	122503320	92967061	This study
	13B Cl.45 D16_1	16	WT	117505611	81310336	This study
	13B Cl.6 D16_1	16	Het	130164511	91700486	This study
	13B Cl.48 D16_1	16	Hom	141466373	100263082	This study
	13B Cl.45 D16_2	16	WT	147170171	96305978	This study
	13B Cl.6 D16_2	16	Het	147452466	87898580	This study
	13B Cl.48 D16_2	16	Hom	133070182	90936195	This study
	13B Cl.45 D16_3	16	WT	113804780	84558461	This study
	13B Cl.6 D16_3	16	Het	134215663	93391591	This study
	13B Cl.48 D16_3	16	Hom	120543917	81094469	This study
	13B Cl.45 D27_1	27	WT	125249986	90666868	This study
	13B Cl.6 D27_1	27	Het	116555579	87162835	This study
	13B Cl.48 D27_1	27	Hom	116216729	85354846	This study
	13B Cl.45 D27_2	27	WT	143787152	94199999	This study
	13B Cl.6 D27_2	27	Het	118236331	82906196	This study
	13B Cl.48 D27_2	27	Hom	127138325	80287366	This study
	13B Cl.45 D27_3	27	WT	133940466	92711641	This study
	13B Cl.6 D27_3	27	Het	130352438	93655003	This study
	13B Cl.48 D27_3	27	Hom	137048178	95732442	This study
ChIP-seq	FOXA2	NA	*In vitro* MPCs	25760337	21579594	E-MTAB-1990 *
	ONECUT1	NA	*In vitro* MPCs	25606334	20745554	E-MTAB-1990 *
	HNF1B	NA	*In vitro* MPCs	25645350	19915667	E-MTAB-1990 *
	TEAD1	NA	*In vitro* MPCs	25813944	20389873	E-MTAB-3061 *
	H3K27ac	NA	*In vitro* MPCs	22219409	17739290	E-MTAB-3061 *
	H3K4me1	NA	*In vitro* MPCs	51072767	44298878	E-MTAB-1990 *
	INPUT	NA	*In vitro* MPCs	49562149	37147369	E-MTAB-1990 *

* data from ArrayExpress Archive

Table S5. Day13 10x single-cell RNA-seq initial clustering. Top 50 cell cluster markers

Marker #	Cluster			
	SOX2+	Early MPC	Late MPC	PROCR+
1	RP11-834C11.4	NDFIP1	HMGB2	TFPI
2	SEMA3C	MEST	H2AFZ	VIM
3	PTPN13	FTL	SMC4	COL3A1
4	SOX2	DLK1	CENPU	TMSB10
5	DSP	SERPINB6	CKS1B	MAP1B
6	IGFBP5	CLU	CDK1	TPM4
7	IGFBP2	AMBP	KIAA0101	RHOC
8	TMSB4X	DUSP5	ZWINT	SFRP1
9	TAGLN2	LAPTM4B	TUBA1B	HEY1
10	ANXA3	PHGDH	NUSAP1	PFN1
11	PERP	SOX9	UBE2C	PHLDA1
12	KLF5	VIM	MAD2L1	PTMS
13	NR2F1	PABPC1	HMGN2	TMSB4X
14	PAM	HMGCS2	HMGB1	COL1A2
15	PPDPF	TTYH1	ASPM	PTN
16	MYL12B	ALDH1A1	BRCA2	GNG11
17	PLEKHA5	RAMP1	CENPF	ETS1
18	CD63	LDHB	DEK	ACTG1
19	ANXA2	ZFP36L2	PRC1	NES
20	NPW	GATM	TUBB	SPARC
21	IGDCC3	KIRREL2	CENPW	MAGED2
22	CLDN4	COX7C	CBX5	MMP2
23	PDLIM1	BTG1	TUBB4B	IFITM3
24	UCP2	SCD	NUCKS1	FAM212A
25	GSTP1	RAB3B	SGOL1	BGN
26	TPM1	PKDCC	TOP2A	CFL1
27	FOXP1	BEX1	MKI67	BASP1
28	EPCAM	CPA2	SMC2	IGFBP4
29	KRT19	SERINC5	TPX2	IFITM2
30	PGM2L1	RPLP1	RAD51AP1	TLN1
31	CLDN6	NQO2	KIF11	ARPC2
32	CTSH	APOE	TK1	SEPT11
33	RUNX1	SLC4A4	TYMS	SPRY1
34	SPINT1	TM4SF4	C21orf58	COL4A1
35	UNC13C	ECE1	RRM2	TUBA1A
36	TNNC1	PDX1	PTMA	ATP5E
37	WFDC2	LIN28A	BIRC5	THY1
38	DSTN	CAMK2N1	KIF15	HSPB1
39	PRSS8	TCF7L2	ORC6	39326
40	SPINT2	ID2	ATAD5	MEF2C
41	ELF1	PLK2	MIS18BP1	ITGA1
42	FRZB	SMC3	CENPK	FILIP1
43	CD9	JAG1	KIF20B	ACTB
44	STARD10	LAMA1	PBK	PLAT
45	HOTAIRM1	ATP5A1	CLSPN	MLLT11
46	EPSTI1	PRTG	TACC3	SERPINH1
47	CELF2	HEY1	DIAPH3	NFIA
48	C2orf54	FN1	TMPO	ANXA6
49	SOX21	FLRT3	NUF2	TPM2
50	EZR	RPLP0	DLGAP5	SNCA

EXPERIMENTAL PROCEDURES

hiPSC generation and characterization

Two hiPSC lines, FSPS13.B and Eipl_1 were used for genome editing and pancreatic differentiation experiments. The hiPSCs were derived from human skin fibroblasts and peripheral blood (http://www.hipsci.org/lines/#/lines/HPSI0813i-fpdm_2, http://www.hipsci.org/lines/#/lines/hpsi0114i-eipl_1). Ethics approval was obtained from the National Research Ethics Service (NRES) Committee East of England, Cambridge East (Ethics reference no. 09/h0304/77).

hiPSC culture

Undifferentiated hiPSCs were routinely cultured under feeder-free conditions on vitronectin-coated (STEMCELL Technologies #07180) tissue culture plates (Corning) with Essential 8 Medium (Life Technologies #A1517001). The medium was changed every day, and cells were passaged every 4-5 days using 0.5 mM EDTA (Life Technologies, #15575-020) to dissociate cells. In all hiPSC cultures, 10 μM Rho-associated protein kinase (ROCK) inhibitor, Y-27632 (Selleck Chemicals, #S1049), was only added into the culture media when thawing hiPSCs. Human iPSCs were maintained at 37°C with 5% CO_2 and regularly tested negative for mycoplasma contamination and for chromosomal aberrations.

Cell preparation for pancreatic differentiation

Human iPSCs were passaged and seeded onto 12-well plates using E8 supplemented with Y27632 (Rho-associated, coiled-coil containing protein kinase, ROCK Inhibitor; 10μM). Cells were plated as single cells and the plating density was optimised for each hiPSC line (approximately 80,000 to 100,000 cells per well of a 12-well plate). After 24 hours, the media was replaced with fresh E8 media without ROCK Inhibitor.

Pancreatic differentiation protocol

To induce definitive-endoderm differentiation (days 1-3), cells were cultured in CDM-PVA supplemented with Activin (100ng/ml), FGF2 (80ng/ml), BMP4 (10ng/ml), Ly294002 (10μM) and CHIR99021 (3μM) on day 1, CDM-PVA supplemented with Activin (100ng/ml), FGF2 (80ng/ml), BMP4 (10ng/ml) and Ly294002 (10μM) on day 2 then RPMI/B27 media containing Activin (100ng/ml), FGF2 (80ng/ml) on day 3. For primitive gut tube differentiation (days 4-6), cells were cultured in Adv-BSA media supplemented with SB-431542 (10μM), FGF10 (50ng/ml), RA (3μM), Noggin (150μg/ml) and L-Ascorbic acid (250μM) for 3 days. For posterior foregut differentiation (days 7-8), cells were cultured in Adv-BSA with FGF10 (50ng/ml), RA (3μM), Noggin (150μg/ml), KAAD-cyclopamine (0.238μM), PdBU (50nM) and L-Ascorbic acid (250μM). Pancreatic progenitor specification (days 9- 13) was induced by culturing cells in RA (1μM), Noggin (150μg/ml), KAAD-cyclopamine (200ng/ml), EGF (100ng/ml), Nicotinamide (10mM), and L-Ascorbic acid (250μM) for 5 days. Cells were then grown in Adv-BSA containing glucose (final concentration 25mM), B27 (1%), RA (100nM), DAPT (1μM), Alk5i (10μM) and the small molecule BNZ (0.1mM) for 3 days to induce maturation of pancreatic progenitors to endocrine progenitor cells (fifth stage; days 14-16). For maturation of endocrine cells and further differentiation into C-peptide-producing beta cells, cells were cultured for 3 additional days in Adv-BSA containing B27 (1%), RA (100nM) and Alk5i (10μM) followed by 11 days in Adv-BSA containing B27 (1%), RA (100nM) (sixth stage; days 17-27).

List of abbreviations used in **Figure 1A**: A, activin A; F, fibroblast growth factor 2; B, bone morphogenetic protein; Ly, LY294002; Chir, Chir99021; B27, B-27 Supplement® (ThermoFisher Scientific, Waltham, MA, USA); RA; retinoic acid; NOG, noggin; SB; SB-431542; F10, fibroblast growth factor 10; VitC, Vitamin C; Cyclo, cyclopamine; PdBU, phorbol 12,13-dibutyrate; EGF, epidermal growth factor; NA, nicotinamide; Alk5i, TGFβ type I receptor kinase (Alk5) inhibitor; BNZ, 6-Benzoyladenosine-3',5'-cyclic monophosphate; DAPT, N-(N [3,5-diflurophenylacetyl]-L-alanyl)-S-phenylglycine t-butyl ester. CDM, chemically defined medium; PVA, Polyvinyl Alcohol; RPMI, Roswell Park Memorial Institute medium; Adv-DMEM/F-12; Advanced Dulbecco's Modified Eagle Medium/Ham's F-12.

Generation of clonal hiPSC mutant lines

Assembly of Cas9, gRNA and donor vectors. Cas9 nuclease target regions in exon 1 of the HNF1B gene and suitable guide RNA (gRNA) sequences were identified using the CRISPR design tool provided by the Zhang laboratory (Ran et al., 2013). The human codon-optimized Cas9 expression plasmid was obtained from Addgene (hCas9 Plasmid #41815,). To construct the gRNA vector, gRNA sequences were cloned into a U6 BsaI gRNA backbone vector, containing a hU6 promoter and a Kanamycin resistance cassette (obtained from Professor Bill Skarnes' group at the Wellcome Trust Sanger Institute, Hinxton, Cambridge). The success of the gRNA assembly was verified by Sanger sequencing.

A donor vector aimed at terminating transcription of HNF1B prematurely by inserting a 'donor template' through homologous recombination was also constructed. The donor vector contains 5' and 3' homology arms each 1kb in length recognising the flanking regions of the gRNA target site, an Ef1a promoter, a puromycin antibiotic resistant cassette and a polyA tail. The final construct was sequenced to confirm that the donor vector was cloned successfully.

Electroporation and screening of drug-resistant clones. Cas9 nuclease, gRNA and final donor vectors were transfected into cells using the Amaxa Nucleofector® Technology and Human Stem Cell Nucleofector® Kit 1 (Lonza, #VAPH-5012). Cells were harvested into a single cell suspension using Accutase (Stem Cell Technologies #07920). 1 x 10^6 cells were used for each nucleofection. Nucleofection was performed according to the manufacturer's instructions using Nucleofector® Program B-016. Following nucleofection, cells were plated in E8 media supplemented with ROCK inhibitor. 48 hours after nucleofection, selection was commenced using puromycin (1 µg/ml; Sigma-Aldrich, #P8833) for 5 days. Single colonies were picked and screened to detect wild-type, heterozygous and homozygous HNF1B knockout clones.

Multiplex fluorescence in situ hybridization (M-FISH) karyotyping

For each cell line, 10-20 randomly selected metaphases were karyotyped based on multiplex fluorescence *in situ* hybridization (M-FISH) with human 24-colour painting probe and DAPI-banding pattern analyses.

Western blotting

Cells were harvested and lysed using the cell lysis buffer, CelLytic M reagent (Sigma-Aldrich, C2978) with PhosStop phosphatase inhibitor cocktail (Roche, # 4906837001) and cOmplete protease inhibitor cocktail (Roche, #11697498001). Cell lysates were centrifuged at 14,000 rpm at 4°C for 5 minutes. The supernatants were collected and protein concentrations were determined by Bradford assay (Protein Assay Dye Reagent Concentrate, Bio-Rad) according to the manufacturer's protocol. The normalized cell lysates were heat denatured then subjected to SDS-PAGE electrophoresis on NuPAGE Novex 4-12% Bis-Tris Protein Gels using the XCell SureLock Mini-Cell (Invitrogen) system. The protein samples were next transferred onto a PVDF membrane (Bio-Rad, #162-0177) using the Mini Trans-Blot Cell (Bio-Rad). Membranes were blocked in 4% powdered skimmed milk diluted in 0.05% Tween-20 in D-PBS for 1 hour at room temperature. The membrane was incubated with primary antibodies overnight at 4°C, followed by incubation with horseradish peroxidase (HRP)-conjugated secondary antibodies for 1 hr at room temperature. Protein bands were detected via chemiluminescence using the Pierce ECL detection kit (Thermo Fisher Scientific). Antibodies used for western blotting are listed below.

Immunofluorescence (IF) staining

Cells in 12 well plates were fixed in 500 μl of 4% paraformaldehyde (PFA; VWR, #43368.9M) solution for 20 min at 4°C. They were then washed thrice in D-PBS followed by blocking in 10% donkey serum (AbD Serotec, #C06SB) in 0.1% Triton X-100 in D-PBS (PBST) for 20 min at room temperature. Cells were then incubated overnight at 4°C with primary antibodies diluted in 1% donkey serum in PBST. Cells were then washed thrice with PBST and incubated with fluorescence-dye conjugated secondary antibodies diluted in 1% donkey serum in PBST for 1 hr

at room temperature. Antibodies used for immunostaining are listed below. Images were taken using a Zeiss LSM 700 confocal microscope (Carl Zeiss, Jena, Germany).

Fluorescence activated cell sorting (FACS) analysis

Cells in 12 well plates were washed twice in D-PBS and incubated in Accutase for 5 min at 37°C. The cells were dissociated by gentle pipetting and then re-suspended and washed twice with D-PBS. The cell suspension was filtered through a 40µm filter and then fixed by incubating in 4% PFA solution diluted in D-PBS for 20 min at 4°C, then washed twice in D-PBS. Cell surface marker staining; CXCR4 or Live/dead staining (LIVE⁄DEAD Fixable Violet Dead Cell Stain Kit; Molecular Probes, #L34955, 1:1,000); was performed by incubating cells in FACS buffer or 5% FBS in D-PBS for 30 minutes or 1 hour on ice. For intracellular staining, cells were permeabilised in 1% saponin (Sigma-Aldrich, #47036-50G-F) in D-PBS for 30 min at room temperature. Cells were then incubated with primary antibody diluted in staining solution (1% saponin and 5% FBS in D-PBS) for 2 hr at room temperature. Cells were washed three times with staining solution and incubated with secondary antibodies diluted in staining solution for 30 min at room temperature. Cells were washed thrice in staining solution and re-suspended in 2% FBS diluted in D-PBS prior to analysis. Analyses were performed using a BD LRSFortessa cell analyser (BD Biosciences). Data analyses were performed using FlowJo. All gates shown on scatterplots were set according to the undifferentiated population control. Antibodies used for FACS analyses are listed below.

Antibodies used in this work

The antibodies used in this work for western blotting, IF and/or FACS included: Goat anti-NANOG (R&D, AF1997, RRID:AB_355097), Goat anti-OCT3/4 (Santa Cruz, sc8628, RRID:AB_653551), Mouse anti-OCT3/4 (Santa Cruz, sc5279, RRID:AB_628051), Goat anti-SOX2 (R&D, AF2018, RRID:AB_355110), Rabbit anti-SOX2 (Millipore, AB5603, RRID:AB_2286686), Goat anti-SOX17 (R&D, AF1924, RRID:AB_355060), Goat anti-Brachyury (R&D, AF2085,

RRID:AB_2200235), Rabbit anti-PAX6 (Cambridge BioScience , PRB-278P-100, RRID:AB_291612), Rabbit anti-EOMES (Abcam, ab23345, RRID:AB_778267), Goat anti-FOXA2 (R&D, AF2400, RRID:AB_2294104), Mouse anti-CXCR4 (R&D, MAB173, RRID:AB_2089398), Mouse anti-GATA4 G-4 (Santa Cruz, sc25310, RRID:AB_627667), Rabbit anti-GATA6 D61E4 (CST, #5851, RRID:AB_10705521), Goat anti-HNF1B C-20 (Santa Cruz, sc7411, RRID:AB_2116769), Rabbit anti-HNF1B (Santa Cruz, sc22840, RRID:AB_2279595), Rabbit anti-HNF4A H-171 (Santa Cruz, sc8987, RRID:AB_2116913), Mouse anti-HEX (Abcam, ab117864, RRID:AB_10900199), Mouse anti-CDX2 (CDX-88) (Abcam, ab86949, RRID:AB_10671889), Goat anti-PDX1 (R&D, AF2419, RRID:AB_355257), Rabbit anti-PDX1 (CST, #5679, RRID:AB_10706174), Rabbit anti-HNF6 H-100 (Santa Cruz, sc13050, RRID:AB_2251852), Rabbit anti-SOX9 (Millipore, AB5535, RRID:AB_2239761), Mouse anti-NKX6-1 (DSHB, F55A12, RRID:AB_532379), Sheep anti-NGN3 (R&D, AF3444, RRID:AB_2149527), Goat anti-GCC G-17 (Santacruz, sc7780, RRID:AB_641025), Rabbit anti-SST (Daka, A0566, RRID:AB_2688022), Mouse anti-C-PEP (Acris Antibodies, BM270S, RRID:AB_978884), Rat anti-INS (DSHB, GN-ID4, RRID:AB_2255626), Goat anti-CHGA (Santa Cruz, sc1488, RRID:AB_2276319), Mouse anti-Alpha-tubulin (Sigma, T6199, RRID:AB_477583), Donkey anti-goat 488 (Invitrogen, A-11055, RRID:AB_2534102), Donkey anti-mouse 488 (Invitrogen, A-21202, RRID:AB_141607), Donkey anti-rat 488 (Invitrogen, A-21208, RRID:AB_141709), Donkey anti-sheep 488 (Invitrogen, A-11015, RRID:AB_141362), Donkey anti-rabbit 488 (Invitrogen, A-21206, RRID:AB_2535792), Donkey anti-goat 647 (Invitrogen, A-21447, RRID:AB_141844), Donkey anti-mouse 647 (Invitrogen, A-31571, RRID:AB_162542), Donkey anti-sheep 647 (Invitrogen, A21448, RRID:AB_1500712), Donkey anti-rabbit 647 (Invitrogen, A-31573, RRID:AB_2536183), Goat anti-mouse HRP (Sigma, A2554, RRID:AB_258008), Goat anti-rabbit HRP (Sigma, A0545, RRID:AB_257896).

RNA isolation and qRT-PCR

Total RNA was isolated using the RNeasy Mini Kit (Qiagen, #74106) and eluted in 30 µl of RNase free water. 500 ng of isolated total RNA was used for cDNA synthesis with SuperScript II Reverse Transcriptase (Invitrogen, #18064014). Quantitative Real-Time PCR was performed using the SensiMix SYBR Low-Rox Kit (Bioline, #QT625-20) on the Mx3005P Real-Time PCR system (Agilent) according to the manufacturer's instructions. Samples were run in technical triplicates and normalized to PBGD. Gene-specific primers are listed below:

**Gene**	**Primer**	**Sequence (5' to 3')**
PBGD	F	GGAGCCATGTCTGGTAACGG
	R	CCACGCGAATCACTCTCATCT
POU5F1 / OCT4	F	AGTGAGAGGCAACCTGGAGA
	R	ACACTCGGACCACATCCTTC
NANOG	F	CATGAGTGTGGATCCAGCTTG
	R	CCTGAATAAGCAGATCCATGG
SOX2	F	TGGACAGTTACGCGCACAT
	R	CGAGTAGGACATGCTGTAGGT
SOX17	F	CGCACGGAATTTGAACAGTA
	R	GGATCAGGGACCTGTCACAC
CXCR4	F	CACCGCATCTGGAGAACCA
	R	GCCCATTTCCTCGGTGTAGTT
GATA6	F	TTCGTTTCCTGGTTTGAATTCC
	R	TGCAATGCTTGTGGACTCTAC
Brachyury	F	TGCTTCCCTGAGACCCAGTT
	R	GATCACTTCTTTCCTTTGCATCAAG
PAX6	F	CTTTGCTTGGGAAATCCGAG
	R	AGCCAGGTTGCGAAGAACTC
FOXA2	F	GGGAGCGGTGAAGATGGA
	R	TCATGTTGCTCACGGAGGAGTA
HNF1B	F	TCACAGATACCAGCAGCATCAGT
	R	GGGCATCACCAGGCTTGTA
HNF4A	F	CATGGCCAAGATTGACAACCT
	R	TTCCCATATGTTCCTGCATCAG
HEX	F	GCCCTTTTACATCGAGGACA
	R	AGGGCGAACATTGAGAGCTA
ONECUT1	F	GTGTTGCCTCTATCCTTCCCAT
	R	CGCTCCGCTTAGCAGCAT
PTF1A		Hs_PTF1A_1_SG QuantiTect Primer Assay, Quiagen QT0021802
SOX9		Hs_SOX9_1_SG QuantiTect Primer Assay, Qiagen QT00001498
MNX1	F	CACCGCGGGCATGATC
	R	ACTTCCCCAGGAGGTTCGA
PDX1	F	AAGTCTACCAAAGCTCACGCG

	R	GTAGGCGCCGCCTGC
NKX6-1	F	GGCCTGTACCCCTCATCAAG
	R	TCCGGAAAAAGTGGGTCTCG
NEUROG3	F	GCTCATCGCTCTCTATTCTTTTGC
	R	GGTTGAGGCGTCATCCTTTCT
NEUROD1	F	AGACGCTTTGCAAGGGCTTA
	R	TCCGAGGATTGAGTTGCAGG
GLIS3	F	GTCCATGGATTTTATGGGCAGC
	R	CAAACGAAGGCACCACACTG
CHGA	F	GCAGAGGACCAGGAGCTAGA
	R	CAGGGGCTGAGAACAAGAGA
INS	F	CAGGAGGCGCATCCACA
	R	AAGAGGCCATCAAGCAGATCA
GCG	F	AAGCATTTACTTTGTGGCTGGATT
	R	TGATCTGGATTTCTCCTCTGTGTCT
SST	F	CCCCAGACTCCGTCAGTTTC
	R	TCCGTCTGGTTGGGTTCAG

RNA-seq experiments

For the bulk RNA-seq experiments, one HNF1B$^{+/+}$, one HNF1B$^{+/-}$ and one HNF1B$^{-/-}$ (targeted wild-type) clone from the FSPS13.B hiPSC line were differentiated along the pancreatic lineage. RNA was extracted using the GenElute Mammalian Total RNA Miniprep Kit (Sigma-Aldrich) according to manufacturer's protocol. RNA-seq library construction and sequencing was carried out by the DNA pipelines core facility at the Wellcome Sanger Institute. Standard Illumina unstranded poly-A enriched libraries were prepared and sequenced using Illumina HiSeq 2500 v4 (Illumina, San Diego, CA, USA), with 75bp paired-end reads per sample and a library fragment size of 100-1000 bp. Three independent experiments (biological triplicates generated from FSPS13.B clones) were sequenced for each clone at each stage of differentiation.

RNA-seq data analysis and functional annotation

Bioinformatics analyses were carried out following standard procedures (Conesa et al., 2016). Reads were aligned to the reference human genome assembly GRCh38 with TopHat v2.0.13 (Kim et al., 2013) with a transcript index built using Ensembl version 76 gene annotation. Reads

with Mapping Quality Value <10 were filtered out with Samtools. featureCounts was used on paired-end reads to count fragments in annotated gene features, with parameters '-p -C -T 8 -t exon -g gene_id' (Liao et al., 2014), and genes with no counts were filtered out. Differentially expressed genes were identified using DESeq2 R/Bioconductor package (Love et al., 2014). Genes with fold change ≥2 and adjusted p-value (using Benjamin-Hochberg correction) <0.05 were identified as differentially expressed (**Table S3**). Counts were normalized using the fragments per kilobase of transcript per million mapped reads (FPKM). Principal component plot of the samples was performed in DESeq2. Sample information together with the total number of aligned fragments and mapped reads with quality score ≥10 are shown in **Table S2**. For further analysis (**Figure 4**), differentially expressed genes were then selected if the three replicates had an expression value >0.5 FPKM in at least one of the genotypes. Functional annotation was performed with DAVID (Huang da et al., 2009), using the Gene Ontology (GO) tool, focusing in the biological processes (BP). Significantly enriched terms were defined with a Benjamini value >0.05 (**Table S4**).

Apoptosis assay

Apoptotic cells were detected using the Annexin V Apoptosis Detection Kit FITC (Thermo Fisher Scientific, Waltham, MA, USA). Cells were detached and harvested using Accutase and washed once in PBS, then once in 1x Binding Buffer. Cells were filtered and then resuspended in 1x Binding Buffer at 2.5x106 cells/ml. Cells were subsequently aliquoted in four conditions – Annexin V only, Annexin V and Propidium Iodide (PI), PI only, and no staining. 5µL of fluorochrome-conjugated Annexin V was added to 100µl of the cell suspension and incubated for 15 minutes at room temperature. After two washes with 1x Binding Buffer, cells were incubated for 10 minutes with 5µL PI (10µg/ml) to visualize dead cells. Cells were analysed by flow cytometry within four hours of staining. Flow cytometric data were analysed with FlowJo software.

Cell proliferation assay

Cell proliferation was measured using the Click-iT® EdU Flow Cytometry Assay Kit (Invitrogen). 5-ethynyl-2'-deoxyuridine (EdU) was added to the culture medium at 10µM for 2 hours. Cells were then detached and harvested using Accutase as previously discussed. 1x106 cells were aliquoted and washed once with 3mL of 1% BSA in PBS. Cells were then incubated in 100µL of Click-iT fixative for 15 minutes at room temperature, protected from light. Cells were washed with 3mL of 1% BSA in PBS and incubated in 100µl of 1x Click-iT saponin based permeabilization and wash reagent for 15 minutes. 500µl of Click-iT reaction cocktail (CuSO4 10µl, Pacific Blue azide 2.5µl, 1x Reaction Buffer Additive 50µl, and PBS 438µl) was added to each sample and the reaction mixture was incubated for 30minutes at room temperature, protected from light. Cells were washed once with 3mL of 1x Click-iT saponin based permeabilization and wash reagent and then resuspended in 1mL of 1x Click-iT saponin based for staining for DNA content using the FxCycle™ Far Red stain (Invitrogen). 1µl of FxCycle™ Far Red stain (final concentration 200nM) and 20mg/mL of RNase A was added to each flow cytometry sample and incubated for 30 minutes at room temperature. FxCycle™ Far Red stains RNA as well as dsDNA, so addition of RNase A (Thermo Fisher Scientific) is required for DNA content analysis. Samples were analysed on a flow cytometer without washing, using 405/450nm (Pacific Blue) and 640/670nm (Far Red) excitation and emission spectra.

Glucose-stimulated insulin secretion

Human iPSC-derived pancreatic endocrine cells at day 27 were preincubated in DMEM supplemented with 2.5µM glucose (low glucose media) for 60 minutes at 37°C. To measure basal C-peptide release, cells were incubated in low glucose media for 60 minutes at 37°C. To estimate glucose-induced C-peptide secretion, the media was replaced by DMEM supplemented with 22.5 mM glucose (high glucose media) and alternatively with DMEM supplemented with 2.5 mM glucose for 60 minutes at 37°C. The low glucose – high glucose stimulation was repeated for a second cycle. Finally, cells were incubated in low-glucose DMEM with 30mM potassium

chloride (KCl) for 30 min. The supernatant at each stage was collected and stored at -80°C for determination of C-peptide release. C-peptide ELISA was measured using the Mercodia C-peptide ELISA kit (Mercodia, Uppsala, Sweden), following the manufacturer's recommendations. Absorbance was read at 450nm on an Infinite 200 Pro plate reader (Tecan, Männedorf, Switzerland).

Preparation of differentiated cells for single-cell sequencing

Single cell libraries from D13.1βHet and D13.1βWT samples were generated using the Chromium Single Cell 3′ library and gel bead kit v2 (PN #120237) from 10x Genomics. Briefly, to reach a target cell number of 2,000 cells per sample, 3,500 cells per sample were loaded onto a channel of the 10x chip to produce Gel Bead-in-Emulsions (GEMs). This underwent reverse transcription to barcode RNA before cleanup and cDNA amplification followed by enzymatic fragmentation and 5′adaptor and sample index attachment. Libraries were sequenced on the HiSeq4000 (Illumina) with 125 bp paired-end sequencing.

Analysis of single cell RNA-seq data

Filtering, alignment to the GRCh38 human genome version 28 (Ensembl 92) and unique molecular identifier (UMI)-collapsing were performed using the Cell Ranger (v2.01) pipeline with default mapping arguments (10X Genomics). All further analyses were run with python 3 using the Scanpy API package (Wolf et al., 2018). To further remove low quality cells, we filtered cells with a high fraction of counts from mitochondrial genes (7% or more) indicating stressed or dying cells, and cells expressing less than 3000 genes. In addition, genes with expression in less than 10 cells were excluded. To improve the quality of the analysis, we also excluded outlier cells with >7000 genes detected, or with less that 1.5% fraction of counts from mitochondrial genes. Cell by gene count matrices of D13-1βWT and D13-1βHet samples were then concatenated to a single matrix and each cell was then normalized by total counts over all genes,

so that every cell has the same total count after normalization. Values were next log transformed. This output matrix was input to all further analyses.

Low dimensional embedding, visualization and clustering. A single-cell neighbourhood graph was computed on the 30 first principal components using 40 nearest neighbours. Clusters were identified using the Louvain algorithm (with resolution = 0.4) as implemented in louvain-igraph (https://github.com/vtraag/louvain-igraph) and adopted by Scanpy. Visualisation in 2D was performed using the dimensionality reduction algorithm UMAP (Uniform Manifold Approximation and Projection). Cell types were annotated based on the expression of known marker genes.

Marker gene identification and subtype characterization. Characteristic gene signatures were identified by testing for differential expression of a subgroup against all other cells or between two subgroups as outlined in the text using the tl.rank_genes_groups function of Scanpy, setting the Wilcoxon rank-sum test as statistical method.

Pseudotime analysis. To infer a pseudotemporal ordering of the cells, diffusion pseudotime (dpt) (Haghverdi et al., 2016) was used as implemented in Scanpy (tl.dpt) setting a root cell within the starting population (SOX2+ cluster).

Software specifications. All analyses from UMI count matrices were run with python 3 with the Scanpy API v.1.4 and anndata v.0.6.19. Versions of packages required by Scanpy that might influence numerical results are as follows: numpy v.1.16.2, scipy v.1.2.1, pandas v.0.24.1, scikit-learn v.0.20.2, statsmodels v.0.9.0, python-igraph v.0.8.2, louvain v.0.6.1.

Analysis of ChIP-seq data

Publicly available raw datasets were obtained from the Sequence Read Archive (SRA) database as listed in **Table S2**. Raw sequence reads were aligned to the human (UCSC hg19) genome using Bowtie v1.1.2 (Langmead et al., 2009), and further processed as previously described (Cebola et al., 2015; Pasquali et al., 2014). In brief, only sequences uniquely aligned with ≤1 mismatch were retained. Post-alignment processing of sequence reads included *in silico*

extension and signal normalization based on the number of million mapped reads. Reads were extended to a final length equal to MACS fragment size estimation (Zhang et al., 2008), and only unique reads were retained. For signal normalization, the number of reads mapping to each base in the genome was counted using the genomeCoverageBed command from BedTools (Quinlan and Hall, 2010). Processed files were visualized in the UCSC genome browser (Kent et al., 2002). Transcription factor enrichment sites were detected with MACS v1.4.2 (Zhang et al., 2008) using default parameters and a *P* value of 10^{-5}. Transcription factor binding sites were associated to the nearby genes using GREAT v4.0.4 with default settings

Quantification and statistical analysis

For both FSPS13.B and Eipl_1, we used 3 wild-type clones (HNF1B+/+; one non-targeted wild-type and two targeted wild-type clones), 3 heterozygous clones (HNF1B+/-) and 2 homozygous clones (HNF1B-/-; one with puromycin cassette in both alleles and one with puromycin cassette in 1st allele and indel in 2nd allele). The clone identities are shown in **Figures S2C, S2E**. The data in the main and supplementary figures are pooled from experiments using FSPS13.B and Eipl_1 clones for qPCR, flow cytometry and Elisa. Quantification data are presented as mean ± SEM. Data from clonal lines of the same genotype were combined for calculating the significance of the differences between different genotypes. To directly compare two groups, Student's t test with two-tailed distribution was used to test for statistical significance. P values less than 0.05 was considered statistically significant. All statistical analyses were performed using GraphPad Prism 6.0 (GraphPad Software Inc., San Diego, CA, USA) or the R statistical environment.

REFERENCES

Cebola, I., Rodriguez-Segui, S.A., Cho, C.H., Bessa, J., Rovira, M., Luengo, M., Chhatriwala, M., Berry, A., Ponsa-Cobas, J., Maestro, M.A., *et al.* (2015). TEAD and YAP regulate the enhancer network of human embryonic pancreatic progenitors. Nat Cell Biol *17*, 615-626.

Conesa, A., Madrigal, P., Tarazona, S., Gomez-Cabrero, D., Cervera, A., McPherson, A., Szcześniak, M.W., Gaffney, D.J., Elo, L.L., Zhang, X., *et al.* (2016). A survey of best practices for RNA-seq data analysis. Genome Biology *17*, 13.

Haghverdi, L., BÃ¼ttner, M., Wolf, F.A., Buettner, F., and Theis, F.J. (2016). Diffusion pseudotime robustly reconstructs lineage branching. Nature Methods *13*, 845-848.

Huang da, W., Sherman, B.T., and Lempicki, R.A. (2009). Systematic and integrative analysis of large gene lists using DAVID bioinformatics resources. Nat Protoc *4*, 44-57.

Kent, W.J., Sugnet, C.W., Furey, T.S., Roskin, K.M., Pringle, T.H., Zahler, A.M., and Haussler, D. (2002). The human genome browser at UCSC. Genome Res *12*, 996-1006.

Kim, D., Pertea, G., Trapnell, C., Pimentel, H., Kelley, R., and Salzberg, S.L. (2013). TopHat2: accurate alignment of transcriptomes in the presence of insertions, deletions and gene fusions. Genome Biology *14*, R36.

Langmead, B., Trapnell, C., Pop, M., and Salzberg, S.L. (2009). Ultrafast and memory-efficient alignment of short DNA sequences to the human genome. Genome Biol *10*, R25.

Liao, Y., Smyth, G.K., and Shi, W. (2014). featureCounts: an efficient general purpose program for assigning sequence reads to genomic features. Bioinformatics *30*, 923-930.

Love, M.I., Huber, W., and Anders, S. (2014). Moderated estimation of fold change and dispersion for RNA-seq data with DESeq2. Genome Biology *15*, 550.

Pasquali, L., Gaulton, K.J., Rodriguez-Segui, S.A., Mularoni, L., Miguel-Escalada, I., Akerman, I., Tena, J.J., Moran, I., Gomez-Marin, C., van de Bunt, M., *et al.* (2014). Pancreatic islet enhancer clusters enriched in type 2 diabetes risk-associated variants. Nat Genet *46*, 136-143.

Quinlan, A.R., and Hall, I.M. (2010). BEDTools: a flexible suite of utilities for comparing genomic features. Bioinformatics *26*, 841-842.

Wolf, F.A., Angerer, P., and Theis, F.J. (2018). SCANPY: large-scale single-cell gene expression data analysis. Genome Biol *19*, 15.

Zhang, Y., Liu, T., Meyer, C.A., Eeckhoute, J., Johnson, D.S., Bernstein, B.E., Nusbaum, C., Myers, R.M., Brown, M., Li, W., *et al.* (2008). Model-based analysis of ChIP-Seq (MACS). Genome Biol *9*, R137.
